# Supplementary material for: Spatial determinants of farmers’ interest in European Union’s pro-investment programs in Poland
Source: PLoS One. 2021 Mar 18;16(3):e0248059. doi: 10.1371/journal.pone.0248059 (PMC7971496; doi:10.1371/journal.pone.0248059)
Supplement: S1 Table — (PDF) [file pone.0248059.s001.pdf]

Municipality	Number of farms	Number of applications 'setting up of young farmers'	Number of applications ‘modernization of agricultural holdings’	Factor score value -farm structure	Factor score value - infrastructure	Factor score value - Organic agriculture	Factor score value - Demography	Factor score value - Animal production	Factor score value - Enetprenuership	Factor score value - Agricultural land greening
Bolesławiec	1438	10	25	-0,15	-0,62	-0,09	0,95	-0,87	0,73	-1,04
Gromadka	618	2	5	-0,55	-1,13	0,59	0,01	-0,55	-0,10	-0,16
Nowogrodziec	1131	7	16	-0,48	-0,32	0,08	0,85	-0,47	-0,13	-0,68
Osiecznica	412	1	5	0,02	-0,86	0,96	0,48	-0,87	0,70	0,45
Warta Bolesławiecka	669	16	48	0,54	0,09	-0,42	0,81	-1,18	-0,10	-1,18
Dzierżoniów	1305	32	73	0,29	-0,44	-0,49	-0,40	-0,94	0,59	-1,57
Łagiewniki	687	10	41	0,61	-0,63	-0,53	-0,34	-1,22	0,11	-1,85
Niemcza	259	1	9	2,25	0,36	-0,55	-1,80	-1,02	1,08	-0,85
Głogów	572	19	31	-1,33	0,03	5,78	1,04	-0,46	-0,04	-1,94
Jerzmanowa	289	4	17	0,29	-0,20	0,00	2,17	-1,20	0,52	-1,46
Kotla	422	8	22	1,17	-0,70	-0,04	0,69	-0,58	-0,39	-0,67
Pęcław	177	5	15	0,87	-0,41	0,49	1,09	-0,67	-0,68	-0,72
Żukowice	336	17	32	1,71	0,01	-0,71	0,63	-1,22	-0,07	-1,52
Góra	1181	36	61	0,72	-0,13	-0,24	-0,58	-0,45	0,61	-0,82
Jemielno	369	7	21	0,89	-0,65	-0,31	-0,33	-0,64	-0,41	-0,39
Niechlów	563	8	16	0,36	-0,76	-0,27	0,31	-0,54	-0,43	-0,60
Wąsosz	773	62	139	0,37	-0,65	-0,34	-0,03	-0,13	0,17	-0,49
Bolków (3)	870	7	24	0,39	-0,40	0,65	-0,89	-0,73	0,90	-0,68
Męcinka (2)	660	17	23	0,69	-0,59	-0,46	-0,13	-1,12	0,26	-1,37
Mściwojów (2)	348	12	37	1,66	-0,10	-0,55	-0,11	-1,12	0,00	-1,74
Paszowice (2)	460	6	41	0,68	-0,26	-0,25	-0,14	-1,09	0,24	-1,27
Wądroże Wielkie (2)	375	16	47	1,56	-0,33	-0,60	-0,22	-1,15	0,21	-1,36
Janowice Wielkie (2)	213	2	9	1,16	-0,11	0,95	-0,40	-0,07	1,97	1,26
Jeżów Sudecki (2)	511	13	10	-0,15	-0,32	0,94	0,30	-0,35	1,49	0,20
Mysłakowice (2)	444	3	2	-0,07	0,24	0,48	-0,90	-0,56	1,04	1,56
Podgórzyn (2)	580	5	6	-0,43	0,41	0,93	-1,43	-0,08	2,41	1,09
Stara Kamienica (2)	339	10	8	0,62	-0,54	1,90	-0,44	-0,55	0,80	0,04
Kamienna Góra (2)	809	18	19	-0,25	-0,19	2,69	0,58	-0,35	-0,18	0,12
Lubawka (3)	708	5	12	-0,29	-0,10	1,49	-1,01	-0,12	0,63	1,19

Marciszów (2)	453	3	7	0,01	-0,34	1,65	-0,41	-0,22	0,19	0,76
Bystrzyca Kłodzka (3)	1632	19	25	-0,20	-0,39	1,10	-1,67	-0,37	1,39	0,33
Kłodzko (2)	1451	21	43	0,16	-0,45	0,41	-0,33	-0,86	0,33	-0,69
Lądek-Zdrój (3)	608	4	3	-0,26	-0,34	0,82	-2,41	-0,21	2,08	1,03
Lewin Kłodzki (2)	284	4	4	0,12	-0,23	0,88	-0,58	-0,14	1,95	1,79
Międzylesie (3)	856	15	27	0,00	-0,52	3,43	-0,66	-0,25	0,00	0,50
Nowa Ruda (2)	1007	10	19	0,12	-0,02	1,31	-1,12	-0,59	0,09	0,51
Radków (3)	682	22	66	0,56	-0,27	-0,14	-0,97	-0,74	0,87	-0,11
Stronie Śląskie (3)	330	1	2	-0,10	-0,47	0,76	-1,91	-0,45	1,50	1,85
Szczytna (3)	671	4	1	-0,46	-0,50	0,57	-1,25	-0,50	0,89	0,69
Chojnów (2)	1320	29	51	0,05	-0,71	-0,41	0,27	-0,55	0,07	-1,02
Krotoszyce (2)	280	9	37	1,39	0,08	-0,53	0,46	-0,88	0,06	-1,38
Kunice (2)	544	4	14	0,19	0,01	-0,36	1,12	-0,67	1,70	-1,75
Legnickie Pole (2)	444	13	30	0,89	-0,19	-0,71	0,06	-0,84	0,31	-1,86
Miłkowice (2)	497	8	26	0,17	-0,22	-0,28	0,43	-0,34	0,89	-1,26
Prochowice (3)	554	7	9	-0,32	-0,38	0,35	-0,28	0,01	1,02	-0,55
Ruja (2)	382	5	20	1,23	-0,42	-0,67	0,00	-1,01	-0,79	-1,65
Leśna (3)	817	11	12	-0,37	-0,20	3,03	-0,90	-0,53	0,45	-0,49
Lubań (2)	979	6	16	-0,23	-0,77	-0,06	0,57	-0,86	0,18	-1,12
Olszyna (3)	412	2	3	-0,01	0,42	0,53	-0,94	-0,53	1,12	-0,50
Platerówka (2)	273	3	4	-0,25	-0,31	0,04	-0,05	-0,50	-0,40	-0,54
Siekierczyn (2)	671	10	8	0,15	0,40	0,02	-0,58	-0,88	-0,03	-1,06
Lubin (2)	1615	14	36	-0,29	-0,36	0,01	1,44	-0,62	1,03	-1,52
Rudna (2)	866	8	37	0,11	-0,27	-0,09	0,92	-1,00	0,21	-0,89
Ścinawa (3)	751	8	35	0,10	-0,85	0,16	-0,21	-0,77	0,38	-1,05
Gryfów Śląski (3)	466	10	18	-0,06	0,63	0,26	-1,62	-0,32	0,92	-0,15
Lubomierz (3)	549	11	17	0,29	-0,63	1,56	-0,49	-0,44	0,34	0,25
Lwówek Śląski (3)	1405	43	34	0,79	-0,09	0,13	-0,55	-0,92	0,92	-0,52
Mirsk (3)	771	8	8	-0,96	-0,83	1,47	-0,64	-0,35	0,79	0,27
Wleń (3)	435	4	15	0,45	-0,72	0,27	-0,89	-0,74	0,95	-0,22
Cieszków (2)	352	16	23	-0,05	-0,58	-0,29	0,20	-0,09	0,41	-0,67
Krośnice (2)	712	11	37	-0,19	-0,55	0,27	-0,03	-0,50	0,32	-0,12
Milicz (3)	1098	46	75	0,47	-0,34	-0,06	-0,26	0,19	1,21	0,08

Bierutów (3)	798	18	38	0,64	-0,82	-0,59	0,41	-1,01	0,49	-1,31
Dobroszyce (2)	603	6	4	-0,39	-0,43	-0,46	0,80	-0,06	0,79	-0,76
Dziadowa Kłoda (2)	572	17	28	0,51	-0,78	-0,78	1,25	-0,35	-0,27	-1,12
Międzybórz (3)	446	8	12	-0,31	-0,59	0,20	0,35	0,12	0,12	-0,22
Oleśnica (2)	1026	35	88	-0,05	-0,85	-0,20	1,68	-0,24	0,57	-1,19
Syców (3)	561	14	19	0,48	0,05	-0,05	-0,05	0,05	0,87	-0,86
Twardogóra (3)	1201	1	23	-0,84	-0,41	1,65	0,41	-0,39	0,36	-0,34
Domaniów (2)	459	21	42	1,71	-0,22	-0,91	-0,17	-1,39	-0,37	-2,15
Jelcz-Laskowice (3)	834	7	10	-0,28	0,21	-0,23	0,37	-0,61	0,74	-0,49
Oława (2)	1844	24	80	0,56	-0,46	-0,54	0,75	-1,29	0,45	-1,96
Chocianów (3)	767	2	4	0,67	-0,52	-0,93	0,24	-1,19	0,38	0,84
Gaworzyce (2)	367	3	25	0,67	-0,35	0,18	0,31	-1,18	0,11	-1,12
Grębocice (2)	596	8	20	0,75	-0,19	-0,55	0,39	-0,98	-0,74	-1,14
Polkowice (3)	503	1	0	-0,04	0,52	0,15	0,26	-0,50	0,64	0,13
Przemków (3)	495	3	18	-0,48	-0,23	1,26	-0,29	-0,40	-0,24	0,34
Radwanice (2)	356	7	13	0,22	-0,54	0,71	1,58	-0,44	0,21	-0,14
Borów (2)	608	29	42	0,70	-0,50	-0,80	-0,06	-1,06	0,09	-1,88
Kondratowice (2)	409	20	36	1,86	-0,39	-0,60	-0,48	-1,46	-0,39	-1,61
Przeworno (2)	418	16	15	1,16	-0,56	-0,55	-1,04	-1,12	-0,28	-1,31
Strzelin (3)	968	17	35	1,03	0,70	-0,83	-0,34	-0,92	0,61	-1,55
Wiązów (3)	982	26	51	0,51	-0,63	-0,36	-0,33	-1,49	-0,02	-1,72
Kostomłoty (2)	917	17	51	0,47	-0,66	-0,73	0,73	-1,02	0,13	-1,77
Malczyce (2)	452	2	16	-0,68	-0,27	-0,24	-0,09	0,26	0,46	-1,35
Miękinia (2)	1389	2	22	-0,15	-0,58	-0,06	1,74	-1,05	1,42	-1,78
Środa Śląska (3)	1144	14	37	0,80	-0,03	-0,54	-0,40	-0,90	1,28	-1,44
Udanin (2)	620	13	61	1,04	-0,70	-0,65	-0,26	-1,21	0,37	-1,67
Dobromierz (2)	465	10	17	1,23	-0,25	-0,44	-0,37	-1,16	0,28	-1,56
Jaworzyna Śląska (3)	369	9	16	1,20	0,21	-0,64	-0,21	-0,97	0,42	-1,61
Marcinowice (2)	765	20	33	0,45	-0,52	-0,64	0,49	-1,16	0,33	-1,64
Strzegom (3)	917	18	48	1,15	1,14	-0,31	-1,33	-0,57	0,97	-1,49
Świdnica (2)	1902	29	86	0,27	-0,41	-0,32	0,49	-0,92	0,60	-1,27
Żarów (3)	710	10	34	0,43	0,40	-0,48	-0,07	-0,96	0,23	-1,82
Oborniki Śląskie (3)	1257	5	23	-0,77	-0,09	-0,08	-0,20	-0,22	1,66	-0,86

Prusice (3)	1013	20	25	-0,38	-0,90	-0,33	0,78	-0,18	0,31	-0,91
Trzebnica (3)	1234	13	50	0,09	0,03	-0,62	0,27	-0,60	1,13	-1,37
Wisznia Mała (2)	652	6	18	-0,54	-0,16	-0,31	1,21	-0,65	1,56	-1,75
Zawonia (2)	684	4	7	-0,10	-0,67	-0,30	1,04	-0,92	0,50	-1,35
Żmigród (3)	1068	35	89	0,52	-0,44	-0,18	-0,05	-0,17	0,49	-0,29
Czarny Bór (2)	326	3	7	-0,65	-0,40	1,90	0,51	-0,23	0,34	0,47
Głuszyca (3)	195	2	6	1,06	1,02	0,83	-1,72	-0,57	1,06	2,78
Mieroszów (3)	369	6	10	0,10	0,15	1,46	-1,74	0,42	1,23	1,55
Stare Bogaczowice (2)	454	6	5	-0,68	-0,40	0,54	-0,87	2,93	1,37	0,40
Walim (2)	461	5	5	-0,37	-0,18	0,81	-1,04	-0,13	1,55	1,32
Brzeg Dolny (3)	338	8	20	0,71	1,03	-0,53	-0,22	-0,46	0,71	-0,92
Wińsko (2)	1035	18	49	-0,05	-1,00	-0,06	-0,47	-0,66	-0,02	-0,69
Wołów (3)	1259	21	60	0,66	-0,21	-0,70	-0,63	-0,80	0,75	0,19
Czernica (2)	537	0	4	-0,08	1,45	0,27	1,82	-0,27	1,73	-0,90
Długołęka (2)	1901	15	32	-0,18	0,17	-0,58	2,38	-0,49	1,75	-1,60
Jordanów Śląski (2)	339	10	12	0,63	-0,46	-0,76	0,58	-1,00	-0,49	-1,93
Kąty Wrocławskie (3)	1419	13	37	0,03	0,41	-0,20	1,46	-0,82	1,41	-1,91
Kobierzyce (2)	650	12	39	0,69	0,50	-0,44	2,49	-0,82	2,33	-2,07
Mietków (2)	539	7	28	0,32	-0,77	-0,68	0,08	-1,06	0,57	-1,84
Sobótka (3)	882	11	32	0,14	-0,04	-0,46	-0,49	-0,80	1,00	-1,72
Żórawina (2)	773	11	26	0,32	-0,51	-0,53	1,71	-1,06	1,42	-2,24
Bardo (3)	426	11	7	0,28	-0,08	-0,41	-1,45	-0,79	0,85	-0,89
Ciepłowody (2)	403	11	12	0,79	-0,39	-0,63	-0,26	-0,79	-0,22	-1,79
Kamieniec Ząbkowicki (2)	671	19	19	0,83	-0,60	-0,46	-0,47	-0,92	0,50	-1,46
Stoszowice (2)	718	14	24	3,07	-0,41	-1,26	-0,75	-1,39	0,70	0,34
Ząbkowice Śląskie (3)	1123	27	53	0,70	0,52	-0,39	-1,72	-0,78	1,64	-1,51
Ziębice (3)	1249	42	21	0,70	-0,23	-0,43	-1,59	-0,75	0,98	-1,18
Złoty Stok (3)	324	5	0	-0,24	-0,60	0,59	-1,92	-0,30	1,57	0,61
Bogatynia (3)	456	3	8	0,62	0,38	-0,39	-0,73	-0,83	0,44	-0,97
Pieńsk (3)	298	4	9	0,78	-0,26	-0,05	-0,38	-0,50	0,89	-0,04
Sulików (2)	547	7	24	0,45	-0,50	-0,37	0,43	-0,90	-0,60	-1,45
Węgliniec (3)	556	6	4	-0,36	-0,94	1,36	-1,03	-0,72	1,03	0,44
Zgorzelec (2)	911	12	28	0,53	-0,39	-0,15	0,03	-1,31	0,74	-1,19

Pielgrzymka (2)	516	11	22	0,69	-0,26	-0,28	0,17	0,01	0,03	-1,14
Świerzawa (3)	775	16	28	0,11	-0,84	0,32	-0,41	-0,81	0,39	-0,42
Zagrodno (2)	565	11	34	1,68	-0,28	-0,60	-0,42	-1,00	-0,47	-1,21
Złotoryja (2)	600	12	63	1,60	0,12	-0,58	-0,05	-1,16	0,57	-1,27
Aleksandrów Kujawski (2)	918	29	111	-0,26	-0,07	-0,31	0,66	0,60	-0,02	-0,73
Bądkowo (2)	665	40	66	0,29	-0,29	-0,66	-0,39	0,54	-0,30	-1,48
Koneck (2)	488	30	68	0,07	0,18	-0,55	-0,75	1,20	-0,42	-0,65
Raciążek (2)	429	13	32	-0,40	0,22	-0,28	0,05	0,41	-0,45	-1,19
Waganiec (2)	430	17	47	0,08	0,28	-0,62	0,50	0,27	-0,93	-0,82
Zakrzewo (2)	448	51	78	0,41	0,01	-0,60	-0,78	2,17	-0,65	-0,48
Bobrowo (2)	682	78	90	0,71	-0,10	0,05	0,58	1,08	-0,61	-0,75
Brodnica (2)	660	42	46	0,05	-0,47	-0,22	2,42	0,11	-0,29	-0,96
Brzozie (2)	422	32	65	0,24	-0,38	-0,15	1,44	1,54	-0,70	0,10
Górzno (3)	445	26	49	-0,07	-0,61	0,01	0,67	1,04	-0,07	-0,15
Jabłonowo Pomorskie (3)	562	58	50	1,06	-0,17	-0,66	0,07	1,36	-0,64	-0,63
Osiek (2)	495	44	53	0,22	-0,09	-0,41	0,35	1,03	-1,04	-0,67
Świedziebnia (2)	709	41	74	0,01	-0,28	-0,61	0,62	1,19	-1,04	-0,15
Zbiczno (2)	427	28	36	0,28	-0,78	-0,22	0,69	0,19	0,74	-0,26
Białe Błota (2)	402	3	5	-1,11	0,19	0,63	2,17	0,35	2,44	0,13
Dąbrowa Chełmińska (2)	520	13	28	-0,33	-0,40	-0,33	1,42	0,11	0,23	-0,16
Dobrcz (2)	721	42	95	0,47	0,00	-0,51	1,17	0,42	0,17	-1,25
Koronowo (3)	1127	111	187	1,41	-0,38	-0,84	0,29	0,34	0,11	-0,99
Nowa Wieś Wielka (2)	385	6	9	-0,49	-0,76	0,27	0,93	0,40	1,37	0,59
Osielsko (2)	372	1	1	-0,78	0,23	0,15	1,91	-0,22	3,03	-0,71
Sicienko (2)	691	24	64	0,78	-0,31	-0,39	1,06	-0,29	0,41	-1,13
Solec Kujawski (3)	226	3	11	-1,23	0,14	2,72	-0,36	4,12	1,82	-0,23
Chełmno (2)	606	36	63	0,38	-0,40	-0,45	2,07	0,13	-0,55	-0,62
Kijewo Królewskie (2)	458	25	50	0,84	-0,10	-0,79	0,58	-0,46	-0,98	-1,45
Lisewo (2)	589	40	32	0,54	-0,13	-0,55	0,54	0,14	-1,04	-1,75
Papowo Biskupie (2)	336	25	46	1,11	0,13	-1,05	0,54	-1,12	-0,87	-1,63
Stolno (2)	393	41	32	1,20	-0,21	-0,69	1,40	-0,15	-0,86	-1,34
Unisław (2)	419	22	45	0,68	-0,03	-0,72	0,37	-0,18	-0,30	-1,18
Ciechocin (2)	578	38	24	-0,15	-0,45	-0,44	0,41	0,87	-0,73	-0,74

Golub-Dobrzyń (2)	1104	68	156	0,04	-0,65	-0,54	1,01	0,33	0,11	-0,87
Kowalewo Pomorskie (3)	998	54	104	0,31	0,07	-0,65	0,05	0,53	0,00	-1,53
Radomin (2)	692	59	78	0,03	0,01	-0,52	-0,49	1,17	-0,68	-1,24
Zbójno (2)	550	41	49	0,36	0,15	-0,65	-0,17	1,03	-0,75	-0,53
Grudziądz (2)	985	34	71	-0,30	0,07	0,23	1,20	-0,27	0,38	-0,99
Gruta (2)	657	36	93	1,04	-0,13	-0,76	0,05	-0,02	-0,67	-1,37
Łasin (3)	667	64	149	1,85	0,10	-0,99	-0,06	0,34	-0,56	-1,42
Radzyń Chełmiński (3)	450	25	73	1,22	-0,04	2,22	0,10	0,30	-0,42	-1,29
Rogóźno (2)	540	20	56	0,61	-0,46	-0,63	1,20	-0,46	-0,71	-1,67
Świecie nad Osą (2)	390	20	52	1,49	-0,15	-0,47	0,49	-0,09	-1,37	-1,06
Dąbrowa Biskupia (2)	554	52	107	0,67	-0,46	-0,66	0,76	0,89	-0,45	-0,73
Gniewkowo (3)	520	51	113	1,27	0,08	-0,71	-0,44	0,23	-0,28	-1,13
Inowrocław (2)	956	67	133	0,15	-0,31	0,22	-0,02	0,35	0,38	-1,57
Janikowo (3)	446	42	94	1,85	0,69	-0,82	-0,83	-0,04	-0,48	-1,29
Kruszwica (3)	903	87	181	3,84	0,73	-0,54	-1,05	0,42	-0,29	1,34
Pakość (3)	359	21	34	0,53	0,18	-0,61	-0,11	0,36	0,03	-1,01
Rojewo (2)	535	23	87	0,85	-0,35	-0,46	0,32	0,57	-0,64	-0,24
Złotniki Kujawskie (2)	575	52	77	1,25	0,09	-0,61	0,23	0,50	-0,24	-0,73
Bobrowniki (2)	328	5	10	-0,09	-0,80	-0,12	0,63	-0,27	-0,28	0,95
Chrostkowo (2)	481	32	63	0,21	-0,25	-0,51	-0,47	0,87	-0,92	-0,28
Dobrzyń nad Wisłą (3)	1178	55	88	-0,04	-0,10	-0,47	-0,04	-0,49	-0,62	-1,14
Kikół (2)	740	52	67	0,14	-0,20	-0,65	0,27	0,66	-0,82	-0,81
Lipno (2)	1470	64	86	-0,64	-0,52	-0,07	1,03	0,93	-0,42	-0,26
Skępe (3)	810	12	15	-0,38	-0,92	-0,14	0,37	0,08	0,50	0,79
Tłuchowo (2)	598	36	76	0,02	-0,58	0,15	0,86	0,66	-0,42	-0,03
Wielgie (2)	1073	34	50	-0,38	-0,64	-0,37	0,80	0,18	-0,52	-0,24
Dąbrowa (2)	686	44	101	0,48	-0,42	-0,62	0,65	-0,04	-0,57	-1,10
Jeziora Wielkie (2)	647	37	93	-0,14	-0,74	-0,38	-0,16	0,38	0,21	-0,93
Mogilno (3)	1781	115	232	0,39	0,09	-0,41	-0,11	-0,02	0,06	-1,02
Strzelno (3)	1046	52	152	0,77	-0,02	-0,72	-0,59	-0,49	-0,49	-1,56
Kcynia (3)	925	65	184	1,49	-0,08	-0,59	0,08	0,71	-0,77	-0,16
Mrocza (3)	403	24	114	2,31	-0,31	-0,93	0,49	0,24	-0,47	-0,62
Nakło nad Notecią (3)	606	36	84	2,27	1,16	-0,54	-0,58	0,24	0,05	-0,15

Sadki (2)	590	28	52	1,53	-0,43	-0,83	0,97	0,08	-0,97	-0,54
Szubin (3)	822	59	120	1,33	-0,14	0,47	0,62	0,59	-0,08	0,19
Bytoń (2)	545	37	54	0,04	-0,28	-0,41	-0,89	0,47	-0,61	-0,65
Dobre (2)	624	31	88	0,05	-0,05	-0,56	-0,67	0,93	-0,50	-1,01
Osięciny (2)	834	43	129	0,58	-0,05	-0,87	-0,51	1,29	-0,48	-1,04
Piotrków Kujawski (3)	1066	48	201	-0,23	-0,41	-0,44	-0,13	0,86	-0,09	-0,51
Radziejów (2)	620	45	140	0,60	-0,39	-0,77	-0,29	0,02	-0,38	-1,48
Topólka (2)	863	49	107	-0,43	-0,53	-0,44	-0,27	0,66	-0,52	-0,53
Brzuze (2)	801	41	116	0,02	-0,10	-0,33	-0,40	0,37	-0,70	-0,87
Rogowo (2)	848	45	101	-0,22	-0,70	-0,54	0,28	0,46	-0,12	0,66
Rypin (2)	960	59	128	-0,17	-0,28	-0,45	0,72	1,13	-0,87	-0,27
Skrwilno (2)	1057	29	57	-0,39	-0,52	-0,43	-0,60	0,94	-0,53	0,70
Wąpielsk (2)	492	44	57	0,53	0,06	-0,35	-0,62	0,88	-0,95	-0,70
Kamień Krajeński (3)	544	50	76	1,15	-0,46	-0,58	0,45	0,06	-0,11	-0,30
Sępólno Krajeńskie (3)	697	42	149	1,09	0,10	-0,50	-0,18	0,72	-0,15	0,02
Sośno (2)	547	48	69	1,52	-0,51	-0,82	0,59	0,63	-0,37	-0,52
Więcbork (3)	731	67	85	1,02	-0,30	-0,52	-0,19	1,04	-0,15	0,27
Bukowiec (2)	459	30	77	1,10	-0,38	-0,55	0,82	0,92	-0,73	-0,37
Dragacz (2)	508	15	32	-0,03	-0,62	-0,36	0,59	0,16	0,58	-0,65
Drzycim (2)	353	25	55	0,27	-0,57	-0,19	0,68	1,02	-0,55	-0,19
Jeżewo (2)	510	13	29	-0,05	-0,53	-0,30	0,78	0,78	-0,34	-0,28
Lniano (2)	475	23	51	0,13	-0,27	-0,25	0,45	0,50	-0,66	0,23
Nowe (3)	378	10	17	0,65	0,11	0,19	-0,49	-0,08	-0,49	-0,35
Osie (2)	312	7	10	0,62	-1,08	-0,70	1,15	-0,13	0,25	-0,21
Pruszcz (2)	779	58	147	0,49	-0,16	-0,61	0,49	0,92	-0,09	-1,26
Świecie (3)	871	23	77	0,52	1,18	-0,26	-0,53	0,16	0,40	-0,78
Świekatowo (2)	296	21	38	0,34	-0,04	-0,31	0,63	1,55	-0,34	-0,31
Warlubie (2)	513	13	40	8,68	-0,90	-8,21	0,27	-4,95	0,56	10,49
Chełmża (2)	800	54	137	1,31	-0,11	-0,93	0,28	0,40	-0,64	-1,32
Czernikowo (2)	673	25	48	0,07	-0,65	-0,33	1,00	0,26	-0,08	-0,52
Lubicz (2)	818	22	38	-0,32	0,42	-0,19	1,34	0,25	0,88	-1,05
Łubianka (2)	581	35	67	0,13	-0,08	-0,61	1,86	0,54	-0,12	-1,14
Łysomice (2)	638	26	66	0,68	0,02	-0,56	1,42	-0,13	1,13	-1,48

Obrowo (2)	817	40	72	-0,28	-0,48	-0,46	3,04	0,58	0,67	-0,45
Wielka Nieszawka (2)	122	2	6	-0,73	-1,02	-0,24	0,90	-0,58	2,05	-0,09
Zławieś Wielka (2)	929	25	85	0,23	-0,30	-0,61	1,86	0,49	0,64	-0,93
Cekcyn (2)	786	16	24	-0,44	-0,89	-0,08	1,05	-0,26	-0,08	0,55
Gostycyn (2)	373	38	54	1,95	-0,30	-1,03	0,44	0,73	0,10	-0,67
Kęsowo (2)	370	42	41	2,05	0,07	-0,74	0,56	1,31	-0,89	0,29
Lubiewo (2)	794	30	55	-0,28	-0,75	-0,54	0,93	0,34	-0,10	-0,05
Śliwice (2)	452	7	11	-0,18	-0,98	-0,50	1,01	-0,09	-0,11	1,32
Tuchola (3)	843	40	43	0,32	-0,03	-0,39	-0,09	0,07	0,38	-0,01
Dębowa Łąka (2)	394	34	69	0,10	0,10	-0,47	-0,20	1,12	-0,53	-0,52
Książki (2)	561	13	31	0,12	-0,30	-0,38	-0,11	0,49	-0,83	-0,84
Płużnica (2)	468	32	77	1,46	-0,09	-0,89	0,22	0,69	-0,59	-1,22
Ryńsk (2)	969	0	0	1,10	-0,32	-0,51	0,67	0,52	-0,39	-0,72
Baruchowo (2)	542	24	42	-0,42	-0,91	-0,46	-0,27	0,17	0,05	0,13
Boniewo (2)	464	30	73	0,36	-0,34	-0,45	-0,66	0,08	-0,98	-0,94
Brześć Kujawski (3)	673	54	111	1,17	0,22	-0,67	-0,66	-0,22	-0,04	-1,19
Choceń (2)	658	38	80	-0,15	-0,10	-0,52	0,09	-0,15	-0,54	-1,43
Chodecz (3)	791	37	67	0,16	-0,69	-0,63	0,06	0,42	-0,48	-0,97
Fabianki (2)	723	15	28	-0,90	0,54	0,08	0,66	0,11	0,40	-0,90
Izbica Kujawska (3)	893	59	92	-0,07	-0,36	-0,27	-0,40	0,74	-0,50	-0,29
Kowal (2)	621	35	68	0,04	-0,43	-0,52	-0,52	-0,06	-0,41	-0,44
Lubanie (2)	654	7	49	-0,31	-0,16	-0,54	-0,24	-0,31	-0,27	-1,35
Lubień Kujawski (3)	881	46	101	0,15	-0,39	-0,62	-0,34	0,52	-0,73	-0,62
Lubraniec (3)	1047	67	219	0,51	-0,09	-0,63	-0,80	0,16	-0,48	-1,17
Włocławek (2)	707	30	93	-0,09	-0,74	-0,46	0,60	-0,61	0,39	-0,36
Barcin (3)	510	38	62	1,12	0,44	-0,44	-0,58	0,57	-0,17	-0,25
Gąsawa (2)	471	42	80	1,14	-0,70	-1,06	-0,39	0,21	0,67	-0,57
Janowiec Wielkopolski (3)	491	74	162	1,65	0,08	-0,92	-0,29	2,41	-0,12	-0,75
Łabiszyn (3)	574	31	64	0,60	-0,20	-0,16	0,70	1,01	0,16	0,41
Rogowo (2)	548	26	32	1,10	-0,40	-0,68	0,02	0,98	-0,40	-0,53
Żnin (3)	983	149	235	1,73	0,45	-0,94	-0,45	1,13	-0,17	-0,87
Biała Podlaska (2)	2599	171	232	-0,88	-0,88	1,70	1,46	-0,41	0,11	-0,17
Drelów (2)	1146	67	86	-0,38	-0,84	0,43	0,38	0,29	-0,85	0,64

Janów Podlaski (2)	867	58	92	-0,35	-0,89	0,50	-0,17	-0,20	-0,12	0,24
Kodeń (2)	758	19	47	-0,34	-0,85	0,98	-1,19	-0,20	-0,65	0,25
Konstantynów (2)	706	37	119	-0,56	-0,55	0,07	0,43	-0,20	-0,47	-0,32
Leśna Podlaska (2)	695	58	96	-0,31	-0,56	-0,02	-0,26	0,10	-0,68	-0,03
Łomazy (2)	1032	87	131	0,12	-0,69	0,47	-1,18	0,43	-0,62	0,61
Międzyrzec Podlaski (2)	2070	99	258	-0,74	-0,73	0,20	0,64	0,60	-0,35	0,20
Piszczac (2)	1307	69	96	-0,55	-0,42	1,10	0,04	-0,10	-0,55	0,31
Rokitno (2)	649	29	59	0,02	-0,81	0,37	-0,68	-0,28	-0,82	-0,04
Rossosz (2)	562	37	35	-0,03	-0,67	-0,09	-1,44	-0,46	-0,16	0,87
Sławatycze (2)	425	13	21	-0,26	-0,36	2,26	-1,04	0,12	-0,47	0,08
Sosnówka (2)	614	78	163	0,15	-0,73	1,17	-0,77	0,96	-1,06	0,29
Terespol (2)	1135	27	40	-0,81	-0,62	0,57	0,02	-0,46	-0,68	-0,14
Tuczna (2)	837	51	100	-0,04	-0,75	0,39	-1,50	0,39	-0,94	0,43
Wisznice (2)	951	112	226	-0,20	-0,73	-0,05	0,01	-0,01	-0,32	-0,09
Zalesie (2)	1005	19	59	-0,93	-0,86	1,62	0,68	-0,28	-0,72	-0,07
Aleksandrów (2)	728	11	43	-0,44	-0,73	-0,72	0,38	-1,34	-0,20	0,68
Biłgoraj (2)	2663	15	14	-0,63	-0,50	-0,94	0,80	-1,00	-0,03	1,45
Biszcza (2)	828	25	84	-0,41	-0,74	-0,10	0,20	-0,65	0,05	-0,48
Frampol (3)	1461	10	24	-0,94	-0,42	-0,11	-1,38	-0,51	-0,15	-0,09
Goraj (2)	986	14	17	-1,07	-0,69	-0,50	-1,01	-0,47	-0,57	-0,39
Józefów (3)	1338	5	17	-0,68	-0,48	-0,55	-1,05	-0,93	0,18	0,41
Księżpol (2)	1521	15	58	-0,67	-0,64	-0,17	0,69	-0,84	-0,17	-0,28
Łukowa (2)	807	37	85	-0,18	-1,11	-0,73	0,32	-1,02	-0,04	-0,50
Obsza (2)	978	30	93	-0,39	-0,57	0,48	0,14	-0,83	-1,07	-0,92
Potok Górny (2)	1207	23	123	-0,20	-0,28	-0,73	-0,28	-1,03	-0,62	0,13
Tarnogród (3)	1143	45	64	-0,47	-0,06	0,13	-0,41	-0,66	-0,12	-0,21
Tereszpol (2)	1163	4	16	-0,75	-1,09	-0,89	-0,02	-1,16	-0,04	1,06
Turobin (2)	1537	55	89	-0,55	-0,60	-0,64	-2,54	-0,33	-0,37	-0,85
Białopole (2)	759	29	38	-0,09	-0,54	-0,14	-1,53	-0,34	-0,58	-0,44
Chełm (2)	2224	79	145	-0,40	-0,31	0,38	0,81	-0,85	-0,05	-1,21
Dorohusk (2)	1547	17	16	-0,46	-0,90	-0,05	-0,73	-0,83	-0,43	0,22
Dubienka (2)	521	19	30	-0,25	-1,01	-0,27	-0,72	-0,50	-0,37	0,39
Kamień (2)	934	22	45	-0,02	-0,66	-0,30	1,33	-0,65	-0,57	-0,47

Leśniowice (2)	939	38	47	-0,06	-0,60	-0,15	-0,75	-0,65	-0,78	-1,10
Rejowiec Fabryczny (2)	1049	8	9	-0,87	-0,32	-0,11	-0,64	-0,51	-0,98	0,11
Ruda-Huta (2)	1020	11	26	-0,86	-0,76	0,21	-0,31	-0,52	-0,85	0,18
Sawin (2)	1248	29	48	-0,51	-0,92	0,40	0,03	-0,05	-0,47	0,07
Wierzbica (2)	865	22	40	0,17	-0,68	-0,32	-0,27	-0,67	-0,79	-0,54
Wojsławice (2)	951	30	18	-0,42	-0,69	0,01	-1,36	-0,63	-0,70	-0,77
Żmudź (2)	684	30	81	0,19	-0,93	-0,43	-0,07	-0,62	-0,96	-0,57
Rejowiec (2)	982	18	21	-0,45	-0,60	-0,26	-0,84	-0,80	-0,56	-0,98
Dołhobyczów (2)	891	52	90	0,99	-0,55	-0,74	-1,36	-1,04	-0,72	-1,55
Horodło (2)	1031	29	61	-0,03	-0,45	-0,53	-1,20	-0,81	-0,57	-1,63
Hrubieszów (2)	2334	85	203	-0,02	-0,33	-0,51	-0,94	-0,78	-0,88	-1,62
Mircze (2)	1452	68	91	0,26	-0,67	-0,61	-2,07	-0,71	-0,50	-1,18
Trzeszczany (2)	847	34	37	0,13	-0,30	-0,60	-1,96	-0,67	-0,87	-1,31
Uchanie (2)	989	38	78	0,08	-0,46	-0,37	-2,31	-0,51	-0,83	-1,04
Werbkowice (2)	1612	61	80	-0,04	-0,35	-0,54	-1,06	-0,67	-0,75	-1,08
Batorz (2)	702	34	46	-0,57	-0,37	-0,67	-0,91	-0,41	-0,44	-0,81
Chrzanów (2)	718	31	93	-0,75	-0,66	-0,57	-0,84	-0,25	-0,58	-0,72
Dzwola (2)	1410	18	38	-0,59	-0,83	-0,82	-0,82	-0,78	-0,51	0,45
Godziszów (2)	1226	54	128	-0,48	-0,30	-0,51	-1,36	-0,48	-0,38	-0,31
Janów Lubelski (3)	1728	4	12	-0,89	-0,63	-0,54	-0,34	-0,61	1,23	0,59
Potok Wielki (2)	874	14	53	-0,55	-0,19	-0,48	-0,60	-0,67	-0,57	-1,04
Fajsławice (2)	1153	33	58	-0,89	-0,19	-0,49	-1,16	-0,58	-0,57	-1,39
Gorzków (2)	915	31	57	-0,47	-0,60	-0,58	-2,86	-0,45	-0,38	-1,10
Izbica (2)	1644	20	31	-0,70	-0,24	-0,32	-1,09	-0,51	-0,62	-0,73
Krasnystaw (2)	1636	42	103	-0,26	0,14	-0,11	-1,18	-0,57	-0,73	-1,00
Kraśniczyn (2)	796	17	20	-0,43	-0,25	0,00	-3,10	-0,59	-0,67	-1,03
Łopiennik Górny (2)	971	35	50	-0,95	-0,72	-0,47	-0,85	-0,38	-0,53	-1,04
Rudnik (2)	662	30	18	-0,30	-0,40	-0,25	-2,50	-0,22	-0,98	-0,95
Siennica Różana (2)	769	26	31	-0,20	-0,50	-0,38	-0,94	-0,62	-0,10	-1,20
Żółkiewka (2)	1194	48	47	-0,31	-0,50	-0,32	-2,74	-0,29	-0,36	-0,92
Annopol (3)	1713	22	58	-1,06	-0,54	-0,38	-0,49	-0,51	-0,46	-0,53
Dzierzkowice (2)	1383	20	43	-1,02	-0,08	-0,28	-0,53	-0,70	-0,50	-1,04
Gościeradów (2)	1633	11	46	-0,80	-0,55	-0,29	-0,69	-0,72	-0,49	-0,70

Kraśnik (2)	1622	20	87	-1,08	-0,42	-0,26	0,50	-0,83	-0,14	-1,20
Szastarka (2)	1318	23	40	-0,95	0,26	-0,31	-1,21	-0,25	-0,58	-0,97
Trzydnik Duży (2)	1447	42	102	-0,41	0,25	-0,61	-1,03	-0,60	-0,78	-1,43
Wilkołaz (2)	1287	26	69	-0,64	0,07	-0,45	-0,24	-0,45	-0,72	-1,30
Zakrzówek (2)	1438	41	71	-0,70	0,01	-0,42	-1,21	-0,27	-0,65	-1,01
Abramów (2)	934	20	50	-0,61	-0,41	-0,30	-0,82	0,15	-0,74	-0,09
Firlej (2)	1489	13	29	-1,11	-0,51	0,48	-0,18	-0,35	-0,48	0,50
Jeziorzany (2)	727	6	35	-1,03	-0,70	0,05	-0,44	-0,30	-0,68	0,26
Kamionka (2)	1336	16	32	-1,00	-0,69	-0,11	0,05	-0,13	0,02	0,00
Kock (3)	1018	9	25	-1,02	-0,30	0,31	-0,27	-0,05	-0,01	0,12
Lubartów (2)	1848	10	20	-1,36	-0,44	0,28	1,24	-0,55	0,03	-0,17
Michów (2)	1557	17	43	-1,04	-0,58	0,39	-0,97	-0,22	-0,49	0,15
Niedźwiada (2)	1468	11	28	-0,91	-0,46	-0,08	-0,43	-0,66	-0,58	0,18
Ostrów Lubelski (3)	1344	22	31	-1,00	-0,68	-0,03	-0,63	-0,10	-0,29	0,16
Ostrówek (2)	896	8	33	-0,91	-0,47	2,10	-0,61	-0,19	-0,78	0,16
Serniki (2)	948	13	10	-0,94	-0,39	0,34	0,17	-0,47	-0,62	-0,09
Uścimów (2)	779	21	72	-0,61	-0,70	0,23	-0,82	0,59	-0,38	0,26
Bełżyce (3)	2258	66	169	-0,49	0,44	-0,45	-0,26	-0,62	-0,08	-1,00
Borzechów (2)	854	48	100	-0,57	-0,24	-0,57	-0,14	-0,43	-0,54	-0,99
Bychawa (3)	2014	68	170	-0,52	-0,26	-0,57	-0,89	-0,39	-0,29	-1,40
Garbów (2)	1760	26	75	-0,91	-0,23	-0,41	0,57	-0,29	-0,35	-0,91
Głusk (2)	1155	14	24	-0,90	1,61	-0,27	1,61	-0,32	0,32	-1,71
Jabłonna (2)	1478	21	55	-0,60	-0,12	-0,28	-0,21	-0,80	-0,22	-1,50
Jastków (2)	2498	22	73	-0,93	0,45	-0,25	0,43	-0,57	0,34	-1,67
Konopnica (2)	2323	20	78	-1,02	0,92	-0,11	0,57	-0,56	0,81	-1,77
Krzczonów (2)	940	53	62	-0,04	-0,56	-0,44	-1,54	-0,30	-0,18	-1,59
Niedrzwica Duża (2)	1513	39	118	-0,25	0,88	-0,42	0,33	-0,60	-0,10	-1,16
Niemce (2)	3566	16	77	-1,38	0,25	-0,19	1,11	-0,28	0,40	-1,27
Strzyżewice (2)	1589	20	70	-0,73	0,12	-0,33	0,07	-0,65	0,07	-1,46
Wojciechów (2)	1543	27	132	-0,74	0,12	-0,50	0,25	-0,74	-0,56	-1,39
Wólka (2)	1545	4	25	-1,11	0,98	-0,15	1,92	-0,40	0,06	-1,18
Wysokie (2)	1203	39	71	-0,45	-0,49	-0,47	-2,03	-0,11	-0,68	-1,19
Zakrzew (2)	801	30	29	-0,75	-0,58	-0,63	-1,35	-0,24	-0,75	-1,17

Cyców (2)	1483	21	73	-0,40	-0,37	0,52	0,64	-0,21	-1,04	-0,43
Ludwin (2)	1077	19	70	-1,00	-0,67	0,22	1,62	-0,39	-0,53	-0,44
Łęczna (3)	1288	22	28	-0,04	1,97	0,05	0,78	-0,59	-1,03	-1,00
Milejów (2)	1560	9	22	-0,94	-0,26	-0,25	-0,58	-0,44	-0,60	-0,76
Puchaczów (2)	1134	6	24	-0,62	0,00	-0,11	0,76	-0,63	-0,26	-0,62
Spiczyn (2)	1003	11	27	-1,04	-0,29	-0,36	0,72	-0,37	-0,16	-0,60
Adamów (2)	961	5	14	-0,74	-0,63	-0,37	-0,23	-0,78	-0,32	0,13
Krzywda (2)	1748	26	142	-0,72	-0,48	-0,51	0,90	-0,09	-0,45	0,44
Łuków (2)	3272	35	161	-0,93	-0,69	-0,11	1,93	-0,30	-0,31	0,04
Serokomla (2)	850	23	74	-0,78	-0,61	-0,46	0,05	0,18	-0,71	0,13
Stanin (2)	1845	38	285	-0,59	-0,35	-0,74	0,78	0,24	-0,45	0,32
Stoczek Łukowski (2)	1922	14	83	-0,73	-0,43	-0,56	-0,41	-0,19	-0,36	0,59
Trzebieszów (2)	1426	46	244	-0,51	-0,55	-0,19	0,55	0,59	-0,69	0,24
Wojcieszków (2)	1373	26	93	-0,76	-0,37	-0,53	0,47	0,10	-0,86	-0,06
Wola Mysłowska (2)	1124	44	232	-0,53	-0,26	-0,59	-0,08	0,66	-0,24	0,58
Chodel (2)	1605	36	77	-1,13	-0,50	-0,47	0,31	-0,48	-0,12	-0,67
Józefów nad Wisłą (2)	1634	51	173	-1,02	-0,72	-0,45	-1,05	-0,74	-0,20	-0,48
Karczmiska (2)	1317	18	55	-0,86	-0,08	-0,34	-0,75	-0,74	-0,70	-0,40
Łaziska (2)	1423	45	230	-1,21	-0,74	-0,32	-0,46	-0,75	-0,50	-0,70
Opole Lubelskie (3)	2886	49	227	-1,00	-0,17	-0,44	-0,65	-0,59	0,24	-0,40
Poniatowa (3)	1548	33	67	-0,68	0,85	-0,26	-1,03	-0,45	-0,08	-1,01
Wilków (2)	1217	16	107	-1,16	-0,45	-0,39	-1,31	-0,73	-0,56	-0,47
Dębowa Kłoda (2)	848	50	69	0,07	-1,01	-0,21	-0,31	-0,50	0,25	-0,02
Jabłoń (2)	769	82	204	0,23	-0,42	-0,43	-0,30	0,64	-0,60	-0,06
Milanów (2)	597	80	114	0,24	-0,42	-0,24	-0,42	0,67	-0,79	-0,07
Parczew (3)	1351	76	117	-0,54	-0,49	0,00	-0,45	0,07	0,57	-0,18
Podedwórze (2)	469	53	122	0,33	-0,89	-0,33	-0,82	0,32	-0,12	-0,20
Siemień (2)	1110	26	55	-0,75	-0,57	0,01	-1,02	-0,13	-0,39	0,21
Sosnowica (2)	572	12	26	-0,53	-1,05	0,89	-0,92	-0,24	0,10	0,79
Baranów (2)	1002	18	35	-0,75	-0,61	-0,08	-1,60	-0,42	-0,55	0,49
Janowiec (2)	907	7	23	-1,04	-0,94	-0,16	-0,47	-0,59	0,47	-0,02
Kazimierz Dolny (3)	1406	8	32	-0,89	0,53	-0,77	-1,87	-0,54	1,43	0,11
Końskowola (2)	1963	23	159	-0,85	0,38	0,03	-1,15	-0,68	-0,13	-0,84

Kurów (2)	1286	32	131	-0,86	-0,23	-0,40	-0,36	-0,49	0,43	-0,94
Markuszów (2)	629	13	31	-0,64	0,05	-0,28	-0,71	-0,28	-0,50	-0,39
Nałęczów (3)	1247	12	77	-0,52	1,40	-0,35	-1,52	-0,46	0,76	-1,21
Puławy (2)	2414	16	64	-1,26	-0,35	-0,20	-0,06	-0,58	-0,01	-0,50
Wąwolnica (2)	858	14	44	-0,72	0,26	-0,04	-1,05	-0,66	-0,21	-0,78
Żyrzyn (2)	1274	14	55	-0,86	-0,39	-0,08	-0,62	-0,51	-0,37	0,13
Borki (2)	1146	51	54	-0,73	-0,47	-0,22	0,26	-0,05	-0,55	0,11
Czemierniki (2)	1050	26	33	-0,74	-0,74	0,21	-0,55	-0,05	-0,52	0,01
Kąkolewnica (2)	1711	60	70	-0,84	-0,56	-0,36	0,56	-0,07	-0,33	0,27
Komarówka Podlaska (2)	984	56	85	-0,40	-0,80	-0,41	-1,09	0,33	-0,47	0,18
Radzyń Podlaski (2)	1614	56	95	-0,87	-0,59	-0,13	0,40	-0,12	-0,31	-0,34
Ulan-Majorat (2)	1167	59	174	-0,44	-0,24	-0,44	0,16	0,21	-0,95	0,06
Wohyń (2)	1231	96	128	-0,39	-0,47	-0,15	-0,48	0,62	-0,75	-0,07
Kłoczew (2)	1494	43	233	-0,82	-0,47	-0,67	0,34	0,18	-0,71	0,30
Nowodwór (2)	981	7	73	-0,77	-0,34	0,20	0,28	-0,41	-1,06	0,24
Ryki (3)	2928	24	116	-1,05	0,43	-0,09	-0,48	0,13	0,19	0,01
Stężyca (2)	1258	15	70	-1,00	-0,65	-0,43	-0,56	-0,09	-0,11	0,47
Ułęż (2)	823	10	21	-1,13	-0,84	-0,04	-0,81	-0,33	-0,23	0,21
Mełgiew (2)	1649	17	50	-0,76	0,31	-0,31	0,26	-0,65	0,18	-1,75
Piaski (3)	2270	95	255	-0,41	-0,38	-0,61	-0,80	-0,70	-0,06	-1,73
Rybczewice (2)	1027	43	166	1,31	-0,14	-0,73	-2,92	-2,71	0,12	-1,03
Trawniki (2)	1555	14	23	-0,94	0,11	-0,07	-0,69	-0,58	-0,70	-0,72
Bełżec (2)	697	6	0	-0,92	-0,27	-0,25	0,13	-0,97	-0,40	-0,37
Jarczów (2)	1116	18	38	-0,51	-0,68	-0,59	-1,29	-0,82	-0,50	-1,29
Krynice (2)	1062	13	12	-0,75	-0,09	1,64	-1,84	-0,68	-0,57	-1,22
Łaszczów (3)	1174	31	69	-0,26	-0,27	-0,31	-1,99	-0,70	-0,64	-1,29
Rachanie (2)	1138	16	26	-0,65	-0,39	-0,54	-1,06	-0,54	-0,54	-1,07
Susiec (2)	1503	7	18	-0,91	-0,83	-0,75	-0,21	-0,71	0,20	0,11
Tarnawatka (2)	718	7	10	-0,36	-0,54	-0,39	-0,02	-0,59	-0,64	-0,87
Telatyn (2)	896	54	114	0,29	-0,41	-0,66	-1,69	-0,51	-0,50	-1,67
Tomaszów Lubelski (2)	3007	23	42	-0,85	-0,26	0,11	0,00	-0,96	-0,28	-0,68
Tyszowce (3)	1614	26	31	-0,51	-0,54	-0,41	-1,16	-0,59	-0,35	-0,90
Ulhówek (2)	746	36	67	0,28	-0,68	-0,61	-0,91	-0,76	-1,02	-1,38

Hanna (2)	760	70	116	0,11	-0,46	1,19	-1,41	0,56	-0,77	0,64
Hańsk (2)	586	25	39	0,10	-0,87	1,08	0,45	0,27	-0,88	1,08
Stary Brus (2)	554	17	25	-0,54	-1,17	-0,23	-0,05	-0,26	-0,15	0,69
Urszulin (2)	1203	19	56	-0,91	-0,92	1,39	0,45	-0,03	-0,24	0,45
Włodawa (2)	947	51	95	-0,44	-0,90	4,29	-0,12	-0,25	0,77	0,33
Wola Uhruska (2)	593	17	24	-0,46	-0,85	-0,12	-1,10	-0,52	-0,37	0,43
Wyryki (2)	538	26	65	-0,14	-1,07	-0,23	-0,66	-0,42	-0,35	0,49
Adamów (2)	1252	5	43	-0,84	-0,54	-0,49	-0,55	-0,37	-0,17	-0,21
Grabowiec (2)	1000	29	45	0,12	-0,77	-0,68	-1,62	-0,63	-0,83	-1,34
Komarów-Osada (2)	1347	50	79	-0,16	-0,21	-0,41	-1,48	-0,60	-0,59	-0,95
Krasnobród (3)	1319	3	4	-0,73	-0,62	-0,87	-0,45	-0,81	0,65	0,72
Łabunie (2)	1108	14	45	-0,46	-0,21	0,26	0,08	-0,85	-0,43	-0,89
Miączyn (2)	1032	39	68	0,34	-0,45	-0,39	-1,41	-0,94	-1,13	-1,04
Nielisz (2)	1399	18	33	-0,79	-0,60	-0,39	-1,86	-0,71	-0,66	-1,06
Radecznica (2)	1408	20	20	-0,77	-0,33	-0,52	-2,38	-0,25	-0,60	-0,45
Sitno (2)	1153	34	59	-0,09	0,10	-0,29	-0,30	-0,51	-0,60	-0,91
Skierbieszów (2)	1400	31	46	-0,59	-0,72	-0,45	-1,32	-0,69	-0,30	-1,20
Stary Zamość (2)	1086	11	22	-0,60	-0,13	-0,25	-0,77	-0,36	-0,54	-0,71
Sułów (2)	886	15	34	-0,37	-0,18	-0,44	-1,73	-0,58	-0,77	-1,30
Szczebrzeszyn (3)	1777	9	24	-0,57	-0,20	-0,51	-1,21	-0,92	-0,41	-0,93
Zamość (2)	2309	62	131	-0,26	0,33	-0,33	0,15	-0,86	0,16	-1,02
Zwierzyniec (3)	580	2	3	-0,77	-0,60	-0,50	-1,31	-0,73	0,63	0,58
Bogdaniec (2)	812	12	52	-0,60	-0,19	0,75	0,37	0,48	0,80	-0,22
Deszczno (2)	895	13	44	-1,12	-0,66	0,84	1,45	0,96	1,30	0,02
Kłodawa (2)	574	67	136	0,85	-0,98	-1,56	1,48	-1,69	2,38	2,06
Lubiszyn (2)	731	18	50	0,13	-0,59	0,76	0,24	0,14	0,37	0,01
Santok (2)	818	9	71	-0,13	-0,42	0,66	0,96	-0,55	0,82	-0,03
Witnica (3)	1053	25	59	-0,22	-0,53	1,42	0,05	-0,42	0,33	0,29
Bobrowice (2)	412	4	16	-0,74	-1,02	4,60	0,43	-0,41	0,47	-0,37
Bytnica (2)	225	3	5	0,54	-0,75	4,74	-0,58	-0,86	-0,09	-0,42
Dąbie (2)	408	59	90	-0,08	-0,51	4,30	0,37	-0,19	-0,10	0,04
Gubin (2)	1012	15	22	0,20	-1,06	0,09	0,25	-0,91	0,14	0,03
Krosno Odrzańskie (3)	727	4	5	1,35	-0,35	-0,28	-0,69	-1,07	1,51	1,68

Maszewo (2)	412	28	102	-0,70	-0,80	5,18	-0,27	-0,48	-0,29	-0,93
Bledzew (2)	457	12	43	1,13	-0,96	0,11	-0,42	-0,94	0,49	-0,50
Międzyrzecz (3)	575	16	41	1,86	-0,34	-0,82	-0,21	-0,78	1,71	1,24
Przytoczna (2)	418	11	58	1,69	-0,62	0,12	-0,34	-0,26	0,70	-0,07
Pszczew (2)	356	15	62	0,46	-0,75	2,97	0,08	0,81	1,05	0,30
Skwierzyna (3)	465	9	28	0,27	-0,73	0,54	-0,76	-0,03	1,44	0,21
Trzciel (3)	581	9	23	-0,08	-0,91	1,32	-0,16	-0,22	0,99	-0,31
Bytom Odrzański (3)	344	9	5	-0,60	0,20	0,25	-0,20	1,20	0,61	-0,88
Kolsko (2)	290	8	6	-0,28	-0,71	0,56	-0,02	-0,12	-0,05	0,40
Kożuchów (3)	1033	11	11	-0,16	-0,49	0,30	-0,48	-0,42	0,80	-0,58
Nowa Sól (2)	354	21	26	0,23	-0,37	2,79	-0,05	-0,09	0,87	0,24
Nowe Miasteczko (3)	308	15	19	2,28	-0,16	0,05	-0,31	-1,32	0,06	-0,15
Otyń (2)	355	3	8	-0,50	0,03	2,19	0,31	1,17	0,64	-0,44
Siedlisko (2)	215	3	2	0,85	-0,62	0,11	1,05	0,09	0,12	-0,95
Cybinka (3)	453	14	13	1,13	-0,75	1,00	-0,08	-0,55	0,25	-0,43
Górzyca (2)	374	6	26	1,10	-0,60	0,86	0,76	-1,29	0,07	-0,26
Ośno Lubuskie (3)	305	6	19	3,25	-0,86	0,37	0,39	-1,78	1,29	0,15
Rzepin (3)	305	5	22	2,64	-0,58	0,30	0,10	-1,32	1,53	-0,72
Słubice (3)	548	15	38	0,80	-0,01	0,48	-0,11	-0,81	2,84	-0,22
Dobiegniew (3)	500	22	38	0,39	-1,13	0,30	-0,53	-0,63	1,31	-0,34
Drezdenko (3)	1207	14	33	-0,36	-0,62	0,42	-0,21	-0,21	1,15	0,97
Stare Kurowo (2)	695	10	23	-0,46	-0,34	1,19	-0,21	-0,16	-0,27	0,98
Strzelce Krajeńskie (3)	638	13	82	3,76	-0,47	-0,21	-0,23	-1,91	1,15	-0,74
Zwierzyn (2)	660	13	42	-0,04	-0,32	0,76	-0,03	0,23	-0,31	1,19
Krzeszyce (2)	744	23	81	-0,43	-0,67	-0,03	0,04	2,26	0,75	0,88
Lubniewice (3)	203	9	17	-0,21	-0,87	2,60	-0,81	-0,69	1,41	-0,97
Słońsk (2)	391	24	79	1,06	-0,68	0,87	0,64	-0,09	0,45	1,87
Sulęcin (3)	563	2	18	0,30	-0,78	0,15	-0,08	-0,73	0,67	-0,79
Torzym (3)	517	4	11	0,37	-1,28	-0,04	0,53	-0,87	0,62	-0,46
Lubrza (2)	126	7	4	1,10	-0,65	1,09	1,09	-0,75	0,62	-0,22
Łagów (2)	416	5	9	-0,46	-0,97	2,50	-1,13	-0,42	1,69	-0,59
Skąpe (2)	283	7	40	2,07	-0,54	-0,42	-0,43	-0,78	0,96	-0,28
Szczaniec (2)	385	11	20	0,64	-0,70	1,49	0,75	-0,82	-0,45	-1,25

Świebodzin (3)	519	19	45	1,68	0,23	0,07	-0,23	0,48	1,64	-0,80
Zbąszynek (3)	351	15	28	0,06	0,02	0,24	0,13	0,09	0,44	-0,33
Babimost (3)	400	17	49	0,79	0,32	0,59	0,07	0,37	0,11	-0,06
Bojadła (2)	292	5	12	0,49	-0,69	1,02	-0,30	-0,78	-0,15	-0,44
Czerwieńsk (3)	561	6	15	0,28	-0,50	0,47	0,29	-0,90	0,79	-0,47
Kargowa (3)	322	9	17	0,29	-0,36	-0,14	0,02	1,39	1,24	-0,08
Nowogród Bobrzański (3)	799	9	7	-0,89	-0,81	2,14	-0,08	-0,42	0,34	-0,28
Sulechów (3)	727	16	32	0,70	0,29	1,26	-0,24	0,17	0,84	-0,52
Świdnica (2)	862	1	19	-0,56	-0,91	1,47	1,37	-0,76	1,54	-0,15
Trzebiechów (2)	354	3	12	-1,04	-0,89	3,49	1,61	-0,24	-0,28	-1,18
Zabór (2)	408	1	8	-0,85	-0,46	2,34	1,33	-0,49	0,94	-0,28
Brzeźnica (2)	449	7	20	0,34	-0,72	0,95	0,60	-0,75	-0,28	-0,94
Iłowa (3)	464	2	4	-0,74	-0,46	3,23	-0,49	-0,24	0,27	0,28
Małomice (3)	256	8	5	-0,18	-0,40	-0,13	-0,72	-0,23	0,42	-0,39
Niegosławice (2)	441	12	45	0,88	-0,72	-0,34	0,81	-0,66	-0,44	-0,70
Szprotawa (3)	853	21	68	0,81	0,15	-0,03	-0,81	-0,74	0,69	-0,77
Wymiarki (2)	217	0	1	0,00	-0,43	0,72	-0,42	-0,63	0,13	0,67
Żagań (2)	622	22	22	0,68	-0,63	1,20	0,91	-0,57	0,57	-1,05
Brody (2)	419	2	4	0,39	-1,09	0,60	0,55	-1,05	-0,10	-0,40
Jasień (3)	521	6	0	-0,73	-0,36	2,30	-0,22	-0,42	0,53	-0,16
Lipinki Łużyckie (2)	410	5	6	-1,10	-0,94	2,58	1,10	-0,46	-0,09	-0,84
Lubsko (3)	855	5	12	1,50	0,31	1,18	-0,88	-0,57	0,87	0,92
Przewóz (2)	381	1	3	-0,64	-1,06	0,96	0,01	-0,56	0,40	0,14
Trzebiel (2)	835	4	11	-1,06	-1,03	1,29	0,22	-0,46	0,22	-0,31
Tuplice (2)	414	1	2	-0,71	-0,74	0,93	-0,59	-0,34	0,46	-0,07
Żary (2)	1284	19	23	-0,20	-0,67	1,90	0,52	-0,85	0,20	-0,66
Sława (3)	599	20	41	1,04	-0,82	-0,37	0,35	0,60	1,18	-0,05
Szlichtyngowa (3)	299	10	9	0,31	-0,38	-0,20	0,57	-0,38	-0,62	-0,34
Wschowa (3)	927	36	56	0,00	0,05	-0,39	0,03	0,58	0,78	-0,48
Bełchatów (2)	1741	9	37	-1,21	-0,62	-0,37	1,26	-0,29	0,16	-0,01
Drużbice (2)	947	26	68	-0,87	-0,73	-0,25	0,15	0,42	-0,04	-0,05
Kleszczów (2)	502	0	5	-0,94	-0,65	-0,27	3,24	-0,70	0,45	-0,14
Kluki (2)	873	1	9	-0,81	-0,87	-0,72	0,16	-0,66	-0,12	1,13

Rusiec (2)	1051	5	41	-0,85	-0,63	-0,59	-0,48	-0,06	-0,06	0,43
Szczerców (2)	1249	0	17	-1,14	-0,69	-0,24	0,43	-0,33	0,06	0,61
Zelów (3)	1800	10	30	-0,91	-0,50	-0,27	-0,51	-0,28	0,01	0,18
Bedlno (2)	982	71	138	0,26	-0,20	-0,75	-1,71	1,12	-0,67	-0,73
Dąbrowice (2)	325	19	38	0,07	-0,31	-0,64	-1,25	0,46	-0,97	-0,90
Krośniewice (3)	739	41	74	0,36	-0,04	-0,63	-1,14	0,17	-0,62	-1,29
Krzyżanów (2)	607	47	125	-0,07	-0,48	-0,47	0,12	0,58	-0,54	-0,79
Kutno (2)	1089	64	171	0,09	0,07	-0,43	-0,70	-0,03	0,19	-1,61
Łanięta (2)	373	13	46	0,73	-0,06	-0,44	-0,65	-0,13	-1,07	-1,46
Nowe Ostrowy (2)	388	13	26	0,44	-0,12	-0,16	-1,96	-0,05	-0,77	-0,68
Oporów (2)	415	44	90	0,56	0,03	-0,74	-0,90	0,15	-1,11	-1,49
Strzelce (2)	644	36	122	0,30	-0,35	-0,44	-1,18	0,79	-0,18	-1,10
Żychlin (3)	754	29	57	-0,03	0,43	-0,57	-1,57	0,41	-0,38	-1,10
Buczek (2)	1027	12	51	-0,86	-0,73	-0,43	0,02	-0,24	0,06	-0,39
Łask (3)	1446	32	82	-0,75	0,42	-0,32	-0,73	0,29	0,83	0,02
Sędziejowice (2)	1178	23	63	-0,84	-0,77	-0,49	-0,37	-0,38	0,15	-0,50
Widawa (2)	1553	38	94	-0,70	-0,73	-0,54	-1,04	0,35	-0,02	0,25
Wodzierady (2)	550	20	80	-0,41	-0,56	-0,49	0,03	0,67	0,31	-0,03
Daszyna (2)	718	28	103	-0,66	-0,26	0,05	-0,61	-0,06	-0,47	-1,35
Góra Świętej Małgorzaty (2)	1038	90	221	-0,68	-0,32	-0,25	-0,98	-0,09	-0,92	-0,77
Grabów (2)	1295	74	166	-0,15	-0,37	-0,36	-1,16	0,35	-0,83	-0,83
Łęczyca (2)	1466	63	151	-0,61	-0,47	-0,48	-0,14	0,19	-0,75	-0,52
Piątek (2)	1067	77	140	-0,61	-0,54	-0,40	-1,14	0,69	-0,66	-0,20
Świnice Warckie (2)	774	29	126	0,07	0,00	-0,20	-1,79	0,59	-0,55	0,39
Witonia (2)	595	53	143	-0,18	-0,21	-0,67	-0,76	0,37	-0,79	-1,26
Bielawy (2)	1134	76	194	-0,16	-0,37	-0,64	-1,63	1,30	-0,52	-0,17
Chąśno (2)	681	58	110	-0,61	-0,42	-0,59	-0,27	1,95	-0,76	-0,53
Domaniewice (2)	748	27	128	-0,69	-0,68	-0,56	0,21	1,03	-0,30	0,15
Kiernozia (2)	668	35	108	-0,47	-0,26	-0,62	-0,95	0,86	-0,49	-1,00
Kocierzew Południowy (2)	964	82	154	-0,65	-0,40	-0,62	0,21	1,53	-0,87	-0,60
Łowicz (2)	1328	63	169	-0,74	-0,54	-0,41	0,08	0,73	-0,07	0,12
Łyszkowice (2)	880	17	53	-0,66	-0,59	-0,50	-0,20	0,27	-0,49	0,16
Nieborów (2)	1612	9	29	-1,19	-0,40	-0,36	-0,18	-0,23	-0,07	-0,02

Zduny (2)	1061	131	385	-0,06	-0,36	-0,73	-1,37	2,08	-0,51	-0,22
Andrespol (2)	169	2	6	-0,49	4,68	0,43	-0,71	1,50	0,44	-0,73
Brójce (2)	714	12	60	-0,92	-0,50	-0,44	1,32	0,08	0,62	-0,48
Koluszki (3)	1426	11	69	-0,95	0,22	-0,21	-0,65	0,42	0,75	-0,70
Nowosolna (2)	495	4	5	-1,08	0,25	0,04	0,69	-0,08	1,99	-0,72
Rzgów (3)	758	26	74	-1,48	0,12	-0,14	0,24	1,64	3,08	-0,39
Tuszyn (3)	837	26	97	-0,97	0,10	0,41	-0,19	2,74	1,46	-0,65
Białaczów (2)	1108	5	9	-0,81	-0,63	-0,16	-0,64	-0,70	-0,69	-0,04
Drzewica (3)	1699	7	33	-1,05	-0,27	-0,57	-0,68	-0,28	-0,34	0,59
Mniszków (2)	1128	22	59	-1,01	-0,86	-0,31	0,33	-0,13	-0,24	-0,07
Opoczno (3)	2658	13	56	-0,69	0,77	-0,28	-0,13	-0,16	-0,45	0,58
Paradyż (2)	839	22	79	-0,69	-0,42	-0,33	0,39	-0,08	-0,80	0,16
Poświętne (2)	702	18	57	-0,49	-0,93	-0,87	-0,77	-0,05	0,02	1,22
Sławno (2)	1419	23	73	-0,97	-0,14	-0,27	1,03	-0,25	-0,75	0,17
Żarnów (2)	1246	4	52	-1,03	-0,65	0,02	-0,65	-0,11	-0,22	0,41
Dłutów (2)	763	10	52	-1,31	-1,06	-0,51	0,42	0,72	1,24	-0,22
Dobroń (2)	1001	12	30	-1,19	-0,54	-0,26	0,05	0,34	1,05	0,18
Ksawerów (2)	188	2	10	-0,66	4,08	0,25	-1,29	0,62	1,54	-0,10
Lutomiersk (2)	826	20	37	-0,58	-0,49	-0,25	0,31	0,43	0,78	-0,58
Pabianice (2)	846	20	47	-1,12	-0,50	0,09	1,14	0,39	1,35	-0,47
Działoszyn (3)	1378	10	25	-0,72	-0,39	-0,65	-0,19	-0,42	0,79	0,33
Kiełczygłów (2)	916	10	30	-0,66	-0,60	-0,46	-1,61	0,16	0,19	0,61
Nowa Brzeźnica (2)	1048	8	28	-0,90	-0,86	-0,36	-1,14	-0,55	-0,02	-0,09
Pajęczno (3)	1146	12	30	-1,07	-0,66	-0,48	-0,23	-0,14	1,03	-0,36
Rząśnia (2)	1068	5	35	-1,43	-0,52	-0,30	-0,09	0,91	0,39	-0,02
Siemkowice (2)	1012	12	34	-0,88	-0,71	-0,61	-0,24	-0,18	-0,24	0,30
Strzelce Wielkie (2)	898	14	12	-1,15	-0,52	-0,26	-0,53	0,26	-0,47	-0,51
Sulmierzyce (2)	962	15	40	-0,94	-0,58	-0,27	-0,62	-0,17	-0,26	-0,58
Aleksandrów (2)	984	4	11	-0,87	-0,87	-0,20	-0,50	-0,17	-0,37	0,19
Czarnocin (2)	691	27	97	-0,91	-0,62	-0,53	0,49	0,63	0,21	-0,47
Gorzkowice (2)	988	11	30	-0,93	-0,47	0,29	-0,24	-0,19	-0,34	-0,36
Grabica (2)	1196	53	134	-0,79	-0,54	-0,50	0,12	1,36	0,11	-0,66
Łęki Szlacheckie (2)	842	11	29	-0,88	-0,89	-0,38	-0,19	-0,09	-0,63	0,22

Moszczenica (2)	1034	43	125	-0,69	0,19	-0,37	-0,12	0,75	-0,39	-0,84
Ręczno (2)	636	9	35	-1,05	-0,96	-0,47	0,01	0,08	-0,01	0,08
Rozprza (2)	1705	30	53	-0,94	-0,54	-0,24	0,15	-0,21	-0,60	-0,18
Sulejów (3)	1150	13	77	-0,82	-0,49	-0,40	0,14	-0,11	0,56	-0,37
Wola Krzysztoporska (2)	1634	30	112	-0,85	-0,38	-0,16	0,20	0,25	-0,41	-0,57
Dalików (2)	754	40	66	-0,27	-0,61	-0,52	0,14	0,67	-0,08	0,20
Pęczniew (2)	821	34	51	-0,37	-0,72	-0,39	-0,85	0,25	-0,32	0,48
Poddębice (3)	1688	52	124	-0,63	-0,52	-0,34	-0,72	0,35	0,69	0,34
Uniejów (3)	1169	50	168	-0,52	-0,55	-0,37	-1,65	0,63	0,99	0,10
Wartkowice (2)	1144	24	110	-0,44	-0,55	-0,44	-0,56	0,60	-0,57	0,18
Zadzim (2)	990	55	177	-0,20	-0,62	-0,66	-0,65	0,50	-0,21	0,19
Dobryszyce (2)	917	7	17	-1,05	-0,14	0,70	0,13	-0,06	0,13	-0,14
Gidle (2)	1183	1	13	-0,99	-0,66	-0,24	-1,09	-0,30	0,44	0,60
Gomunice (2)	687	4	0	-1,01	-0,17	0,01	-0,69	-0,39	-0,15	0,43
Kamieńsk (3)	800	2	5	-0,78	-0,49	-0,06	-0,64	-0,24	-0,15	0,33
Kobiele Wielkie (2)	781	17	49	-0,41	-0,85	-0,52	-0,13	-0,09	0,19	-0,30
Kodrąb (2)	825	35	63	-0,33	-0,64	-0,56	-0,74	-0,22	-0,14	-0,29
Lgota Wielka (2)	755	12	24	-0,66	-0,22	-0,31	-0,49	-0,14	-0,07	-0,56
Ładzice (2)	879	9	21	-0,86	-0,56	0,10	-0,53	-0,08	-0,01	-0,03
Masłowice (2)	740	33	79	-0,35	-0,68	-0,43	-0,47	0,27	-0,65	-0,07
Przedbórz (3)	961	7	27	-0,94	-1,06	-0,43	-0,30	-0,06	0,55	0,67
Radomsko (2)	759	17	26	-0,64	0,29	0,39	-0,53	-0,37	0,06	0,03
Wielgomłyny (2)	981	15	56	-0,70	-0,76	-0,10	-0,45	0,14	-0,43	0,03
Żytno (2)	1192	12	36	-0,65	-0,82	-0,06	-1,50	-0,15	-0,42	0,41
Biała Rawska (3)	1910	98	572	-1,13	-0,88	-0,40	-0,18	-0,41	0,02	-0,61
Cielądz (2)	923	26	99	-0,77	-0,61	-0,32	-0,25	-0,20	-0,58	0,07
Rawa Mazowiecka (2)	1638	25	156	-1,01	-0,69	-0,32	0,13	0,07	0,23	-0,30
Regnów (2)	445	24	69	-0,58	-0,55	-0,46	-0,38	-0,21	-0,65	0,14
Sadkowice (2)	1332	58	390	-1,06	-0,46	-0,38	-0,49	-0,37	-0,99	-0,60
Błaszki (3)	2312	96	268	-0,58	-0,15	-0,82	-0,40	-0,24	-0,62	-0,37
Brąszewice (2)	753	30	60	-0,60	-0,69	-0,53	1,17	0,36	-0,36	0,63
Brzeźnio (2)	1101	56	115	-0,34	-0,61	-0,80	-0,21	0,08	-0,02	-0,05
Burzenin (2)	798	28	55	-0,44	-0,76	-0,69	-0,88	0,26	0,24	0,32

Goszczanów (2)	1095	57	314	-0,07	-0,24	-0,66	-0,61	1,31	-0,73	0,00
Klonowa (2)	567	30	52	-0,27	-0,74	-0,49	-0,20	0,66	-0,61	0,41
Sieradz (2)	1544	57	120	-0,65	-0,69	-0,49	0,49	-0,48	0,13	0,42
Warta (3)	1995	98	198	-0,23	-0,25	-0,89	-0,79	-0,27	-0,17	0,16
Wróblew (2)	1230	36	195	-0,22	-0,23	-1,42	0,09	-0,66	-0,64	0,36
Złoczew (3)	1021	20	67	-0,34	-0,46	-0,63	-0,12	0,41	-0,32	0,19
Bolimów (2)	771	11	33	-0,77	-0,86	-0,40	-0,06	-0,07	0,09	-0,33
Głuchów (2)	1169	51	256	-0,58	-0,47	-0,59	-0,34	0,43	-0,31	-0,13
Godzianów (2)	452	14	61	-0,56	-0,27	-0,25	0,52	0,66	-0,36	-0,34
Kowiesy (2)	800	23	118	-1,00	-0,67	-0,12	-1,41	-0,50	0,02	-0,42
Lipce Reymontowskie (2)	607	6	15	-1,18	-0,07	-0,22	-0,37	0,37	-0,34	-0,48
Maków (2)	1078	4	12	-1,17	-0,55	-0,36	0,12	-0,27	-0,37	-0,02
Nowy Kawęczyn (2)	845	19	102	-0,68	-0,69	-0,21	-0,62	-0,15	-0,27	-0,46
Skierniewice (2)	1415	18	62	-1,09	-0,71	0,00	0,48	-0,28	0,49	-0,53
Słupia (2)	566	17	17	-0,50	0,40	-0,27	-1,13	0,27	-0,64	-0,26
Będków (2)	580	28	51	-0,46	-0,24	-0,64	-0,92	0,95	-0,60	-0,32
Budziszewice (2)	435	7	39	-1,11	-0,59	-0,43	0,75	0,10	0,39	-0,53
Czerniewice (2)	1111	42	99	-1,08	-0,91	-0,34	0,56	0,16	-0,11	-0,08
Inowłódz (2)	574	4	1	-1,17	-0,99	-0,59	-1,55	-0,24	1,25	0,65
Lubochnia (2)	1233	12	31	-1,50	-0,91	-0,17	0,73	-0,30	0,05	-0,58
Rokiciny (2)	752	25	118	-0,65	-0,40	-0,47	-0,67	0,70	0,11	-0,58
Rzeczyca (2)	1019	36	79	-1,00	-0,77	-0,49	-0,08	0,32	-0,57	0,07
Tomaszów Mazowiecki (2)	2207	17	33	-1,44	-0,66	-0,21	0,81	-0,04	0,77	-0,28
Ujazd (2)	858	34	67	-0,51	-0,18	-0,27	-0,82	0,06	0,47	-0,25
Żelechlinek (2)	721	21	115	-0,81	-0,86	-0,28	-0,38	-0,05	-0,33	-0,25
Biała (2)	881	100	294	-0,32	-0,29	-0,96	-0,21	-0,44	-0,19	-0,18
Czarnożyły (2)	792	17	53	-0,16	0,20	-1,19	-0,27	-0,68	-0,37	0,21
Konopnica (2)	707	22	50	-0,67	-0,64	-0,53	-0,47	0,09	0,09	-0,22
Mokrsko (2)	891	17	22	-0,69	-0,11	-0,45	0,01	-0,08	-0,51	-0,70
Osjaków (2)	1010	20	33	-0,86	-0,79	-0,69	0,14	-0,19	0,56	0,52
Ostrówek (2)	845	7	14	-0,69	-0,68	-0,64	0,00	0,01	-0,24	0,60
Pątnów (2)	1238	15	20	-0,85	-0,72	-0,76	-0,15	-0,48	0,60	0,10
Skomlin (2)	528	13	35	-0,37	-0,55	-0,51	-0,03	-0,30	-0,40	-0,34

Wieluń (3)	1742	27	39	-0,37	1,28	-0,45	-1,07	0,02	0,76	-0,19
Wierzchlas (2)	1202	14	41	-1,00	-0,57	-0,28	0,16	-0,31	0,05	-0,16
Bolesławiec (2)	724	18	17	-0,68	-0,29	-0,53	-0,26	0,02	0,34	-0,44
Czastary (2)	640	6	8	-0,83	-0,54	-0,52	0,16	-0,19	0,15	-0,13
Galewice (2)	1026	4	30	-0,94	-0,81	-0,50	0,66	-0,21	0,08	0,28
Lututów (2)	748	22	49	-0,64	-0,46	-0,20	0,23	0,58	0,00	-0,02
Łubnice (2)	721	27	44	-0,25	-0,23	-0,68	-0,22	-0,20	0,05	-1,00
Sokolniki (2)	774	11	29	-1,13	-0,53	-0,67	0,57	0,02	0,04	-0,41
Wieruszów (3)	1013	11	27	-0,63	0,00	-0,43	-0,17	0,02	1,29	0,11
Szadek (3)	1056	64	159	-0,41	-0,55	-0,44	-0,57	0,24	0,29	-0,62
Zapolice (2)	846	8	40	-0,89	-0,57	0,07	0,26	-0,23	0,22	-0,29
Zduńska Wola (2)	1575	30	45	-1,04	-0,13	0,01	0,40	-0,23	0,32	-0,08
Aleksandrów Łódzki (3)	640	14	10	-0,29	1,32	-0,06	-0,33	0,63	1,58	-0,09
Głowno (2)	938	38	120	-0,59	-0,40	-0,35	-0,46	0,52	-0,61	-0,08
Ozorków (2)	775	30	36	-0,50	0,03	-0,52	-0,35	0,50	0,26	-0,17
Parzęczew (2)	621	11	54	-0,50	-0,75	-0,35	0,79	0,22	0,33	-0,25
Stryków (3)	1414	7	65	-0,89	-0,36	-0,15	-0,66	0,22	0,95	-0,35
Zgierz (2)	1234	23	82	-1,37	-0,49	-0,15	-0,07	1,09	1,64	-0,33
Brzeziny (2)	985	9	43	-1,08	-0,83	-0,16	0,34	-0,05	0,72	-0,73
Dmosin (2)	1053	29	103	-1,00	-0,71	-0,31	-0,46	0,35	0,39	-0,68
Jeżów (2)	578	19	35	-0,42	-0,61	-0,48	-0,96	0,19	0,25	-0,70
Rogów (2)	621	14	38	0,06	-0,66	-1,50	0,04	-0,53	0,34	0,75
Bochnia (2)	3544	7	15	-0,61	2,21	-0,10	0,35	-0,56	-0,65	0,26
Drwinia (2)	1341	4	7	-0,90	0,30	-0,14	-0,34	-0,86	-0,50	-0,36
Lipnica Murowana (2)	1048	2	13	-0,35	0,20	-0,39	1,52	-1,09	-0,46	1,57
Łapanów (2)	1337	3	20	-0,86	1,28	-0,25	1,64	-0,19	-0,43	0,48
Nowy Wiśnicz (3)	2387	0	2	-0,60	1,39	-0,50	1,36	-0,86	-0,58	1,02
Rzezawa (2)	2071	2	14	-0,72	1,22	-0,08	1,02	-0,73	-0,52	0,29
Trzciana (2)	1027	2	60	-0,66	1,81	0,22	1,54	-0,13	-0,69	0,62
Żegocina (2)	907	2	5	-0,25	2,05	-0,23	1,06	-0,67	-0,79	1,40
Borzęcin (2)	1293	1	29	-0,81	0,29	-0,14	0,10	-0,52	-0,55	-0,03
Brzesko (3)	3160	3	7	-0,32	3,01	0,22	-0,31	-0,29	-0,50	0,97
Czchów (3)	1328	3	13	-0,57	2,35	-0,35	0,68	-0,32	-0,78	0,67

Dębno (2)	2377	19	79	-0,27	2,39	-0,01	0,15	-0,89	-0,95	0,64
Gnojnik (2)	1551	1	7	-0,55	1,34	-0,09	0,60	-0,97	-0,87	0,81
Iwkowa (2)	1286	1	25	-0,56	1,12	-0,59	1,09	-0,62	-0,21	1,49
Szczurowa (2)	1538	14	11	-0,65	0,17	-0,12	-0,49	-0,47	-0,51	0,40
Alwernia (3)	1125	2	0	-0,82	1,57	0,17	-0,70	-0,28	0,16	-0,07
Babice (2)	469	1	4	-0,52	1,44	-0,31	-0,90	-0,54	0,34	0,34
Chrzanów (3)	652	31	93	0,51	4,94	0,01	-2,52	0,07	0,06	1,57
Libiąż (3)	290	0	0	0,00	3,08	0,21	-1,14	0,16	0,03	1,35
Trzebinia (3)	831	0	0	0,01	2,83	0,17	-1,30	-0,23	0,79	1,21
Bolesław (2)	450	6	20	-0,74	0,98	-0,46	-0,77	-1,14	-1,33	0,23
Dąbrowa Tarnowska (3)	2458	11	19	-0,68	1,54	0,06	0,05	-0,45	-0,38	0,30
Gręboszów (2)	600	18	31	-0,53	0,81	-0,02	-2,23	-0,20	-1,08	-1,54
Mędrzechów (2)	471	8	14	-0,79	0,75	-0,09	-0,68	-0,18	-0,88	-0,55
Olesno (2)	1351	40	129	-0,77	0,59	-0,20	0,24	-0,42	-0,83	-0,74
Radgoszcz (2)	1303	19	23	-1,08	0,67	1,64	0,41	-0,33	-1,32	-0,08
Szczucin (3)	2105	10	47	-1,24	0,50	0,07	0,06	-0,14	-0,64	-0,62
Biecz (3)	3116	8	11	-0,38	1,73	0,00	-0,48	-0,85	-0,99	0,98
Bobowa (3)	1435	3	4	-0,25	2,22	-0,24	1,24	-0,71	-1,44	0,95
Gorlice (2)	2931	4	18	-0,05	2,96	0,08	-0,27	-0,70	-0,70	1,71
Lipinki (2)	1443	2	19	-0,37	1,11	0,60	-0,45	-1,05	-0,75	1,28
Łużna (2)	1573	2	20	-0,57	1,01	-0,11	0,76	-0,79	-1,06	1,07
Moszczenica (2)	823	3	3	-0,27	1,48	0,08	0,70	-0,81	-1,20	1,33
Ropa (2)	1028	2	6	-0,78	0,18	-0,22	1,49	-0,73	0,41	1,30
Sękowa (2)	1013	4	24	-0,44	-0,47	1,82	-0,16	-0,60	0,16	1,76
Uście Gorlickie (2)	1176	20	111	-0,32	-0,67	3,30	1,28	-0,34	0,33	2,09
Czernichów (2)	2150	3	23	-1,08	0,99	-0,16	0,64	-0,11	0,34	-0,76
Igołomia-Wawrzeńczyce (2)	1511	24	193	-1,08	0,76	-0,40	-0,14	-0,61	-0,30	-1,57
Iwanowice (2)	1866	16	33	-1,09	1,40	-0,21	0,33	-0,41	-0,23	-1,13
Jerzmanowice-Przeginia (2)	1992	6	9	-1,16	1,38	0,29	-0,16	-0,31	0,12	-0,28
Kocmyrzów-Luborzyca (2)	2073	26	81	-0,94	1,99	-0,44	0,71	-0,59	-0,30	-1,27
Krzeszowice (3)	2780	3	11	-0,87	2,28	0,99	-1,16	-0,20	0,32	-0,33
Liszki (2)	2023	7	13	-1,09	2,65	0,08	0,77	-0,12	0,09	-0,89
Michałowice (2)	1153	4	33	-1,06	1,87	0,17	1,10	-0,53	0,77	-1,06

Mogilany (2)	820	1	0	-0,74	3,29	0,01	0,72	-0,05	0,78	0,05
Skała (3)	1782	2	18	-1,11	1,03	0,01	0,11	-0,50	0,27	-0,86
Skawina (3)	1635	6	7	-0,29	4,17	0,79	-0,55	0,16	-0,04	0,21
Słomniki (3)	1938	35	102	-1,01	0,40	-0,40	-0,10	-0,28	0,14	-1,09
Sułoszowa (2)	1229	30	43	-0,72	1,25	-0,31	-0,20	-0,45	-1,08	-0,33
Świątniki Górne (3)	624	2	4	0,08	5,19	-0,38	0,44	-0,08	-0,28	0,16
Wielka Wieś (2)	1544	1	5	-0,85	3,05	0,10	1,08	-0,37	0,95	-0,63
Zabierzów (2)	1146	4	7	-0,25	3,11	0,61	-0,33	-0,19	1,09	-1,49
Zielonki (2)	1780	7	31	-0,57	4,91	0,18	0,96	0,14	0,84	-1,06
Dobra (2)	1967	4	16	-0,49	0,47	0,40	1,10	-0,74	-0,48	2,34
Jodłownik (2)	1565	12	168	-0,86	1,14	2,00	1,04	-0,40	-0,93	0,48
Kamienica (2)	1028	0	0	0,14	-0,18	-0,94	1,30	-1,39	-0,20	3,52
Laskowa (2)	1241	1	45	-0,35	0,49	-0,14	2,10	-0,86	-0,54	2,38
Limanowa (2)	3796	2	24	-0,45	1,15	-0,36	1,61	-0,65	-0,63	2,26
Łukowica (2)	1529	0	27	-0,60	0,24	-0,28	1,81	-0,79	-0,96	1,94
Mszana Dolna (2)	3223	0	10	-0,27	0,25	-0,51	1,62	-0,85	-0,01	2,70
Niedźwiedź (2)	938	3	9	-0,25	0,77	1,00	1,95	-0,88	-0,73	2,79
Słopnice (2)	888	0	18	-0,58	0,59	1,20	2,91	-0,71	-0,72	2,12
Tymbark (2)	896	2	3	-0,49	1,62	0,27	1,16	-0,47	-0,42	2,22
Charsznica (2)	1645	44	287	-1,00	0,51	-0,52	-1,31	-0,43	-0,32	-1,08
Gołcza (2)	1368	58	169	-1,13	-0,31	-0,50	0,07	-0,33	-0,15	-1,13
Kozłów (2)	938	39	70	-1,02	-0,46	-0,24	-0,96	-0,02	-0,29	-0,56
Książ Wielki (2)	971	73	129	-0,77	-0,65	-0,25	-1,00	0,22	0,10	-0,97
Miechów (3)	2323	80	252	-1,10	0,25	-0,50	-1,02	0,03	1,32	-1,02
Racławice (2)	537	50	113	-0,87	-0,57	-0,82	-0,08	0,15	0,14	-1,17
Słaboszów (2)	820	65	198	-0,69	-0,42	-0,56	-0,96	0,29	-0,57	-0,80
Dobczyce (3)	940	0	7	-0,72	1,77	-0,14	1,04	-0,32	0,04	0,75
Lubień (2)	1639	0	0	-0,22	0,53	-0,80	0,91	-1,20	-0,20	2,27
Myślenice (3)	5092	0	19	-0,42	3,05	-0,42	0,47	-0,21	0,55	1,31
Pcim (2)	1820	0	0	0,11	0,18	-1,40	0,97	-1,44	-0,11	3,15
Raciechowice (2)	1402	8	71	-1,20	0,08	-0,43	1,29	-0,28	-0,34	0,60
Siepraw (2)	1538	0	0	-0,77	3,08	0,08	1,32	-0,19	0,16	0,36
Sułkowice (3)	2169	1	1	-0,45	1,88	-0,67	1,11	-0,80	-0,24	0,84

Tokarnia (2)	1463	1	0	-0,43	-0,06	-1,06	2,21	-1,27	-0,25	2,28
Wiśniowa (2)	1220	2	7	-0,71	0,25	-0,27	1,27	-0,52	0,14	1,89
Chełmiec (2)	2308	2	20	-0,65	2,66	-0,26	1,81	-0,16	-0,87	1,08
Gródek nad Dunajcem (2)	1153	5	7	-0,34	1,45	0,71	0,35	-0,73	-0,98	1,27
Grybów (2)	3649	7	26	-0,43	1,19	-0,29	1,68	-0,74	-0,77	1,69
Kamionka Wielka (2)	1215	1	2	-0,18	1,77	-0,67	1,89	-0,83	-0,77	2,30
Korzenna (2)	2312	13	22	-0,50	1,27	-0,08	1,67	-0,71	-1,03	0,99
Krynica-Zdrój (3)	935	13	28	1,77	0,56	-0,59	-0,90	-1,19	2,52	5,37
Łabowa (2)	920	3	9	-0,31	-0,52	0,53	2,45	-1,12	-0,34	2,58
Łącko (2)	2030	6	95	-0,66	-0,20	-0,18	2,28	-1,05	-0,44	1,78
Łososina Dolna (2)	1358	9	70	-0,68	0,98	0,30	1,79	-0,73	-0,72	1,02
Muszyna (3)	1002	0	5	0,20	0,08	-0,61	-0,05	-1,23	1,06	3,12
Nawojowa (2)	1265	0	5	-0,75	0,38	0,60	2,45	-0,67	-0,65	1,69
Piwniczna-Zdrój (3)	1292	0	0	-0,45	-0,73	-0,80	0,98	-1,04	0,74	2,89
Podegrodzie (2)	1639	1	39	-0,99	0,86	-0,11	2,13	-0,62	-1,07	0,40
Rytro (2)	456	0	0	0,31	-0,32	0,13	0,00	-1,70	0,42	2,36
Stary Sącz (3)	1938	1	27	-0,48	1,70	-0,02	0,85	-0,56	-0,42	1,20
Szczawnica (3)	1076	0	2	-0,56	-0,80	-0,15	-0,68	-0,96	2,65	2,81
Czarny Dunajec (2)	2702	0	31	-0,40	0,01	0,04	0,48	-0,55	-0,71	2,59
Czorsztyn (2)	1255	0	0	-0,57	-0,11	-0,65	0,72	-0,83	0,76	2,05
Jabłonka (2)	3545	0	10	-0,28	0,15	-0,63	1,77	-1,03	-0,67	2,83
Krościenko nad Dunajcem (2)	1271	0	5	-0,51	-0,12	-0,91	0,62	-0,75	0,73	2,82
Lipnica Wielka (2)	937	0	0	-0,25	-0,19	-0,89	1,69	-1,44	-0,72	2,62
Łapsze Niżne (2)	1578	1	19	-0,42	-0,50	-0,46	0,87	-0,89	-0,13	2,76
Nowy Targ (2)	3007	3	20	-0,21	0,19	-0,53	0,93	-0,66	-0,33	2,86
Ochotnica Dolna (2)	1459	0	0	0,18	-0,58	-1,33	1,64	-1,52	-0,11	3,81
Raba Wyżna (2)	2506	0	9	2,23	1,35	-0,79	0,56	-2,46	-0,70	4,76
Rabka-Zdrój (3)	1193	0	0	0,08	2,35	-0,40	-1,10	-0,47	0,22	3,08
Spytkowice (2)	632	3	14	-0,92	0,26	-0,21	1,32	-0,79	-0,38	-0,97
Szaflary (2)	1569	0	14	-0,29	1,57	-0,05	0,61	-0,20	-1,06	2,74
Bolesław (2)	1184	0	0	0,14	2,30	-0,15	-2,41	0,23	0,42	-0,10
Klucze (2)	2568	1	1	-0,93	0,17	-0,56	-0,91	-0,65	1,14	0,87
Olkusz (3)	2610	1	21	-0,74	1,92	-0,07	-1,11	0,03	1,01	0,64

Trzyciąż (2)	1425	17	77	-1,01	0,13	-0,59	-0,18	-0,41	-0,33	-0,40
Wolbrom (3)	2822	26	114	-1,00	1,06	0,06	-1,07	-0,05	0,54	-0,63
Brzeszcze (3)	700	1	3	-0,04	3,60	-0,01	-0,92	0,19	0,01	-0,50
Chełmek (3)	650	0	0	0,15	4,72	0,00	-1,83	0,02	-0,72	1,38
Kęty (3)	734	2	29	0,33	4,29	0,12	-1,00	0,12	-0,18	-0,79
Osiek (2)	1204	2	3	-0,42	1,66	-0,22	0,12	-0,34	-0,88	-1,34
Oświęcim (2)	901	3	18	-0,72	1,75	-0,04	-0,33	-0,17	0,11	-0,50
Polanka Wielka (2)	435	2	30	-0,42	1,14	-0,26	0,61	-0,15	-0,43	-1,28
Przeciszów (2)	951	4	24	-0,10	2,14	-0,36	-0,45	-0,45	-0,74	-0,92
Zator (3)	557	2	17	0,12	2,67	-0,08	-0,51	-0,27	-0,34	-0,51
Koniusza (2)	1557	48	220	-1,12	1,11	-0,45	0,30	-0,35	-0,74	-1,70
Koszyce (2)	996	18	57	-0,81	0,18	-0,44	-0,89	-0,40	-0,31	-1,38
Pałecznica (2)	765	50	193	-0,83	-0,15	-0,72	-0,20	-0,43	-0,30	-1,11
Proszowice (3)	1830	53	234	-0,80	1,00	-0,58	-1,08	-0,24	0,11	-1,49
Radziemice (2)	714	54	134	-1,01	-0,16	-0,68	-0,10	-0,25	-0,17	-1,32
Budzów (2)	1534	2	1	-0,47	-0,54	-1,09	1,51	-1,20	0,06	2,15
Bystra-Sidzina (2)	1383	1	4	-0,64	-0,77	-0,96	1,09	-1,11	0,38	1,96
Jordanów (2)	1726	1	10	-0,52	0,27	-0,59	0,59	-0,77	-0,01	2,10
Maków Podhalański (3)	2488	0	0	-0,30	0,28	-1,12	-0,28	-1,05	1,13	2,21
Stryszawa (2)	2455	1	3	-0,55	-0,07	-0,96	0,29	-1,07	-0,04	2,36
Zawoja (2)	2639	0	2	-0,70	-0,71	-0,95	0,44	-1,10	0,88	2,38
Zembrzyce (2)	650	0	1	-0,28	1,11	-0,76	-0,40	-0,72	0,37	1,94
Ciężkowice (3)	1592	7	26	-0,46	1,01	-0,37	0,14	-0,66	-0,72	0,67
Gromnik (2)	1505	0	2	-0,59	1,04	-0,60	0,98	-0,87	-0,85	0,80
Lisia Góra (2)	1827	9	16	-0,64	1,37	-0,27	1,05	-0,64	-0,69	0,41
Pleśna (2)	1865	4	2	-0,50	1,33	-0,21	0,17	-0,97	-0,94	0,63
Radłów (3)	1475	39	77	-0,26	1,20	-0,13	-0,27	-0,78	-0,80	-0,32
Ryglice (3)	2058	2	6	-0,53	0,64	-0,03	0,17	-0,99	-0,85	0,75
Rzepiennik Strzyżewski (2)	1412	2	7	-0,46	0,82	-0,34	0,14	-0,87	-0,94	0,94
Skrzyszów (2)	1656	2	6	-0,66	1,62	0,17	0,61	-0,52	-0,59	-0,03
Tarnów (2)	1480	6	17	-0,24	3,80	0,03	-0,05	-0,47	-0,65	0,31
Tuchów (3)	1867	5	21	-0,39	2,01	-0,21	-0,04	-0,70	-0,90	0,58
Wierzchosławice (2)	1476	1	0	-0,53	1,49	-0,26	-0,08	-1,13	-0,54	-0,70

Wietrzychowice (2)	534	6	19	-0,37	1,18	-0,02	-1,68	-0,42	-0,94	-0,38
Wojnicz (3)	1966	2	6	-0,60	1,77	-0,22	0,19	-0,80	-0,74	0,15
Zakliczyn (3)	2132	7	23	-0,68	0,38	-0,36	0,83	-0,77	-0,39	0,60
Żabno (3)	2774	17	56	-0,28	2,25	-0,29	-0,36	-0,63	-0,98	-1,05
Biały Dunajec (2)	1325	0	1	-0,40	1,45	-0,34	0,09	-0,34	0,01	2,61
Bukowina Tatrzańska (2)	3145	0	7	-1,03	-0,71	-0,56	0,86	-0,28	1,63	2,28
Kościelisko (2)	900	0	0	-0,66	-0,75	-0,77	0,32	-0,58	1,95	2,86
Poronin (2)	1890	0	10	-0,89	-0,01	-0,07	0,84	-0,01	1,92	2,46
Andrychów (3)	2768	2	20	0,12	3,74	-0,28	-0,53	-0,12	-0,07	1,17
Brzeźnica (2)	1905	5	5	-0,87	1,49	-0,19	0,34	-0,51	-0,24	-0,26
Kalwaria Zebrzydowska (3)	3168	1	7	-0,42	3,02	-0,40	-0,27	-0,25	0,75	0,89
Lanckorona (2)	1339	0	0	-0,66	1,61	-0,43	0,46	-0,58	0,03	0,80
Mucharz (2)	535	0	0	-0,67	0,99	-0,28	0,79	-0,44	0,02	0,81
Spytkowice (2)	1248	0	0	0,30	3,73	-0,56	0,05	-0,59	-1,15	2,63
Stryszów (2)	1008	3	0	-0,58	1,34	-0,23	0,23	-0,88	0,08	0,78
Tomice (2)	972	3	16	-0,81	1,60	-0,18	1,14	-0,10	0,16	-0,72
Wadowice (3)	3200	0	8	-0,05	3,71	-0,39	-0,96	-0,30	0,16	1,25
Wieprz (2)	1753	11	72	-0,65	1,61	-0,21	0,91	-0,14	-0,72	-0,55
Biskupice (2)	1457	0	3	-1,28	1,87	-0,08	1,11	-0,41	-0,29	-0,57
Gdów (2)	2320	12	50	-1,22	1,53	0,03	0,99	0,06	-0,21	-1,05
Kłaj (2)	1175	1	4	-0,83	1,63	0,04	0,14	-0,66	0,35	-0,65
Niepołomice (3)	3425	2	22	-0,76	3,02	0,33	1,08	-0,12	0,51	-0,20
Wieliczka (3)	2849	1	15	-0,63	5,19	0,21	0,11	0,60	0,33	-0,37
Białobrzegi (3)	752	12	54	-0,59	0,47	0,94	-0,32	-0,17	1,43	0,62
Promna (2)	1497	49	179	-0,84	-0,52	0,10	0,23	-0,59	0,05	0,05
Radzanów (2)	886	25	50	-0,42	-0,29	-0,33	0,36	5,96	-0,40	1,60
Stara Błotnica (2)	1129	35	50	-1,06	-0,63	-0,29	1,91	0,03	-0,22	0,05
Stromiec (2)	1119	7	27	-0,96	-0,92	-0,06	0,74	-0,32	-0,17	0,54
Wyśmierzyce (3)	615	11	28	-0,78	-1,03	-0,19	0,07	-0,58	0,09	0,55
Ciechanów (2)	900	104	144	-0,04	-0,50	-0,27	1,15	0,30	-0,13	-0,34
Glinojeck (3)	780	27	29	-0,53	-0,31	-0,25	-0,05	1,01	-0,11	0,68
Gołymin-Ośrodek (2)	532	72	108	1,15	-0,44	-0,64	0,61	0,69	-1,10	-0,58
Grudusk (2)	450	75	139	1,64	-0,18	-0,81	-0,89	1,12	-0,74	-0,59

Ojrzeń (2)	747	40	62	-0,14	-0,68	-0,56	0,69	0,30	-0,56	0,59
Opinogóra Górna (2)	781	98	201	0,77	-0,34	-0,60	0,95	1,06	-0,82	-0,97
Regimin (2)	571	33	44	0,39	-0,32	-0,47	0,08	0,50	-0,85	-0,03
Sońsk (2)	926	75	120	0,48	-0,08	-0,43	-0,33	0,86	-0,76	0,03
Borowie (2)	812	15	34	-0,76	-0,17	-0,42	0,93	0,13	-0,86	0,31
Garwolin (2)	1161	5	25	-0,79	0,05	-0,14	1,44	-0,32	-0,28	0,02
Górzno (2)	948	26	49	-1,04	-0,47	-0,63	1,75	-0,14	-0,38	0,18
Łaskarzew (2)	778	7	28	-0,35	-0,22	-0,63	1,02	-0,31	-0,66	0,78
Maciejowice (2)	1010	14	80	-0,48	-0,61	-0,09	-0,59	-0,23	-0,37	0,65
Miastków Kościelny (2)	823	22	17	-0,68	-0,25	-0,34	0,52	0,14	-0,99	0,16
Parysów (2)	695	5	12	-0,74	-0,46	-0,35	0,87	-0,11	-0,90	0,79
Pilawa (3)	757	1	5	-0,66	0,43	-0,35	0,92	-0,45	-0,30	0,76
Sobolew (2)	816	16	47	-0,66	0,04	-0,66	1,08	0,25	-0,56	0,42
Trojanów (2)	1214	27	72	-0,57	-0,40	-0,66	0,10	0,17	-0,88	0,33
Wilga (2)	908	15	114	-1,11	-0,79	-0,71	0,01	-0,56	0,16	-0,03
Żelechów (3)	929	14	33	-0,73	0,14	-0,56	1,10	0,18	-0,56	0,09
Gostynin (2)	1485	45	113	-0,71	-0,45	-0,18	-0,27	1,34	0,36	-0,34
Pacyna (2)	568	29	29	0,28	-0,26	-0,53	-1,49	0,92	-0,67	-0,70
Sanniki (2)	963	30	69	-0,35	-0,32	-0,24	-0,64	0,10	-0,42	-0,97
Szczawin Kościelny (2)	791	27	63	-0,13	-0,61	-0,42	-0,51	0,00	-0,62	-0,47
Baranów (2)	692	8	24	-0,26	0,11	-0,61	0,71	-0,09	0,00	-0,52
Grodzisk Mazowiecki (3)	477	8	22	0,17	3,20	0,44	0,23	0,56	1,29	-0,37
Jaktorów (2)	550	1	5	-0,44	1,74	-0,20	0,91	-0,07	0,76	0,55
Żabia Wola (2)	790	3	7	-1,50	-0,32	-0,10	1,93	0,03	1,60	-0,18
Belsk Duży (2)	1351	46	305	-0,97	0,28	-0,12	-1,17	-0,64	0,00	-1,39
Błędów (2)	1703	48	330	-0,63	-0,29	-0,34	-0,61	-1,02	0,10	-1,08
Chynów (2)	1156	32	209	-1,08	-0,03	-0,34	0,44	-0,29	-0,03	-0,39
Goszczyn (2)	654	34	74	-0,99	-0,03	0,01	0,68	-0,72	-0,58	-0,62
Grójec (3)	1332	20	176	-1,40	1,37	0,04	0,01	0,40	1,53	-0,85
Jasieniec (2)	1059	21	104	-1,01	-0,58	-0,15	0,10	-0,59	-0,03	-0,47
Mogielnica (3)	1315	47	161	-0,80	-0,21	-0,13	-1,02	-0,58	-0,14	-0,25
Nowe Miasto nad Pilicą (3)	1073	38	70	-0,81	-0,70	-0,62	-1,52	-0,04	0,30	0,20
Pniewy (2)	879	41	247	-1,11	-0,56	-0,44	0,93	-0,61	0,44	-0,27

Warka (3)	1921	102	568	-1,14	-0,58	-0,29	0,26	-0,51	0,37	-0,37
Garbatka-Letnisko (2)	363	5	0	-1,09	-0,16	0,11	-0,79	0,33	0,44	0,38
Głowaczów (2)	1382	16	21	-0,87	-0,78	0,33	-0,16	-0,26	-0,49	0,51
Gniewoszów (2)	698	24	36	-0,70	-0,60	-0,38	-0,57	0,06	-0,25	-0,10
Grabów nad Pilicą (2)	738	15	37	-0,65	-0,83	-0,36	0,08	-0,17	-0,53	0,85
Kozienice (3)	1453	23	87	-0,44	0,46	-0,40	-1,00	-0,41	0,51	0,16
Magnuszew (2)	1180	28	86	-1,05	-0,82	-0,13	0,46	-0,50	-0,57	0,18
Sieciechów (2)	510	18	24	-0,23	0,36	-0,20	-1,35	-0,29	-1,23	-0,31
Jabłonna (2)	352	2	7	-0,76	0,58	1,08	1,87	-0,71	2,40	0,28
Nieporęt (2)	377	4	5	-0,54	0,61	0,02	0,96	-0,28	2,93	0,83
Serock (3)	636	6	26	-0,86	0,18	-0,10	1,01	-0,04	1,72	-0,01
Wieliszew (2)	346	3	5	-0,71	0,20	0,92	1,79	-0,25	2,58	0,45
Chotcza (2)	609	10	19	-0,69	-0,51	0,10	-2,02	-0,16	-0,65	0,38
Ciepielów (2)	1173	28	53	-0,90	-0,84	-0,20	-0,11	0,22	-0,16	0,04
Lipsko (3)	1677	59	60	-1,12	-0,60	-0,36	-1,16	-0,25	1,09	-0,50
Rzeczniów (2)	1106	34	31	-1,00	-0,75	-0,23	-0,80	-0,24	-0,08	-0,51
Sienno (2)	1438	65	61	-0,99	-0,79	-0,02	-0,61	-0,26	-0,05	-0,75
Solec nad Wisłą (2)	1245	40	37	-0,80	-0,53	-0,58	-2,02	-0,67	-0,31	-0,22
Huszlew (2)	742	44	83	-0,26	-0,74	-0,48	-0,82	0,27	-0,52	0,17
Łosice (3)	1153	93	115	-0,65	-0,04	-0,22	-0,33	0,58	0,70	-0,21
Olszanka (2)	672	68	144	0,17	-0,07	-0,35	-0,71	1,20	-0,42	0,55
Platerów (2)	1008	79	49	-0,53	-0,37	-0,15	-0,23	0,09	-0,63	-0,03
Sarnaki (2)	924	38	72	-0,33	-0,90	-0,39	-1,32	0,06	0,43	0,68
Stara Kornica (2)	1048	92	93	-0,36	-0,55	-0,47	-0,43	0,33	-0,75	0,03
Czerwonka (2)	480	39	85	0,28	-0,78	-0,58	0,01	0,35	-0,40	1,17
Karniewo (2)	849	87	166	0,46	-0,39	-0,47	0,09	1,07	-0,83	-0,36
Krasnosielc (2)	914	78	151	0,31	-0,64	-0,40	0,16	1,16	-0,17	1,36
Młynarze (2)	371	38	66	0,19	-0,45	2,95	0,96	0,87	-0,81	1,22
Płoniawy-Bramura (2)	903	50	145	0,11	-0,39	-0,38	-0,46	0,62	-0,28	0,47
Różan (3)	494	17	49	-0,46	-0,26	2,72	-0,66	0,27	0,23	0,45
Rzewnie (2)	686	35	115	-0,15	-0,80	2,13	0,05	0,49	-0,58	0,98
Sypniewo (2)	615	57	148	0,83	-0,44	-0,15	0,05	1,57	-0,82	1,35
Szelków (2)	630	25	93	-0,14	-0,70	-0,57	0,44	0,75	0,09	-0,01

Cegłów (2)	1013	19	26	-1,39	-0,56	-0,34	-0,32	0,55	0,18	-0,02
Dębe Wielkie (2)	673	1	4	-1,09	-0,37	-0,21	1,58	-0,39	0,68	0,56
Dobre (2)	940	9	17	-0,93	-0,80	0,24	0,22	-0,18	-0,09	0,31
Halinów (3)	560	0	8	-0,87	1,52	0,05	1,26	-0,09	0,80	0,46
Jakubów (2)	917	13	38	-1,01	-0,54	-0,30	0,38	0,05	-0,06	0,18
Kałuszyn (3)	759	16	24	-0,90	-0,73	0,00	0,15	0,01	0,14	0,52
Latowicz (2)	967	35	132	-0,25	-0,31	-0,34	0,16	0,55	-1,02	0,44
Mińsk Mazowiecki (2)	1383	16	31	-1,02	0,44	-0,28	1,30	-0,25	0,07	0,18
Siennica (2)	988	31	30	-0,80	-0,29	-0,41	0,48	0,34	-0,39	0,41
Stanisławów (2)	938	5	13	-1,07	-0,51	-0,24	1,24	-0,26	-0,09	0,67
Dzierzgowo (2)	546	55	58	1,00	-0,45	-0,50	-0,29	1,64	-1,27	1,07
Lipowiec Kościelny (2)	728	22	36	0,15	-0,47	-0,07	-0,48	0,56	-0,74	0,82
Radzanów (2)	474	19	67	-1,03	-0,60	0,80	0,32	-0,21	-0,67	-0,37
Strzegowo (2)	1173	72	113	0,28	-0,52	-0,55	-0,26	1,23	-0,65	1,07
Stupsk (2)	609	40	45	0,76	-0,08	0,14	-0,07	1,22	-0,84	0,90
Szreńsk (2)	569	41	45	0,39	-0,24	-0,09	-0,59	3,62	-0,70	1,28
Szydłowo (2)	575	37	44	0,68	-0,31	-0,32	-0,16	0,81	-0,91	0,77
Wieczfnia Kościelna (2)	532	39	116	0,70	-0,28	-0,32	0,55	1,97	-0,77	1,33
Wiśniewo (2)	630	26	27	-0,51	-0,34	-0,04	0,45	2,47	-0,30	0,56
Czosnów (2)	952	0	6	-0,93	0,35	-0,20	0,11	-0,24	1,90	0,54
Leoncin (2)	1376	1	8	-1,27	-0,94	0,14	0,37	-0,26	0,60	0,72
Nasielsk (3)	1665	34	115	-0,65	-0,20	-0,01	0,70	0,15	0,26	0,03
Pomiechówek (2)	667	14	28	-0,88	-0,48	-0,13	0,10	-0,10	1,09	-0,56
Zakroczym (3)	676	13	47	-1,04	-0,30	-0,09	0,25	-0,24	0,17	-0,64
Baranowo (2)	1029	55	87	0,27	-0,40	-0,53	-0,11	1,13	-1,17	2,45
Czarnia (2)	428	24	42	0,98	-0,13	0,45	0,68	0,96	-1,52	2,65
Czerwin (2)	932	153	322	0,75	-0,41	-0,75	-0,60	2,01	-0,64	1,34
Goworowo (2)	1335	76	166	-0,26	-0,71	0,07	0,18	0,58	-0,77	0,84
Kadzidło (2)	1250	55	175	0,38	-0,44	-0,36	1,34	0,99	-0,58	2,08
Lelis (2)	1051	20	57	-0,03	-0,51	-0,27	1,97	0,02	-0,53	1,99
Łyse (2)	1120	55	156	0,72	-0,47	-0,32	0,77	1,35	-1,50	2,39
Myszyniec (3)	1200	65	100	0,30	-0,24	-0,15	0,97	1,38	-0,98	2,21
Olszewo-Borki (2)	1113	32	63	-0,14	-0,67	0,41	1,59	-0,08	-0,05	1,74

Rzekuń (2)	830	27	48	-0,31	-0,08	-0,21	1,71	0,29	0,20	0,87
Troszyn (2)	871	72	182	0,55	-0,51	-0,67	-0,38	1,33	-0,69	1,37
Andrzejewo (2)	855	59	187	0,20	-0,30	-0,72	-0,85	1,83	-1,25	0,30
Boguty-Pianki (2)	577	59	185	0,91	0,15	-1,03	-1,29	1,70	-1,18	0,92
Brok (3)	433	3	10	-0,79	-1,01	-0,23	-0,83	-0,22	0,87	1,35
Małkinia Górna (2)	1369	7	21	-0,74	-0,47	-0,36	-0,26	-0,29	0,16	1,28
Nur (2)	602	24	139	0,17	-0,43	-0,79	-1,99	1,25	-0,38	1,13
Ostrów Mazowiecka (2)	1983	44	96	-0,65	-0,58	-0,40	0,21	0,00	0,08	0,48
Stary Lubotyń (2)	663	73	148	0,77	-0,27	-0,85	-0,50	1,66	-1,38	1,30
Szulborze Wielkie (2)	312	16	133	0,54	-0,13	-0,72	-1,24	1,40	-0,39	0,84
Wąsewo (2)	770	39	94	-0,30	-0,50	-0,36	-0,82	0,36	-0,43	0,57
Zaręby Kościelne (2)	674	21	53	-0,25	-0,42	-0,35	-0,98	0,65	-0,62	0,61
Celestynów (2)	625	1	4	-0,73	-0,17	-0,68	0,65	-0,59	0,84	1,47
Karczew (3)	653	2	41	-1,12	0,36	-0,23	-0,33	-0,05	1,19	-0,01
Kołbiel (2)	876	7	22	-0,93	-0,16	-0,46	0,89	-0,17	-0,13	0,49
Osieck (2)	364	0	6	-0,64	-0,38	0,07	0,35	-0,21	0,15	1,20
Sobienie-Jeziory (2)	1067	15	123	-1,24	-0,58	-0,29	0,02	-0,43	-0,13	0,09
Wiązowna (2)	798	6	9	-1,06	0,27	-0,28	1,61	0,00	2,00	0,65
Góra Kalwaria (3)	1452	30	109	-1,16	0,62	-0,03	0,20	-0,04	1,35	0,04
Konstancin-Jeziorna (3)	763	5	15	-0,41	3,08	-0,17	-1,20	0,12	2,09	-0,04
Lesznowola (2)	1099	12	16	-0,45	4,33	0,32	2,68	0,49	5,04	-0,15
Piaseczno (3)	731	12	31	-0,05	5,71	3,29	0,59	1,13	1,67	-0,08
Prażmów (2)	1011	7	20	-1,37	-0,29	0,11	1,47	-0,18	1,14	-0,15
Tarczyn (3)	1481	19	39	-1,50	0,00	0,02	0,60	0,26	1,36	-0,41
Bielsk (2)	898	35	93	0,62	0,11	-0,60	0,36	0,33	-0,74	-0,88
Bodzanów (2)	884	36	74	0,19	-0,26	-0,72	-0,64	-0,08	-0,50	-1,04
Brudzeń Duży (2)	927	40	104	0,26	-0,37	-0,46	0,63	0,20	-0,59	-0,62
Bulkowo (2)	754	52	89	0,85	-0,08	-0,83	0,11	0,49	-1,25	-0,79
Drobin (3)	724	52	74	0,24	-0,26	-0,90	0,08	0,56	-0,87	-0,25
Gąbin (3)	1006	11	43	-0,72	-0,36	-0,34	-0,60	0,28	0,48	-0,63
Łąck (2)	339	1	5	-1,03	-0,63	2,53	-0,22	-0,25	0,61	-0,31
Mała Wieś (2)	699	37	114	0,39	-0,24	-0,62	-0,74	0,26	-0,79	-0,82
Nowy Duninów (2)	427	6	3	-0,96	-0,92	1,09	-0,06	-0,41	0,15	0,63

Radzanowo (2)	817	25	54	-0,10	-0,12	-0,33	0,40	0,28	-0,27	-1,12
Słubice (2)	657	15	38	-1,00	-0,58	-0,01	-0,52	0,24	-0,29	-0,42
Słupno (2)	516	13	29	-0,44	0,37	-0,38	1,23	0,30	1,26	-1,22
Stara Biała (2)	780	42	107	0,16	0,21	-0,39	1,21	0,20	-0,16	-1,53
Staroźreby (2)	904	37	119	0,40	-0,16	-0,57	0,17	0,72	-0,82	-0,47
Wyszogród (3)	711	29	47	-0,41	-0,43	-0,66	-0,33	0,31	-0,39	-0,56
Baboszewo (2)	951	46	174	0,68	-0,26	-0,73	0,14	0,52	-0,88	-0,19
Czerwińsk nad Wisłą (2)	1490	55	156	-0,81	-0,43	-0,39	-0,63	-0,22	-0,91	-0,92
Dzierzążnia (2)	742	59	117	0,40	-0,33	-0,52	-0,29	0,19	-0,94	-0,49
Joniec (2)	625	12	26	-0,74	-0,43	-0,31	-0,46	0,08	-0,05	0,21
Naruszewo (2)	1115	45	127	-0,15	-0,44	-0,46	-0,33	0,02	-0,88	-0,50
Nowe Miasto (2)	678	14	19	-0,62	-0,84	-0,28	-0,23	0,13	-0,07	0,35
Płońsk (2)	1120	27	92	-0,60	-0,43	-0,33	0,67	-0,20	-0,13	-0,75
Raciąż (2)	1349	86	221	0,30	-0,43	-0,62	-0,09	1,22	-1,31	0,46
Sochocin (2)	908	30	65	-0,64	-0,60	-0,23	0,01	0,22	0,30	0,16
Załuski (2)	1154	38	86	-0,82	-0,31	-0,49	0,75	-0,24	-0,48	-0,60
Brwinów (3)	274	9	37	0,12	3,49	0,62	-0,21	1,70	2,11	-0,45
Michałowice (2)	151	2	8	0,97	5,29	0,45	-1,28	0,48	3,53	0,76
Nadarzyn (2)	355	2	6	-0,71	1,76	0,46	1,06	0,02	2,86	0,05
Raszyn (2)	487	6	18	-0,22	3,91	0,80	-0,61	0,81	2,85	-0,07
Chorzele (3)	1464	79	115	0,58	-0,54	0,24	0,58	0,98	-1,00	2,32
Czernice Borowe (2)	687	88	126	1,10	-0,21	-0,78	-0,43	1,13	-0,95	-0,56
Jednorożec (2)	835	52	85	0,65	-0,62	-0,33	0,51	0,82	-1,08	2,14
Krasne (2)	635	87	97	0,43	-0,48	-0,69	0,06	1,50	-0,46	-0,47
Krzynowłoga Mała (2)	658	94	144	1,25	-0,47	-0,92	0,19	2,05	-0,82	1,64
Przasnysz (2)	1306	123	212	0,07	-0,37	-0,12	0,73	1,18	-0,72	0,30
Borkowice (2)	689	7	5	-0,96	-0,27	-0,39	-0,88	-0,47	-0,06	0,69
Gielniów (2)	687	4	8	-0,76	-0,06	-0,40	-1,58	-0,46	-0,24	0,91
Klwów (2)	791	16	89	-0,98	-0,74	-0,40	-0,16	-0,32	-0,65	0,16
Odrzywół (2)	847	9	26	-0,71	-0,76	-0,40	-0,63	-0,28	0,19	0,85
Potworów (2)	930	26	116	-0,93	-0,73	-0,34	0,31	-0,37	-0,69	-0,05
Przysucha (3)	1555	9	19	-0,85	-0,49	-0,39	-1,15	-0,49	0,63	0,41
Rusinów (2)	1085	9	25	-1,00	-0,74	-0,46	-0,19	-0,35	-0,14	0,36

Wieniawa (2)	1177	11	34	-1,11	-0,74	-0,30	0,09	-0,23	0,13	0,13
Gzy (2)	693	56	128	0,35	-0,25	-0,55	-0,28	0,91	-0,92	-0,20
Obryte (2)	790	25	87	-0,68	-0,80	-0,28	0,02	0,45	-0,48	0,58
Pokrzywnica (2)	807	14	61	-0,43	-0,54	-0,12	0,76	0,46	-0,26	0,03
Pułtusk (3)	918	44	67	-0,01	0,64	-0,20	-0,07	0,30	0,43	-0,32
Świercze (2)	560	33	37	0,32	-0,27	-0,29	0,01	0,60	-1,07	0,20
Winnica (2)	587	45	143	0,42	-0,48	-0,04	0,42	0,87	-0,66	0,02
Zatory (2)	623	25	87	-0,44	-0,62	-0,24	0,61	0,43	-0,27	0,49
Gózd (2)	1063	9	7	-0,95	0,16	-0,02	2,13	-0,28	-0,80	0,07
Iłża (3)	1971	28	49	-0,83	-0,49	-0,11	-1,02	-0,27	0,04	-0,41
Jastrzębia (2)	1011	10	16	-1,15	-0,47	0,34	1,73	-0,24	-0,26	0,38
Jedlińsk (2)	1363	37	24	-1,03	0,00	0,72	1,40	-0,12	-0,43	0,04
Jedlnia-Letnisko (2)	900	9	5	-0,81	1,45	0,01	0,21	-0,05	0,06	-0,26
Kowala (2)	1350	3	15	-1,34	-0,13	-0,04	1,30	-0,40	-0,03	-0,24
Pionki (2)	1416	13	0	-0,97	-0,71	0,17	-0,03	-0,59	-0,40	0,34
Przytyk (2)	1484	22	82	-0,98	-0,77	-0,17	0,61	-0,42	-0,68	-0,10
Skaryszew (3)	1511	27	53	-0,84	-0,27	-0,23	1,57	-0,07	0,14	-0,07
Wierzbica (2)	1245	17	18	-0,87	-0,25	-0,08	0,21	-0,18	-0,38	-0,09
Wolanów (2)	1332	10	24	-1,28	-0,46	-0,15	1,56	-0,37	-0,24	-0,37
Zakrzew (2)	1077	12	25	-1,01	0,46	-0,16	1,68	-0,19	0,01	-0,51
Domanice (2)	493	13	26	-0,56	-0,37	-0,36	0,17	-0,28	-0,72	0,73
Korczew (2)	504	13	20	-0,17	-0,48	-0,26	-2,25	0,00	-0,46	0,54
Kotuń (2)	1349	16	59	-0,81	-0,69	-0,31	0,53	-0,09	-0,34	0,56
Mokobody (2)	902	44	95	0,07	-0,48	-0,53	-0,69	0,57	-0,71	0,61
Mordy (3)	1318	52	175	-0,05	-0,59	-0,53	-0,46	0,70	-0,49	0,64
Paprotnia (2)	502	45	100	0,09	-0,47	-0,54	0,01	0,89	-1,23	0,73
Przesmyki (2)	752	60	105	-0,24	-0,62	-0,58	-1,16	0,35	-0,99	0,24
Siedlce (2)	1592	65	149	-1,34	0,68	0,80	1,22	0,93	0,75	-0,09
Skórzec (2)	1077	14	31	-1,02	-0,80	-0,31	1,25	-0,01	-0,18	0,36
Suchożebry (2)	924	36	133	-0,56	-0,41	-0,48	0,41	0,38	-0,12	0,26
Wiśniew (2)	947	27	122	-0,46	-0,46	-0,50	0,41	0,27	-0,55	0,60
Wodynie (2)	737	11	31	-0,45	-0,45	-0,40	-0,59	-0,05	-0,62	0,89
Zbuczyn (2)	1947	85	173	-0,78	-0,55	-0,48	0,77	0,33	-0,23	0,07

Gozdowo (2)	650	32	48	0,61	-0,23	-0,42	-0,02	1,03	-0,94	-0,23
Mochowo (2)	770	27	60	-0,05	-0,44	0,12	0,60	0,74	-0,96	-0,19
Rościszewo (2)	734	28	56	0,20	-0,42	-0,42	-0,03	1,91	-1,01	0,60
Sierpc (2)	939	54	68	-0,01	-0,39	-0,22	0,44	0,85	-0,46	-0,23
Szczutowo (2)	623	25	19	0,08	-0,36	-0,29	-0,10	0,87	-0,70	1,00
Zawidz (2)	1011	87	105	1,00	-0,19	-0,77	-0,58	1,90	-1,43	0,81
Brochów (2)	897	2	2	-0,94	-0,95	-0,10	0,36	-0,29	0,72	0,94
Iłów (2)	1222	53	64	-0,96	-0,60	-0,40	0,09	-0,02	0,17	-0,49
Młodzieszyn (2)	985	10	43	-0,95	-0,76	-0,38	0,23	-0,18	-0,04	0,21
Nowa Sucha (2)	1007	23	34	-0,93	-0,05	-0,22	0,68	0,33	-0,30	-0,71
Rybno (2)	692	35	49	-0,98	-0,65	-0,52	1,37	0,67	-0,49	-0,73
Sochaczew (2)	1192	19	36	-1,10	0,05	-0,17	1,27	-0,08	0,40	-0,84
Teresin (2)	666	10	22	-0,16	0,43	-0,62	0,29	-0,16	0,45	-1,19
Bielany (2)	770	51	189	0,16	-0,40	-0,74	-0,09	1,57	-0,52	0,51
Ceranów (2)	496	36	70	0,19	-0,62	-0,63	-1,52	0,64	-0,61	1,44
Jabłonna Lacka (2)	1005	69	131	-0,07	-0,66	-0,78	-1,38	0,31	-0,36	-0,11
Kosów Lacki (3)	1130	16	59	-0,14	-0,75	-0,60	-1,14	0,34	-0,54	1,08
Repki (2)	1140	81	216	0,24	-0,41	-0,57	-1,56	0,75	-1,01	0,08
Sabnie (2)	786	39	83	-0,38	-0,80	-0,68	-0,58	0,19	-0,15	-0,39
Sokołów Podlaski (2)	1101	72	153	-0,33	-0,53	-0,61	-0,05	0,47	-0,10	-0,37
Sterdyń (2)	886	52	120	-0,09	-0,60	-0,81	-1,84	0,54	-0,50	0,60
Chlewiska (2)	996	2	6	-1,20	-0,55	0,71	-1,04	-0,56	-0,22	0,34
Jastrząb (2)	560	2	8	-0,79	-0,03	0,65	0,26	-0,32	-0,28	0,36
Mirów (2)	680	7	6	-1,14	-0,62	0,11	1,13	-0,45	-0,28	0,34
Orońsko (2)	789	8	20	-1,01	-0,50	0,61	0,61	-0,24	0,02	-0,02
Szydłowiec (3)	907	10	8	-1,20	0,16	1,20	-0,53	-0,11	0,51	0,30
Błonie (3)	1011	5	91	0,17	1,92	-0,60	-0,74	-0,45	1,33	-0,79
Izabelin (2)	39	2	0	8,53	2,16	4,46	-1,35	-4,33	4,58	3,46
Kampinos (2)	579	5	17	-0,88	-0,77	-0,41	0,03	-0,31	1,12	0,18
Leszno (2)	705	12	71	-0,29	-0,13	0,44	0,21	-0,50	1,13	-0,06
Łomianki (3)	344	5	7	-0,04	6,79	1,12	-0,68	0,98	2,70	0,35
Ożarów Mazowiecki (3)	918	9	83	-0,45	2,45	-0,66	0,80	0,33	1,68	-1,39
Stare Babice (2)	877	1	5	-0,98	2,24	0,07	0,65	-0,04	2,71	-0,22

Grębków (2)	1037	55	80	-0,44	-0,75	-0,51	-0,09	0,36	-0,38	0,36
Korytnica (2)	1533	75	143	-0,46	-0,66	-0,29	0,02	0,37	-0,76	0,56
Liw (2)	1213	49	115	-0,39	-0,48	-0,12	-0,09	0,42	-0,65	0,26
Łochów (3)	2252	7	5	-0,88	-0,40	-0,35	0,33	-0,38	0,26	1,48
Miedzna (2)	928	34	95	-0,44	-0,68	-0,40	-0,67	0,39	-0,40	0,46
Sadowne (2)	1050	23	26	-0,55	-0,61	-0,25	-0,23	-0,03	-0,24	1,78
Stoczek (2)	1041	20	66	-0,78	-0,75	-0,23	-0,23	-0,14	-0,36	1,34
Wierzbno (2)	771	24	32	-0,22	-0,61	-0,37	-0,63	0,20	-0,20	0,55
Dąbrówka (2)	1305	6	23	-1,08	-0,51	-0,28	1,62	-0,36	0,47	0,69
Jadów (2)	1143	3	19	-0,99	-0,58	-0,29	0,15	-0,13	0,05	0,87
Klembów (2)	1059	5	20	-0,71	0,45	-0,29	1,00	-0,35	0,22	0,79
Poświętne (2)	1278	5	5	-0,65	-0,75	-0,24	1,67	-0,80	-0,11	1,39
Radzymin (3)	1546	0	10	-0,93	0,71	0,14	2,07	-0,23	1,15	0,74
Strachówka (2)	710	5	24	-0,51	-1,00	-0,54	-0,07	-0,31	-0,38	1,70
Tłuszcz (3)	1373	6	15	-0,52	0,70	0,06	0,72	-0,41	-0,08	1,12
Wołomin (3)	798	2	6	0,55	6,92	0,24	-1,04	0,99	-0,28	1,79
Brańszczyk (2)	1608	7	50	-1,03	-0,44	0,03	-0,04	-0,19	-0,16	1,07
Długosiodło (2)	1259	9	35	-0,84	-0,74	0,03	0,33	-0,09	-0,29	1,08
Rząśnik (2)	1065	33	120	-0,69	-0,74	-0,26	0,72	0,07	-0,26	0,59
Somianka (2)	984	45	177	-0,43	-0,51	-0,63	0,38	0,15	-0,24	0,44
Wyszków (3)	1942	10	61	-0,74	1,07	0,41	0,69	0,01	0,61	0,55
Zabrodzie (2)	824	11	41	-0,76	-0,48	-0,18	0,80	-0,08	0,10	0,90
Kazanów (2)	890	17	60	-0,71	-0,75	-0,41	0,59	0,10	-0,48	0,41
Policzna (2)	1056	10	49	-0,57	-0,55	-0,21	-0,66	0,00	-0,55	-0,26
Przyłęk (2)	1381	15	25	-0,84	-0,58	0,05	-0,55	-0,33	-0,90	-0,06
Tczów (2)	828	23	75	-0,66	-0,36	-0,37	1,18	-0,01	-0,79	-0,33
Zwoleń (3)	1734	23	33	-0,97	-0,28	-0,17	-0,13	-0,08	0,19	-0,32
Bieżuń (3)	826	72	50	-1,16	-0,18	-0,23	-1,37	6,27	0,65	1,21
Kuczbork-Osada (2)	719	45	57	0,25	-0,36	-0,26	-0,26	1,35	-0,95	0,74
Lubowidz (2)	1121	36	39	-0,27	-0,67	-0,43	-0,33	0,83	-0,05	0,70
Lutocin (2)	809	57	75	-0,19	-0,29	-0,56	-0,77	3,36	-0,88	0,81
Siemiątkowo (2)	570	52	44	0,30	-0,42	-0,18	0,25	2,18	-1,12	1,49
Żuromin (3)	1299	82	63	-1,04	0,18	-0,10	-0,51	4,29	0,71	0,55

Mszczonów (3)	1481	15	92	-1,00	-0,29	-0,29	0,18	-0,32	0,52	-0,26
Puszcza Mariańska (2)	1675	1	19	-1,34	-0,59	0,06	0,02	-0,25	0,58	0,07
Radziejowice (2)	619	3	3	-1,33	-0,10	-0,35	0,82	-0,19	1,78	0,16
Wiskitki (2)	1559	18	57	-0,59	-0,52	-0,49	0,28	-0,38	0,23	-0,38
Grodków (3)	746	19	65	2,34	-0,02	-0,91	-0,55	-1,00	0,43	-1,11
Lewin Brzeski (3)	446	9	37	1,76	0,04	-0,19	-0,65	-0,95	0,83	-1,15
Lubsza (2)	678	19	24	0,42	-0,72	-0,17	-0,31	-0,97	1,00	-1,29
Olszanka (2)	643	18	36	1,06	-0,31	-0,70	-0,10	-1,23	0,14	-1,77
Baborów (3)	314	15	30	2,50	-0,05	-0,98	-0,95	-0,94	0,19	-1,68
Branice (2)	747	23	28	0,58	-0,21	-0,84	-2,03	-1,12	-0,18	-2,19
Głubczyce (3)	1760	50	106	1,04	-0,05	-0,76	-1,07	-0,66	0,75	-1,55
Kietrz (3)	948	25	34	1,30	0,18	-0,14	-1,47	-0,60	0,14	-1,29
Bierawa (2)	760	2	15	-0,39	-0,36	-0,11	-0,68	-0,65	0,20	-0,94
Cisek (2)	760	19	72	0,17	0,05	-0,39	-1,01	0,17	-0,75	-1,32
Pawłowiczki (2)	707	27	86	1,16	-0,16	-0,70	-0,85	-0,27	-0,49	-1,56
Polska Cerekiew (2)	497	9	70	0,99	0,07	-0,70	-1,70	-0,53	-0,47	-1,75
Byczyna (3)	504	24	47	1,54	-0,47	-0,70	-0,04	-0,37	0,13	-0,96
Kluczbork (3)	1201	38	145	1,15	0,93	-0,47	-1,47	-0,01	1,12	-1,22
Lasowice Wielkie (2)	512	20	95	0,51	-0,63	-0,66	-0,40	0,04	-0,22	-0,76
Wołczyn (3)	831	26	64	1,47	0,35	-0,44	-0,67	-1,13	-0,06	-1,64
Gogolin (3)	239	4	22	1,31	0,58	-0,28	-0,49	0,71	0,92	-0,37
Krapkowice (3)	357	13	31	2,17	2,16	-0,31	-2,30	1,10	1,30	-0,19
Strzeleczki (2)	924	17	49	0,02	-0,47	-0,43	-0,25	-0,05	-0,13	-0,80
Walce (2)	581	14	24	0,07	-0,15	-0,34	-0,82	0,11	-0,20	-1,00
Zdzieszowice (3)	500	2	19	0,46	1,73	-0,17	-1,12	-0,47	-0,11	-0,74
Domaszowice (2)	491	11	38	0,36	-0,71	-0,51	-0,12	0,33	0,32	-1,54
Namysłów (3)	1104	49	117	1,08	-0,09	-0,77	-0,17	-0,70	1,28	-1,41
Pokój (2)	453	4	15	-0,55	-0,74	0,34	0,22	-0,07	0,52	0,24
Głuchołazy (3)	1130	16	35	0,81	0,67	-0,54	-1,75	-1,06	1,22	-1,63
Kamiennik (2)	452	15	30	1,16	-0,54	-0,51	-1,11	-1,15	0,04	-1,44
Korfantów (3)	828	28	69	0,61	-0,62	-0,59	-0,19	-0,54	-0,42	-1,31
Łambinowice (2)	464	8	14	0,83	-0,51	-0,61	-0,83	-0,73	0,31	-1,31
Nysa (3)	1783	32	69	0,75	1,46	-0,25	-1,72	-0,59	1,40	-1,47

Otmuchów (3)	585	39	98	1,84	0,03	-0,89	-0,86	-0,55	0,36	-1,39
Paczków (3)	665	8	16	1,00	0,76	-0,84	-1,38	-0,81	0,71	-1,81
Pakosławice (2)	392	10	35	1,66	-0,39	-0,84	-0,35	-1,00	0,39	-1,58
Dobrodzień (3)	477	14	16	0,05	-0,27	-0,47	-0,93	1,73	0,45	0,28
Gorzów Śląski (3)	656	39	60	0,89	-0,27	-0,63	-0,90	0,18	-0,16	-0,74
Olesno (3)	984	40	129	0,43	-0,11	-0,62	-1,03	0,42	1,10	-0,54
Praszka (3)	1305	15	15	-0,61	0,21	-0,56	-0,96	-0,03	0,64	-0,37
Radłów (2)	504	39	77	0,15	-0,76	-0,59	0,11	0,19	-0,06	-0,62
Rudniki (2)	1441	21	41	0,00	-0,05	-0,49	-0,93	0,23	0,40	-1,36
Zębowice (2)	271	15	27	-0,13	-0,71	-0,34	-0,39	0,02	-0,25	-0,14
Chrząstowice (2)	309	10	41	0,10	0,11	-0,31	-0,73	0,55	1,05	0,33
Dąbrowa (2)	258	9	36	1,61	0,19	-0,39	-0,61	0,58	1,01	-0,58
Dobrzeń Wielki (2)	233	9	15	0,89	1,37	-0,24	-0,63	0,97	0,71	-0,13
Komprachcice (2)	374	11	7	-0,29	1,16	-0,31	-0,63	0,04	0,76	-0,89
Łubniany (2)	362	11	24	0,10	-0,13	-0,16	-0,39	0,55	1,24	-0,24
Murów (2)	617	2	4	-0,80	-0,78	0,19	-1,88	-0,42	0,55	0,44
Niemodlin (3)	407	6	26	1,74	-0,06	-0,54	-0,65	1,43	1,80	-0,81
Ozimek (3)	351	0	15	0,13	0,66	-0,09	-1,23	-0,04	0,58	0,69
Popielów (2)	385	8	28	0,41	-0,44	-0,13	-0,66	0,45	0,52	-0,22
Prószków (3)	404	15	40	0,37	0,27	-0,20	-0,56	1,34	0,75	-0,49
Tarnów Opolski (2)	215	9	13	0,58	0,25	0,13	-0,48	-0,40	0,29	-1,15
Tułowice (2)	91	2	3	0,72	-0,11	-0,42	-0,63	-0,13	1,64	0,12
Turawa (2)	355	7	39	0,12	-0,23	-0,05	-0,66	0,38	1,18	0,07
Biała (3)	938	100	294	0,92	-0,35	-0,76	-0,71	0,80	-0,27	-1,26
Głogówek (3)	1117	58	172	0,94	0,16	-0,71	-0,68	0,63	0,22	-1,35
Lubrza (2)	387	19	50	1,21	-0,20	-0,73	-0,46	0,18	-0,04	-1,70
Prudnik (3)	703	29	43	1,25	1,26	-0,36	-1,70	-0,60	0,42	-1,52
Izbicko (2)	628	12	41	-0,45	-0,53	-0,31	0,14	0,40	0,23	-0,19
Jemielnica (2)	545	10	26	-0,45	-0,67	-0,25	0,24	-0,06	-0,32	0,10
Kolonowskie (3)	708	3	5	-0,92	-0,42	-0,16	-0,93	-0,41	-0,14	0,67
Leśnica (3)	824	15	71	0,49	-0,01	-0,66	-0,66	-0,26	-0,22	-1,21
Strzelce Opolskie (3)	612	31	103	2,05	2,11	-0,74	-1,22	-0,63	0,22	-1,16
Ujazd (3)	690	34	67	0,06	-0,17	-0,51	0,42	-0,60	-0,51	-1,30

Zawadzkie (3)	544	3	11	-0,50	0,23	0,02	-1,58	0,10	-0,09	0,32
Czarna (2)	337	21	40	0,47	-0,93	-0,42	0,63	-0,60	0,82	2,87
Lutowiska (2)	156	1	9	1,14	-1,26	3,85	-1,39	-0,31	3,71	1,91
Ustrzyki Dolne (3)	1889	8	47	-0,19	-0,75	0,84	-0,34	-0,61	0,90	2,12
Brzozów (3)	4729	6	9	0,16	3,03	-0,28	-0,26	-1,07	-1,08	1,37
Domaradz (2)	1585	1	0	-0,43	1,16	-0,47	-0,16	-1,28	-1,01	1,01
Dydnia (2)	1631	3	3	-0,31	0,46	-0,34	0,00	-1,25	-0,66	1,20
Haczów (2)	1968	7	19	0,15	1,98	-0,07	-0,61	-1,04	-0,74	0,91
Jasienica Rosielna (2)	1696	1	5	-0,24	1,19	-0,52	0,45	-1,44	-0,88	1,29
Nozdrzec (2)	1876	6	5	-0,40	0,56	-0,27	-0,97	-1,01	-0,70	1,18
Brzostek (3)	2348	7	11	-0,47	0,88	-0,34	-0,04	-1,00	-1,02	0,74
Czarna (2)	1692	6	14	2,38	4,25	-2,91	0,11	-3,77	-1,98	5,47
Dębica (2)	4454	11	27	-0,32	2,46	-0,30	0,37	-0,79	-0,86	0,67
Jodłowa (2)	1073	7	19	-0,57	0,28	-0,39	0,08	-0,95	-1,09	0,35
Pilzno (3)	2854	7	6	-0,62	1,24	-0,32	0,19	-0,70	-0,61	0,20
Żyraków (2)	2100	16	40	-0,28	1,93	-0,45	0,47	-0,83	-0,86	0,21
Chłopice (2)	1016	15	39	0,17	1,83	-0,35	-0,99	-1,30	-1,65	-1,01
Jarosław (2)	1843	46	98	-0,05	1,27	-0,27	0,34	-0,90	-0,64	-0,93
Laszki (2)	1418	11	43	-0,54	-0,40	0,05	0,73	-0,76	-0,77	-0,50
Pawłosiów (2)	1377	10	30	0,21	3,14	-0,06	-0,46	-0,70	-0,96	-1,31
Radymno (2)	2047	28	97	-0,21	0,43	-0,09	-0,09	-1,11	-0,85	-0,46
Rokietnica (2)	929	18	25	-0,27	1,03	0,26	-0,44	-1,16	-1,22	-1,44
Roźwienica (2)	1126	14	37	-0,36	1,32	0,13	0,51	-1,04	-0,99	-1,11
Wiązownica (2)	2066	10	24	-0,65	-0,26	0,02	0,76	-0,82	-0,18	-0,08
Brzyska (2)	1265	0	0	-0,24	1,45	-0,62	0,26	-1,35	-1,18	0,90
Dębowiec (2)	1747	4	6	-0,71	0,79	0,01	0,26	-0,99	-0,73	0,84
Jasło (2)	3432	6	12	0,00	2,32	0,19	-0,83	-1,27	-0,76	0,77
Kołaczyce (3)	1751	2	0	-0,15	2,02	-0,25	-0,06	-1,25	-1,16	1,02
Krempna (2)	418	5	28	0,30	-0,45	4,62	-0,43	-0,56	-0,92	2,12
Nowy Żmigród (2)	1854	3	8	-0,42	0,85	0,64	-0,37	-0,98	-0,72	1,58
Osiek Jasielski (2)	1127	4	7	-0,45	0,91	0,26	0,17	-1,06	-1,09	1,45
Skołyszyn (2)	2580	1	9	-0,40	1,79	-0,14	-0,39	-1,10	-0,94	0,87
Tarnowiec (2)	1893	8	14	-0,45	1,98	0,07	-0,43	-0,83	-1,06	0,62

Cmolas (2)	1177	6	26	-0,42	0,36	-0,34	0,11	-0,92	-0,67	1,52
Kolbuszowa (3)	2569	15	61	-0,65	0,89	-0,10	-0,51	-0,54	0,15	0,67
Majdan Królewski (2)	1731	5	21	-0,67	0,30	-0,19	0,32	-0,90	-0,68	1,30
Niwiska (2)	1094	0	20	-0,71	0,49	-0,32	0,45	-0,84	-0,41	1,11
Raniżów (2)	1288	7	24	-0,65	0,30	-0,05	0,12	-0,73	-0,70	0,99
Dzikowiec (2)	1118	13	32	-0,66	0,00	-0,25	0,34	-0,87	-0,77	0,90
Chorkówka (2)	2727	1	0	-0,35	2,27	-0,43	0,18	-0,98	-0,84	0,73
Dukla (3)	2606	11	19	-0,49	0,26	0,19	-0,06	-0,98	-0,43	1,76
Iwonicz-Zdrój (3)	1119	9	10	0,35	2,85	0,33	-0,78	-1,12	-0,35	1,53
Jedlicze (3)	2688	5	11	0,12	3,36	-0,09	-0,98	-0,93	-1,03	0,43
Korczyna (2)	2165	9	15	-0,48	1,24	-0,31	-0,14	-0,90	-0,12	0,62
Krościenko Wyżne (2)	1019	3	3	0,06	3,42	0,79	-0,27	-0,71	-0,26	1,02
Miejsce Piastowe (2)	2124	6	13	-0,02	3,64	0,27	-0,71	-0,71	-0,75	0,37
Rymanów (3)	2606	21	71	-0,23	0,96	-0,01	-0,24	-0,73	0,20	0,89
Wojaszówka (2)	1904	6	3	-0,50	0,85	-0,29	-0,24	-1,08	-0,17	0,74
Jaśliska (2)	348	1	2	-0,35	-0,62	-0,01	-0,08	-1,03	-0,45	1,69
Grodzisko Dolne (2)	1501	11	16	-0,97	0,77	0,27	-0,18	-0,78	-0,97	-0,36
Kuryłówka (2)	1024	14	93	-0,60	-0,47	0,57	0,54	-0,63	-0,37	0,17
Leżajsk (2)	3116	20	77	-0,60	0,61	0,08	0,35	-0,76	-0,74	-0,42
Nowa Sarzyna (3)	2717	5	18	-0,83	0,96	0,32	-0,10	-0,70	-0,83	0,52
Cieszanów (3)	969	22	113	0,15	-0,28	-0,10	-0,48	-0,89	-0,46	-0,59
Horyniec-Zdrój (2)	927	8	18	-0,41	-1,10	-0,44	-0,93	-0,86	0,11	-0,11
Lubaczów (2)	1612	12	42	-0,59	-0,20	0,14	0,47	-0,85	-0,62	0,10
Narol (3)	1361	21	57	-0,70	-0,61	-0,06	-0,42	-0,66	-0,25	-0,41
Oleszyce (3)	990	16	51	-0,29	-0,26	0,29	-0,03	-1,02	0,05	-0,73
Stary Dzików (2)	869	33	155	-0,13	-0,43	0,44	-1,26	-0,68	-0,94	0,22
Wielkie Oczy (2)	753	13	59	-0,55	-0,59	0,38	0,07	-0,67	-0,62	0,28
Białobrzegi (2)	1627	12	54	-0,73	2,31	-0,10	0,90	-0,67	-0,85	-0,10
Czarna (2)	2061	8	16	-0,78	0,78	0,99	0,80	-0,29	-0,15	-0,62
Łańcut (2)	3227	26	73	-0,37	2,86	0,31	0,08	-0,57	-0,87	-0,77
Markowa (2)	1364	27	67	0,39	0,79	-0,27	0,32	-0,53	-0,80	-0,52
Rakszawa (2)	845	2	0	-0,78	0,53	-0,61	0,36	-0,67	-0,35	1,15
Żołynia (2)	834	3	6	-0,85	1,04	-0,16	0,40	-0,57	-0,53	0,48

Borowa (2)	1077	15	42	-0,97	0,38	-0,42	0,01	-0,10	-0,72	-0,99
Czermin (2)	1398	20	36	-1,08	0,12	-0,28	0,81	-0,33	-0,59	-0,81
Gawłuszowice (2)	584	6	19	-1,42	-0,20	-0,23	0,80	0,27	-0,74	-0,56
Mielec (2)	2203	18	40	-0,91	0,70	0,09	0,39	-0,51	-0,04	-0,67
Padew Narodowa (2)	1075	21	31	-0,44	0,44	-0,22	-0,74	-0,62	-0,43	0,12
Przecław (3)	2063	5	17	-1,05	-0,02	-0,35	1,22	-0,80	-0,69	-0,01
Radomyśl Wielki (3)	2473	14	31	-1,07	0,29	-0,28	0,79	-0,18	-0,20	0,09
Tuszów Narodowy (2)	1576	12	12	-0,99	0,05	-0,29	0,64	-0,69	-0,21	0,21
Wadowice Górne (2)	1268	14	54	-1,15	0,45	-0,18	0,92	-0,11	-0,57	-0,60
Harasiuki (2)	1301	1	5	-0,75	-0,85	-0,69	-0,27	-0,97	-0,28	1,28
Jarocin (2)	1025	73	115	-0,69	-0,22	-0,53	0,00	-0,78	-0,45	1,41
Jeżowe (2)	1767	2	16	-0,52	0,67	-0,14	0,48	-0,91	-0,85	1,07
Krzeszów (2)	790	10	8	-1,01	0,06	-0,12	-0,65	-0,55	-0,20	0,08
Nisko (3)	1732	4	7	-0,67	1,17	0,24	-0,30	-0,77	-0,10	0,74
Rudnik nad Sanem (3)	1565	4	9	-0,64	0,99	-0,10	-0,95	-0,79	-0,29	0,46
Ulanów (3)	1541	0	12	-0,59	0,28	-0,26	-0,97	-0,90	-0,42	0,95
Bircza (2)	1253	9	17	-0,52	-0,83	0,67	0,03	-0,93	-0,42	1,14
Dubiecko (2)	1958	2	3	-0,76	-0,20	-0,36	-0,33	-1,04	-0,52	0,50
Fredropol (2)	1166	11	26	-0,35	-0,39	1,12	0,09	-1,15	-0,89	-0,31
Krasiczyn (2)	749	3	2	-0,68	-0,33	1,54	0,20	-0,97	0,11	0,19
Krzywcza (2)	1171	1	0	-0,77	-0,64	-0,50	0,48	-1,17	-0,44	0,53
Medyka (2)	885	13	31	0,03	0,95	-0,34	0,40	-1,46	-0,77	-1,44
Orły (2)	1552	17	42	-0,03	1,89	0,38	0,35	-1,17	-1,24	-1,53
Przemyśl (2)	1724	29	58	-0,37	0,97	0,69	0,42	-1,15	-0,26	-0,98
Stubno (2)	684	7	33	0,10	0,05	0,62	0,08	-0,89	-0,74	-0,89
Żurawica (2)	2218	15	42	-0,14	1,58	0,31	-0,30	-1,15	-1,16	-1,30
Adamówka (2)	824	16	34	-0,67	-0,66	1,22	-0,09	-0,53	-0,54	0,40
Gać (2)	665	25	85	0,45	2,24	-0,37	-0,52	-0,92	-1,52	-1,39
Jawornik Polski (2)	1069	5	4	-0,64	0,16	-0,17	-1,01	-0,99	-0,16	0,65
Kańczuga (3)	2597	17	44	-0,44	1,62	0,96	-0,63	-0,93	-1,20	-0,96
Przeworsk (2)	2731	34	68	-0,06	2,93	1,12	-0,47	-0,97	-1,60	-0,94
Sieniawa (3)	1097	15	25	-0,84	-0,01	1,06	0,66	-0,66	-0,73	0,15
Tryńcza (2)	1599	10	19	-0,47	1,63	0,36	0,18	-0,77	-0,93	-0,17

Zarzecze (2)	1265	33	36	-0,44	2,18	0,60	0,49	-0,74	-1,47	-1,17
Iwierzyce (2)	944	15	30	-0,79	0,76	-0,32	0,45	-0,43	-0,72	-0,52
Ostrów (2)	927	5	12	-0,41	0,81	-0,60	0,68	-0,75	-0,49	1,28
Ropczyce (3)	1654	14	36	-0,25	2,15	-0,43	0,40	-0,49	-0,42	0,31
Sędziszów Małopolski (3)	1757	3	26	-0,52	1,21	-0,24	0,00	-0,49	-0,29	0,37
Wielopole Skrzyńskie (2)	1353	10	23	-0,64	-0,11	-0,39	0,22	-0,82	-0,43	0,51
Błażowa (3)	2531	6	13	-0,86	0,07	-0,38	-0,29	-0,89	-0,25	0,58
Boguchwała (3)	3195	1	26	-0,64	3,07	0,00	0,32	-0,44	-0,43	-0,41
Chmielnik (2)	1679	12	32	-0,45	1,51	0,25	0,21	-1,08	-0,69	0,89
Dynów (2)	1498	11	29	-0,30	0,49	0,15	-0,21	-0,86	-0,90	0,74
Głogów Małopolski (3)	2218	2	22	-0,77	1,11	0,64	0,67	-0,65	0,13	0,71
Hyżne (2)	1638	1	5	-0,84	1,56	0,30	0,03	-0,78	-1,14	0,38
Kamień (2)	1274	6	10	-0,72	0,67	0,06	0,31	-0,76	-0,85	0,63
Krasne (2)	1686	6	14	-0,33	3,83	-0,13	0,21	-0,53	-0,27	0,18
Lubenia (2)	1373	1	0	-0,57	1,09	-0,19	-0,97	-0,89	-0,75	0,98
Sokołów Małopolski (3)	2900	6	23	-0,67	1,33	-0,18	0,26	-0,60	-0,85	0,68
Świlcza (2)	3241	2	10	-0,47	1,62	-0,02	0,15	-1,08	-0,43	-0,61
Trzebownisko (2)	3635	6	19	-0,29	3,16	-0,19	0,68	-0,66	-0,56	0,25
Tyczyn (3)	2441	0	5	-0,72	1,55	0,09	0,70	-0,85	-0,07	0,31
Besko (2)	404	2	22	0,56	2,10	0,23	0,55	-0,53	-0,98	0,69
Bukowsko (2)	1000	21	85	-0,38	-0,30	0,54	0,83	-0,40	0,02	1,29
Komańcza (2)	686	7	36	0,36	-0,82	3,33	-1,05	-0,51	0,15	1,13
Sanok (2)	3120	18	31	-0,51	0,58	-0,21	0,23	-0,99	-0,44	0,85
Tyrawa Wołoska (2)	413	3	2	-0,38	-0,32	0,89	0,73	-1,15	-0,75	1,48
Zagórz (3)	2273	2	27	-0,49	0,24	0,19	0,05	-1,01	-0,25	1,52
Zarszyn (2)	1692	12	31	-0,14	1,01	0,58	-0,46	-0,57	-0,83	0,49
Bojanów (2)	1351	2	6	-0,70	-0,40	-0,35	0,66	-1,15	-0,50	1,15
Pysznica (2)	1747	0	2	-1,27	-0,14	-0,17	0,49	-0,86	0,22	0,09
Radomyśl nad Sanem (2)	1126	4	31	-0,82	-0,05	-0,24	-0,64	-0,61	0,01	0,29
Zaleszany (2)	2164	6	1	-0,78	1,41	-0,21	-0,28	-0,73	-0,41	-0,06
Czudec (2)	2115	4	10	-0,67	1,32	-0,40	0,07	-0,65	-0,48	0,11
Frysztak (2)	1870	2	4	-0,34	1,36	-0,47	-0,21	-0,99	-0,61	0,81
Niebylec (2)	1917	3	0	-0,43	0,96	-0,69	0,12	-1,05	-0,81	1,24

Strzyżów (3)	3220	7	24	-0,38	1,21	-0,51	-0,54	-0,92	-0,25	0,68
Wiśniowa (2)	1480	9	14	-0,47	0,76	-0,34	-0,16	-0,92	-0,65	0,26
Baranów Sandomierski (3)	2210	10	11	-0,92	0,61	-0,03	-0,44	-0,58	-0,28	0,44
Gorzyce (2)	1564	6	20	-0,41	2,23	-0,02	-0,73	-0,63	-0,69	-0,04
Grębów (2)	1534	3	6	-0,83	0,13	-0,20	0,13	-0,71	-0,43	0,59
Nowa Dęba (3)	1601	7	16	-0,37	0,91	0,06	-1,21	-0,68	0,00	0,44
Baligród (2)	553	3	19	-0,05	-0,85	0,72	-0,48	-0,80	1,41	2,19
Cisna (2)	260	1	0	-0,17	-1,75	-0,60	-0,09	-0,77	4,58	2,00
Lesko (3)	1358	1	6	-0,43	0,69	1,46	-0,88	-0,63	0,89	1,45
Olszanica (2)	935	4	18	-0,65	-0,34	2,87	-0,43	-0,58	0,17	1,61
Solina (2)	841	1	11	-0,85	-0,93	1,79	-1,05	-0,28	3,95	1,74
Augustów (2)	1306	88	187	0,75	-0,34	-0,01	0,34	1,35	-1,19	1,15
Bargłów Kościelny (2)	999	74	179	0,47	-0,34	-0,10	-0,10	1,71	-1,52	1,04
Lipsk (3)	943	31	70	0,20	-0,58	0,17	-1,55	0,35	-0,79	1,13
Nowinka (2)	605	15	29	-0,42	-0,74	1,53	-0,19	0,25	0,17	0,86
Płaska (2)	740	3	4	-0,86	-1,48	-0,17	-0,86	-0,47	1,61	1,20
Sztabin (2)	951	70	149	1,02	-0,44	1,01	-1,17	1,12	-1,07	1,93
Choroszcz (3)	1954	12	13	-1,04	-0,75	0,02	0,66	0,00	0,79	0,46
Czarna Białostocka (3)	486	15	12	-0,50	-0,84	0,17	-0,50	0,01	0,36	0,68
Dobrzyniewo Duże (2)	1286	25	22	-0,15	-0,72	0,19	0,88	-0,29	0,96	0,18
Gródek (2)	1107	12	24	-0,13	-1,08	0,26	-2,12	-0,11	0,40	1,32
Juchnowiec Kościelny (2)	1874	20	24	-0,71	-0,44	-0,05	1,56	-0,22	1,08	0,03
Łapy (3)	1408	49	59	-0,20	0,57	-0,28	-1,29	0,44	-0,04	0,73
Michałowo (3)	1493	32	42	-0,30	-0,81	0,68	-2,51	0,27	0,20	0,88
Poświętne (2)	705	39	126	0,19	-0,22	-0,75	-0,60	2,00	-0,99	0,73
Supraśl (3)	948	4	9	-0,49	-0,70	0,60	1,09	-0,80	1,81	-0,41
Suraż (3)	640	11	13	-0,49	-0,79	-0,30	-0,94	0,46	0,28	0,77
Turośń Kościelna (2)	845	13	14	-0,82	-0,69	-0,27	1,09	0,54	0,92	0,50
Tykocin (3)	985	60	96	0,57	-0,62	-0,73	-0,85	1,13	-0,81	1,21
Wasilków (3)	1013	4	9	-1,49	-0,36	1,07	1,64	0,48	1,42	0,41
Zabłudów (3)	1823	28	37	-0,46	-1,00	0,17	-0,15	-0,12	0,40	0,62
Zawady (2)	633	23	109	0,95	-0,49	-0,97	-0,85	1,42	-1,23	1,74
Bielsk Podlaski (2)	3133	87	125	-0,67	-0,92	0,44	-2,69	0,18	0,18	0,20

Boćki (2)	1222	50	124	0,26	-0,63	-0,23	-2,44	0,92	-0,67	1,02
Brańsk (2)	1258	110	191	0,58	-0,44	-0,66	-1,17	1,44	-1,33	1,11
Orla (2)	1206	12	15	-0,62	-0,86	-0,47	-4,56	0,13	0,49	0,14
Rudka (2)	318	14	21	-0,02	-0,60	-0,48	-0,77	0,37	-0,41	1,02
Wyszki (2)	1225	51	78	0,10	-0,69	-0,34	-1,31	0,83	-0,82	0,91
Grajewo (2)	982	86	164	1,39	-0,45	0,56	0,07	1,75	-1,70	1,90
Radziłów (2)	744	79	178	1,26	-0,31	-0,12	-0,67	2,00	-1,78	1,67
Rajgród (3)	784	39	93	0,90	-0,46	0,23	-0,94	1,27	-0,48	1,65
Szczuczyn (3)	621	67	150	1,04	-0,27	-0,20	-0,45	1,82	-1,08	1,56
Wąsosz (2)	632	62	139	0,63	-0,38	-0,23	-0,47	1,07	-1,61	1,24
Białowieża (2)	458	0	0	-1,95	-1,08	4,76	-2,67	-0,32	2,14	-0,06
Czeremcha (2)	921	2	5	-1,09	-0,78	0,47	-2,36	-0,22	-0,27	0,95
Czyże (2)	853	21	18	-0,17	-0,59	-0,46	-4,89	0,49	0,12	0,04
Dubicze Cerkiewne (2)	675	7	8	-0,52	-0,75	-0,41	-6,39	0,59	1,77	1,14
Hajnówka (2)	1123	40	42	-0,83	-0,98	0,55	-1,81	0,01	0,46	-0,28
Kleszczele (3)	716	2	4	-0,93	-0,92	0,20	-3,32	0,00	0,51	0,92
Narew (2)	1051	12	34	-0,51	-0,76	-0,19	-3,61	1,62	0,66	0,71
Narewka (2)	1101	4	2	-0,68	-0,94	0,15	-2,97	-0,33	0,22	1,56
Grabowo (2)	555	73	217	1,55	-0,31	-0,82	-0,33	2,17	-1,52	1,82
Kolno (2)	1385	158	414	0,87	-0,36	0,35	0,24	1,96	-1,44	1,52
Mały Płock (2)	849	51	111	0,26	-0,50	-0,43	-0,51	0,86	-0,92	1,10
Stawiski (3)	831	66	190	1,10	-0,39	-0,36	-0,40	1,85	-1,11	1,49
Turośl (2)	780	34	109	1,02	-0,52	-0,52	0,89	2,09	-1,26	2,59
Jedwabne (3)	1005	56	158	0,35	-0,68	-0,13	-0,31	0,93	-1,13	1,17
Łomża (2)	1564	102	189	-0,20	-0,60	0,15	0,62	0,47	-0,03	0,75
Miastkowo (2)	658	31	67	0,23	-0,72	-0,33	0,64	0,43	-0,93	1,08
Nowogród (3)	487	20	34	0,25	-0,73	-0,33	-0,25	0,52	-0,21	1,10
Piątnica (2)	1689	64	94	-0,28	-0,69	-0,13	0,11	0,36	-0,25	0,62
Przytuły (2)	360	31	66	1,23	-0,10	-0,30	-0,39	1,63	-1,70	1,56
Śniadowo (2)	892	83	259	0,93	-0,41	-0,81	-0,42	2,51	-0,99	1,31
Wizna (2)	716	44	167	1,03	-0,31	0,04	-1,10	1,52	-1,26	1,65
Zbójna (2)	622	14	61	0,47	-0,61	-0,08	-0,26	1,04	-0,73	2,18
Goniądz (3)	773	45	49	0,87	-0,88	-0,27	-0,66	1,03	-0,44	1,70

Jasionówka (2)	603	29	49	0,28	-0,52	-0,46	-1,16	0,98	-0,88	1,00
Jaświły (2)	938	88	100	0,89	-0,26	-0,46	-1,91	1,24	-1,27	1,20
Knyszyn (3)	567	32	21	0,31	-0,57	-0,61	-0,74	1,11	-0,23	1,03
Krypno (2)	629	24	53	0,23	-0,32	-0,28	-0,49	2,14	-0,49	1,37
Mońki (3)	1208	72	65	-0,04	-0,28	-0,06	-0,88	1,20	-0,07	0,63
Trzcianne (2)	846	40	49	0,43	-0,86	-0,35	-0,87	0,74	-0,97	1,48
Giby (2)	663	21	23	-0,42	-1,08	1,19	-1,41	0,05	0,14	0,71
Krasnopol (2)	1063	48	63	-0,40	-0,89	2,18	0,03	0,60	-0,86	0,76
Puńsk (2)	667	68	94	0,89	-0,10	1,37	-0,91	2,55	-1,26	1,42
Sejny (2)	964	84	138	0,27	-0,65	0,72	-0,84	1,32	-0,65	0,98
Drohiczyn (3)	1166	64	120	0,28	-0,41	-0,10	-1,96	1,19	-0,48	0,31
Dziadkowice (2)	669	45	49	0,38	-0,52	-0,34	-1,83	0,60	-0,94	0,68
Grodzisk (2)	1068	60	129	0,26	-0,64	-0,61	-1,54	1,02	-1,13	0,68
Mielnik (2)	579	2	3	-0,49	-0,89	-0,43	-3,16	-0,26	0,38	1,33
Milejczyce (2)	647	6	28	0,04	-0,70	-0,36	-3,85	0,20	-0,29	1,41
Nurzec-Stacja (2)	1025	5	3	-0,46	-0,92	-0,24	-3,26	-0,22	-0,09	0,70
Perlejewo (2)	633	32	114	0,57	-0,35	-0,41	-1,88	1,17	-0,90	0,61
Siemiatycze (2)	1721	57	76	-0,62	-0,96	-0,09	-0,88	0,08	-0,28	0,38
Dąbrowa Białostocka (3)	1453	82	95	0,46	-0,17	0,12	-1,75	0,78	-0,62	0,99
Janów (2)	811	68	81	0,65	-0,49	0,14	-1,65	1,20	-1,03	1,00
Korycin (2)	575	50	106	0,88	-0,36	-0,43	-0,87	1,72	-1,23	1,20
Krynki (3)	282	5	20	0,38	-0,76	0,78	-2,58	-0,33	0,11	0,17
Kuźnica (2)	683	14	14	-0,50	-0,59	1,64	-1,16	0,79	-0,48	0,59
Nowy Dwór (2)	598	36	29	0,29	-0,52	0,13	-1,28	0,69	-1,33	0,83
Sidra (2)	752	31	49	0,23	-0,60	0,60	-0,98	0,77	-1,13	0,91
Sokółka (3)	1695	47	66	0,01	-0,32	0,99	-0,80	0,29	-0,11	0,77
Suchowola (3)	1080	75	199	0,63	-0,35	0,25	-1,45	1,78	-1,05	1,26
Szudziałowo (2)	716	20	41	0,34	-0,96	-0,40	-1,77	0,04	-0,27	0,17
Bakałarzewo (2)	535	39	56	0,64	-0,39	0,64	0,94	1,82	-1,35	0,94
Filipów (2)	728	45	74	0,36	-0,45	2,17	-0,11	1,23	-1,51	0,57
Jeleniewo (2)	712	40	78	0,28	-0,43	2,09	0,30	1,12	-1,11	0,89
Przerośl (2)	633	38	47	0,68	-0,12	3,30	-0,60	1,49	-1,61	1,20
Raczki (2)	883	44	64	-0,06	-0,45	1,87	0,71	1,14	-1,29	0,37

Rutka-Tartak (2)	513	25	35	0,55	-0,35	1,55	-0,39	1,50	-1,62	1,63
Suwałki (2)	1256	53	43	-0,42	-0,68	2,66	1,18	0,68	0,21	0,37
Szypliszki (2)	824	39	45	0,41	-0,38	2,04	0,22	1,29	-1,25	1,13
Wiżajny (2)	561	44	62	0,47	-0,19	3,13	-0,66	1,73	-1,41	1,13
Ciechanowiec (3)	1292	59	113	0,12	-0,62	-0,35	-1,36	0,57	0,21	0,87
Klukowo (2)	851	50	181	0,65	-0,24	-0,90	-1,20	1,67	-1,11	0,78
Kobylin-Borzymy (2)	628	45	88	1,03	-0,25	-1,09	-0,79	2,13	-1,53	1,15
Kulesze Kościelne (2)	554	52	105	1,25	-0,31	-1,22	-0,44	2,64	-1,59	2,02
Nowe Piekuty (2)	759	73	196	0,61	-0,36	-0,95	-0,80	1,72	-0,91	0,87
Sokoły (2)	898	60	130	0,31	-0,46	-0,76	-0,36	1,37	-0,90	0,80
Szepietowo (3)	870	60	194	0,59	-0,37	-0,93	-0,82	1,89	-0,60	0,25
Wysokie Mazowieckie (2)	1032	78	168	0,16	-0,50	-0,62	0,38	1,57	-1,02	0,26
Kołaki Kościelne (2)	466	33	117	0,94	-0,16	-0,94	-0,99	1,74	-0,97	1,36
Rutki (2)	837	67	92	1,05	-0,45	-0,85	-0,74	1,50	-1,23	1,49
Szumowo (2)	794	48	65	0,35	-0,50	-0,54	0,09	1,27	-0,86	0,47
Zambrów (2)	1514	73	186	-0,04	-0,71	-0,70	0,56	1,14	-0,53	0,37
Borzytuchom (2)	347	4	17	0,03	-0,79	0,30	2,03	-0,32	-0,31	0,16
Bytów (3)	1030	11	38	0,10	-0,02	0,30	0,93	-0,33	0,94	0,35
Czarna Dąbrówka (2)	689	29	18	0,10	-1,08	0,37	1,68	-0,26	-0,07	0,18
Kołczygłowy (2)	310	7	31	0,21	-0,56	3,71	1,02	-0,29	-0,79	-0,44
Lipnica (2)	742	44	39	1,07	-1,01	-0,41	1,17	0,00	-0,17	1,76
Miastko (3)	613	20	40	0,73	-0,57	2,27	-0,19	0,41	0,50	0,32
Parchowo (2)	441	17	23	0,00	-1,05	-0,60	2,33	0,25	0,14	1,05
Studzienice (2)	322	9	13	0,57	-1,03	-0,73	1,27	0,06	0,44	1,28
Trzebielino (2)	324	6	9	1,18	-0,96	0,37	0,72	-0,47	0,46	0,32
Tuchomie (2)	547	13	48	-0,02	-0,54	1,37	1,54	-0,13	-0,54	0,27
Brusy (3)	926	54	179	0,64	-0,82	-0,76	1,83	1,02	0,12	1,37
Chojnice (2)	977	102	201	0,98	-0,63	-0,54	1,85	0,47	0,00	-0,15
Czersk (3)	1082	31	60	-0,16	-0,65	-0,36	0,80	0,25	0,36	0,94
Konarzyny (2)	241	17	43	0,81	-0,69	-0,36	1,36	0,57	-0,64	1,04
Czarne (3)	259	12	26	2,18	-0,41	2,37	-0,15	-0,55	-0,10	-0,64
Człuchów (2)	765	50	109	1,01	-0,82	0,64	1,76	-0,71	-0,20	-0,70
Debrzno (3)	456	40	98	1,75	-0,49	-0,10	-0,01	-0,34	0,03	-0,41

Koczała (2)	219	2	8	0,02	-1,14	2,85	0,24	-0,49	0,30	0,02
Przechlewo (2)	448	15	40	2,35	-0,29	-0,20	0,58	6,45	0,23	0,48
Rzeczenica (2)	421	9	13	-0,10	-0,94	2,49	0,48	-0,48	-0,22	0,55
Cedry Wielkie (2)	352	21	51	2,14	-0,22	-0,72	1,87	-0,85	-0,49	-1,28
Kolbudy (2)	704	0	6	-1,03	1,17	0,54	1,86	-0,44	1,87	-0,17
Pruszcz Gdański (2)	756	29	62	0,09	0,63	-0,06	3,60	-0,63	1,81	-1,82
Przywidz (2)	651	3	6	-0,73	-0,99	0,43	1,14	-0,13	0,95	0,48
Pszczółki (2)	371	12	27	0,94	1,14	-0,59	1,50	-1,11	0,45	-1,69
Suchy Dąb (2)	326	20	38	1,19	-0,32	-0,57	1,37	-0,90	-0,37	-1,34
Trąbki Wielkie (2)	641	8	17	-0,05	-0,52	0,37	1,60	-0,76	0,02	-0,75
Chmielno (2)	652	12	51	-0,95	-0,28	-0,63	2,52	1,30	0,53	0,68
Kartuzy (3)	1333	31	89	-1,08	0,37	0,45	1,21	1,07	0,76	0,41
Przodkowo (2)	770	5	30	-1,05	0,08	0,07	3,48	0,58	0,31	0,29
Sierakowice (2)	1163	36	123	-0,64	-0,35	-0,53	3,52	1,58	0,16	0,72
Somonino (2)	554	5	34	-0,54	-0,46	-0,17	2,79	0,16	0,08	0,58
Stężyca (2)	1002	24	68	-0,53	-0,70	-0,40	2,69	0,09	0,49	0,75
Sulęczyno (2)	648	5	16	-0,21	-0,84	-0,74	1,90	0,03	0,24	1,20
Żukowo (3)	987	6	52	-0,96	1,50	0,64	3,41	1,23	1,26	-0,50
Dziemiany (2)	305	19	27	0,46	-1,24	-1,48	2,34	-0,18	0,22	2,24
Karsin (2)	496	25	16	0,87	-0,80	-1,08	0,82	0,45	0,29	1,64
Kościerzyna (2)	1739	41	50	-0,42	-1,11	-0,39	2,23	-0,40	0,77	0,73
Liniewo (2)	443	39	39	0,19	-0,51	-0,08	0,42	0,19	-0,06	0,37
Lipusz (2)	235	7	23	0,63	-1,04	-0,98	2,21	0,02	0,22	1,91
Nowa Karczma (2)	539	21	41	0,09	-0,29	0,06	2,28	1,36	-0,58	0,43
Stara Kiszewa (2)	860	40	82	-0,14	-1,01	-0,42	1,31	0,27	-0,08	0,57
Gardeja (2)	604	21	32	0,65	-0,48	0,00	1,14	-0,85	-0,46	-1,22
Kwidzyn (2)	694	40	101	1,60	-0,14	-0,33	1,30	-0,53	0,25	-1,09
Prabuty (3)	602	31	84	1,14	-0,20	-0,52	0,32	-0,06	0,04	-0,80
Ryjewo (2)	546	21	45	0,41	-0,55	-0,52	0,54	0,08	-0,06	-0,93
Sadlinki (2)	572	32	56	0,09	-0,60	-0,28	1,79	-0,59	-1,06	-0,56
Cewice (2)	491	12	30	-0,12	-0,84	-0,19	2,04	-0,20	-0,12	0,07
Nowa Wieś Lęborska (2)	845	28	78	0,63	-0,62	0,06	1,28	-0,11	0,60	0,30
Wicko (2)	366	8	25	0,70	-1,04	1,46	0,76	-0,47	1,65	0,60

Lichnowy (2)	153	14	27	6,48	0,56	-1,34	1,14	-1,01	-1,45	0,12
Malbork (2)	344	41	58	1,70	-0,39	-0,31	1,09	-1,01	-0,22	-1,25
Miłoradz (2)	198	13	26	2,41	-0,18	-0,38	0,25	-0,71	-0,41	-0,91
Nowy Staw (3)	376	23	31	1,44	-0,20	-0,39	-0,22	-0,81	-0,16	-1,49
Stare Pole (2)	224	4	24	2,46	-0,04	-0,55	0,90	-0,50	-0,43	-0,73
Nowy Dwór Gdański (3)	626	43	66	3,11	0,53	-0,75	-0,29	-0,01	-0,19	-0,40
Ostaszewo (2)	353	10	60	0,64	-0,45	-0,36	0,57	-0,84	-0,16	-1,62
Stegna (2)	483	27	40	1,79	-0,49	-0,55	-0,49	-0,46	2,92	-0,68
Sztutowo (2)	229	10	29	0,36	-1,07	-1,36	-0,19	-0,75	3,51	-0,85
Kosakowo (2)	323	3	15	-0,04	1,45	0,11	2,66	-0,24	1,94	-0,06
Krokowa (2)	527	14	43	1,15	-0,24	-0,52	1,48	-0,13	1,04	0,36
Puck (2)	925	31	67	0,36	0,13	-0,53	2,31	-0,02	-0,33	-0,37
Damnica (2)	369	14	27	2,21	-0,25	-0,48	0,88	-1,36	-0,90	-0,38
Dębnica Kaszubska (2)	430	22	38	1,34	-0,75	0,68	0,56	-0,85	0,21	-0,19
Główczyce (2)	821	23	54	1,27	-0,76	0,20	0,57	-0,81	-0,44	-0,05
Kępice (3)	369	6	18	0,55	-0,69	1,26	-0,12	-0,95	0,74	-0,22
Kobylnica (2)	856	27	42	0,59	-0,60	0,21	2,04	-0,73	1,67	-0,64
Potęgowo (2)	626	19	37	1,15	-0,73	0,14	0,93	-0,79	-0,57	-0,01
Słupsk (2)	572	37	45	1,44	-0,30	0,74	1,89	-0,32	0,63	-1,43
Smołdzino (2)	523	13	18	0,23	-1,04	1,09	-0,22	-0,60	0,54	1,13
Ustka (2)	464	16	16	0,37	-1,08	-0,81	0,26	-0,69	2,92	0,15
Bobowo (2)	250	11	17	0,77	-0,29	-0,28	2,10	0,34	-0,50	-0,57
Kaliska (2)	358	0	4	-0,34	-1,11	-0,21	1,20	-0,51	0,53	1,06
Lubichowo (2)	357	18	10	0,03	-0,83	-0,32	0,95	1,01	0,15	0,39
Osieczna (2)	204	55	84	0,42	-0,90	-0,81	0,82	0,38	-0,27	1,98
Osiek (2)	459	1	3	-0,76	-1,37	-0,60	-0,55	-0,37	1,01	1,20
Skarszewy (3)	812	33	43	0,05	-0,62	-0,23	1,80	0,02	0,02	-0,19
Skórcz (2)	422	36	38	1,25	-0,17	-0,50	0,89	0,51	-0,99	-0,78
Smętowo Graniczne (2)	344	17	14	0,86	-0,31	-0,47	0,41	-0,07	-0,55	-0,86
Starogard Gdański (2)	732	44	28	0,08	-0,18	0,23	2,01	0,43	0,02	-0,55
Zblewo (2)	1219	32	19	-0,33	-0,34	-0,27	1,76	-0,30	0,16	-0,32
Gniew (3)	511	18	56	1,18	-0,07	-0,24	0,13	0,28	0,03	-0,33
Morzeszczyn (2)	204	18	31	3,48	-0,02	-0,42	1,03	0,38	-0,62	-0,28

Pelplin (3)	308	21	47	2,52	0,73	-1,01	0,07	1,47	-0,03	-0,63
Subkowy (2)	278	15	47	2,21	0,16	-0,81	1,63	-0,09	-0,60	-1,32
Tczew (2)	559	33	39	1,09	0,18	-0,14	2,20	-0,35	0,24	-1,28
Choczewo (2)	259	8	28	1,74	-0,85	-0,04	0,37	-1,15	0,62	-0,16
Gniewino (2)	390	8	41	0,25	-0,88	-0,04	1,99	-0,87	-0,09	-0,47
Linia (2)	763	20	28	-0,36	-0,70	-0,59	2,87	-0,02	-0,34	0,79
Luzino (2)	509	15	50	-0,05	-0,15	-0,48	3,48	0,22	0,08	0,70
Łęczyce (2)	600	14	50	0,52	-0,75	-0,22	2,02	-0,32	0,22	0,46
Szemud (2)	1076	31	90	-0,23	0,02	-0,40	3,19	0,42	0,86	0,18
Wejherowo (2)	586	20	32	-0,59	-0,19	0,04	3,15	0,57	1,08	0,24
Dzierzgoń (3)	421	44	118	2,65	-0,01	-0,78	0,48	-0,44	0,29	-0,79
Mikołajki Pomorskie (2)	264	12	29	2,35	-0,23	-0,78	0,61	-0,08	-1,17	-0,25
Stary Dzierzgoń (2)	334	44	44	2,52	-0,50	-0,44	0,70	0,26	-0,96	0,21
Stary Targ (2)	377	36	61	2,40	-0,35	-0,81	0,45	-0,01	-0,95	-0,54
Sztum (3)	816	15	36	0,88	0,17	-0,12	-0,15	-0,55	0,50	-1,06
Bobrowniki (2)	1638	0	0	-1,03	1,69	0,66	-1,38	0,03	0,87	-0,43
Mierzęcice (2)	1262	1	0	-0,90	1,84	-0,08	-0,79	-0,15	0,58	0,33
Psary (2)	1620	4	2	-1,32	1,44	0,48	-0,83	0,17	0,96	-0,26
Siewierz (3)	1868	7	4	-1,01	0,33	-0,16	-0,68	-0,13	1,24	0,38
Bestwina (2)	537	5	10	-0,77	2,98	-0,08	0,12	1,26	0,50	-0,91
Buczkowice (2)	1271	0	0	1,03	7,95	-0,05	-1,25	0,21	-0,96	2,74
Czechowice-Dziedzice (3)	667	8	17	0,89	6,60	0,13	-1,22	1,20	-0,54	-0,23
Jasienica (2)	2599	3	32	-0,81	1,68	-0,06	0,95	0,02	0,42	-0,81
Jaworze (2)	224	1	0	-0,46	3,22	0,10	-1,05	0,51	1,70	0,27
Kozy (2)	274	1	7	-0,63	4,25	1,92	-0,45	0,53	0,35	-0,95
Porąbka (2)	1980	1	3	-0,79	2,00	-0,04	-0,04	-0,27	-0,14	0,68
Wilamowice (3)	2275	4	31	-0,46	2,69	-0,29	0,64	-0,35	-0,35	-1,11
Wilkowice (2)	1271	0	0	-0,34	3,29	-0,15	-1,39	0,11	0,95	1,80
Brenna (2)	1299	2	6	-0,28	0,45	-0,77	0,29	-0,58	1,15	2,08
Chybie (2)	432	2	8	-0,32	2,53	-0,05	0,44	-0,03	-0,59	-0,30
Dębowiec (2)	521	10	24	-0,77	1,19	-0,31	1,10	1,36	0,52	-0,57
Goleszów (2)	943	12	56	-0,52	1,56	0,07	-0,56	0,44	0,67	-0,09
Hażlach (2)	678	6	21	-0,59	1,89	0,06	0,68	0,29	-0,41	-0,89

Istebna (2)	1354	0	0	-1,22	-0,20	-0,39	2,00	-0,34	0,08	1,42
Skoczów (3)	973	4	6	-0,02	4,33	0,15	-0,96	0,40	-0,22	0,07
Strumień (3)	1425	7	22	-0,55	1,67	-0,29	0,58	-0,26	-0,31	-1,29
Zebrzydowice (2)	1499	2	6	-0,95	3,52	0,17	-0,39	0,43	-1,01	-0,60
Blachownia (3)	1152	1	0	-1,08	0,17	0,20	-1,29	-0,31	0,66	0,07
Dąbrowa Zielona (2)	749	12	8	-0,96	-0,64	-0,31	-1,90	-0,07	0,24	0,55
Janów (2)	1510	6	5	-1,21	-1,11	-0,29	0,06	-0,63	0,48	0,24
Kamienica Polska (2)	927	2	5	-0,74	0,42	0,08	-0,87	-0,59	0,90	0,55
Kłomnice (2)	1538	5	9	-0,56	0,20	-0,39	-1,12	-0,47	-0,21	-0,20
Koniecpol (3)	1869	13	20	-0,98	-0,71	-0,28	-1,14	-0,15	0,26	0,61
Konopiska (2)	1252	3	0	-0,91	0,63	-0,26	-0,72	-0,57	0,40	0,63
Kruszyna (2)	717	12	14	-0,43	-0,61	-0,45	-0,05	-0,82	-0,41	-0,54
Lelów (2)	984	18	49	-0,83	-0,53	-0,51	-0,98	-0,15	0,04	-0,01
Mstów (2)	1978	10	14	-1,12	-0,10	0,10	-0,16	-0,51	0,19	-0,22
Mykanów (2)	2102	38	78	-0,84	0,19	-0,31	0,10	-0,62	0,85	-0,66
Olsztyn (2)	1439	43	32	-1,36	-0,36	-0,32	0,68	-0,46	0,97	0,22
Poczesna (2)	1107	3	1	-0,74	1,40	0,03	-1,00	-0,30	0,43	0,43
Przyrów (2)	836	14	33	-0,74	-0,41	-0,22	-1,45	-0,22	0,01	0,58
Rędziny (2)	1515	5	9	-0,73	1,54	-0,22	-0,56	-0,33	0,11	-0,60
Starcza (2)	405	0	0	-0,39	2,07	0,64	-0,82	0,17	-1,13	0,81
Gierałtowice (2)	1320	3	25	0,10	2,83	-0,34	0,08	-0,04	-0,22	-1,29
Pilchowice (2)	819	2	10	0,36	0,23	0,33	0,71	-0,60	0,73	-0,58
Rudziniec (2)	471	5	11	-0,15	-0,21	-0,14	-0,35	1,37	0,43	-0,94
Sośnicowice (3)	680	1	14	-0,40	-0,73	-0,33	0,08	-0,25	0,96	-1,11
Toszek (3)	816	20	12	-0,46	-0,23	-0,31	-0,42	-0,25	-0,07	-0,83
Kłobuck (3)	1515	6	17	-0,71	0,47	-0,28	-0,88	-0,18	0,82	-0,51
Krzepice (3)	1009	23	49	-0,82	0,17	-0,38	-0,80	0,31	0,51	-0,30
Lipie (2)	1131	0	0	-1,03	-0,59	-0,43	-0,85	-0,34	0,23	-0,25
Miedźno (2)	1265	7	19	-1,15	-0,76	-0,38	-0,16	-0,42	0,37	-0,09
Opatów (2)	1170	70	110	-0,83	0,08	-0,09	-0,41	-0,05	-0,30	-0,26
Panki (2)	838	6	20	-1,20	-0,42	-0,26	-0,29	-0,10	0,13	-0,03
Popów (2)	1345	12	38	-1,19	-0,85	-0,31	-0,27	-0,33	0,72	-0,10
Przystajń (2)	893	9	35	-1,14	-0,73	-0,27	-0,29	0,05	0,18	-0,12

Wręczyca Wielka (2)	1798	8	10	-1,29	-0,20	-0,05	-0,36	-0,21	0,48	-0,07
Boronów (2)	304	4	0	-0,95	-0,68	-0,06	-0,01	-0,30	0,38	0,28
Ciasna (2)	587	22	85	0,11	-0,31	-0,34	-0,51	0,65	0,02	-0,16
Herby (2)	213	4	1	0,17	0,26	-0,32	-0,38	0,35	0,90	0,37
Kochanowice (2)	406	11	27	-0,10	0,07	-0,25	0,69	0,14	-0,14	-0,58
Koszęcin (2)	925	9	12	-0,77	-0,45	-0,01	0,21	-0,03	0,53	-0,37
Pawonków (2)	483	17	21	0,19	-0,21	-0,54	0,03	0,59	-0,16	-0,25
Woźniki (3)	781	24	20	-0,38	-0,30	-0,41	-0,43	-0,31	0,42	-0,39
Ornontowice (2)	546	0	6	-0,59	2,17	-0,23	0,96	0,20	-0,44	-1,42
Wyry (2)	188	2	3	-0,18	2,22	-0,11	0,92	0,75	0,47	-0,01
Koziegłowy (3)	2831	18	22	-1,43	-0,13	-0,29	-0,67	-0,01	1,32	-0,08
Niegowa (2)	1439	28	41	-1,06	-0,43	-0,58	0,45	-0,27	0,19	-0,01
Poraj (2)	1133	2	0	-0,75	1,41	0,11	-1,74	-0,17	1,22	0,26
Żarki (3)	1576	6	12	-1,01	-0,30	-0,27	-0,40	-0,34	1,29	0,48
Goczałkowice-Zdrój (2)	235	0	9	-0,51	1,17	-0,71	-0,28	-0,39	1,41	-1,02
Kobiór (2)	60	0	3	-0,41	0,18	-0,24	0,03	-0,26	1,62	0,66
Miedźna (2)	1132	2	10	-0,01	2,73	-0,07	2,15	-0,57	-1,22	-0,38
Pawłowice (2)	701	7	15	0,45	2,44	-0,36	1,10	0,37	-0,66	-1,02
Pszczyna (3)	1410	5	54	-0,03	2,42	-0,32	0,16	0,89	1,15	-1,02
Suszec (2)	917	5	29	-0,32	1,11	-0,23	1,72	-0,27	-0,27	-0,50
Kornowac (2)	617	3	4	-0,54	2,01	0,87	0,05	0,30	-1,36	-0,74
Krzanowice (3)	423	18	33	0,56	0,46	-0,62	-0,89	0,08	-0,97	-1,36
Krzyżanowice (2)	307	11	27	1,63	1,58	-0,61	-0,84	0,93	-0,02	-1,09
Kuźnia Raciborska (3)	507	2	7	-0,76	-0,30	-0,10	-0,66	0,20	0,10	-0,19
Nędza (2)	589	3	7	-0,67	-0,14	-0,25	-0,19	-0,32	-0,25	-0,33
Pietrowice Wielkie (2)	342	14	48	1,53	0,45	-0,70	-0,96	-0,03	-0,16	-1,26
Rudnik (2)	347	19	44	1,24	0,79	-0,66	-0,72	0,23	-0,96	-1,22
Czerwionka-Leszczyny (3)	479	9	2	0,07	2,62	0,35	-0,79	2,08	0,26	-0,83
Gaszowice (2)	531	0	0	-0,54	3,76	0,78	-0,12	1,04	-1,45	-0,33
Jejkowice (2)	414	0	0	-0,63	3,42	-0,17	-0,02	0,10	-0,61	0,28
Lyski (2)	885	2	10	-0,68	0,71	0,10	-0,26	-0,02	-0,30	-0,91
Świerklany (2)	1135	0	0	-0,15	4,62	0,01	0,16	0,05	-0,96	-0,87
Ożarowice (2)	778	1	5	-1,14	1,06	-0,12	-0,38	0,11	1,49	0,37

Świerklaniec (2)	56	0	5	1,10	2,09	-0,08	-0,65	-0,40	2,56	0,53
Tworóg (2)	270	5	10	-0,30	-0,33	-0,24	-0,43	0,53	1,32	-0,12
Zbrosławice (2)	473	18	42	2,31	0,84	-0,90	-0,71	0,48	0,67	-0,69
Bojszowy (2)	355	2	2	-0,56	1,00	0,00	0,91	-0,27	-0,03	-0,08
Chełm Śląski (2)	293	0	0	-0,61	2,70	0,16	-0,28	0,11	0,01	-0,01
Godów (2)	583	1	0	-0,19	3,32	-0,14	-0,27	-0,03	-0,59	-0,15
Gorzyce (2)	1002	1	7	-0,59	2,23	-0,36	0,06	0,30	-0,41	-0,48
Lubomia (2)	238	1	9	-0,20	0,83	-0,44	-0,38	0,50	-0,39	-0,89
Marklowice (2)	629	0	1	-0,42	3,58	-0,24	-1,04	-0,17	-1,15	-0,73
Mszana (2)	507	2	4	0,27	3,55	0,29	0,34	-0,02	-1,24	-0,68
Irządze (2)	608	23	90	-0,42	-0,02	-0,27	-2,09	0,29	-0,87	0,00
Kroczyce (2)	1194	9	43	-0,87	-0,90	-0,66	-0,20	-0,56	0,45	0,24
Łazy (3)	1742	3	11	-0,88	0,02	0,30	-1,06	-0,60	1,30	0,32
Ogrodzieniec (3)	1632	4	10	-0,82	-0,16	-1,04	-1,19	-0,63	1,44	0,68
Pilica (3)	1548	48	85	-0,81	-0,70	-0,54	-0,60	0,05	0,26	-0,28
Szczekociny (3)	1075	48	158	-0,59	-0,41	-0,46	-1,47	0,76	0,69	0,33
Włodowice (2)	1104	1	2	-0,90	-0,27	0,20	-1,15	-0,46	0,34	0,73
Żarnowiec (2)	953	111	160	-0,30	-0,72	-0,82	-0,11	0,76	-0,42	0,14
Czernichów (2)	629	0	0	0,62	1,18	-1,55	-0,68	-1,74	1,27	3,68
Gilowice (2)	712	0	0	-0,26	1,92	-0,53	0,16	-0,42	-0,56	2,21
Jeleśnia (2)	2568	0	0	2,71	-0,25	-4,94	-0,43	-3,37	1,06	8,01
Koszarawa (2)	260	0	0	-0,19	0,24	-0,54	-0,18	-1,12	-0,12	2,36
Lipowa (2)	1593	0	0	-0,16	1,18	-0,58	0,41	-1,15	0,13	0,44
Łękawica (2)	602	0	0	-0,61	0,28	0,11	0,65	-1,16	-0,58	1,72
Łodygowice (2)	1030	1	2	-0,12	5,10	-0,45	0,08	0,36	-0,41	1,14
Milówka (2)	1940	1	0	-0,55	-0,02	-0,38	0,22	-1,08	0,04	2,29
Radziechowy-Wieprz (2)	1632	0	0	-0,48	0,85	-0,40	0,36	-0,85	-0,32	1,87
Rajcza (2)	1720	0	0	-0,18	-0,24	-0,85	-0,54	-1,49	0,55	2,48
Ślemień (2)	705	0	0	-0,57	-0,38	-0,84	0,10	-0,95	0,72	2,26
Świnna (2)	1573	0	1	0,58	2,14	-1,11	-0,69	-1,56	-0,39	3,04
Ujsoły (2)	814	0	1	-0,62	-0,62	-0,23	-0,80	-1,10	0,53	2,65
Węgierska Górka (2)	1958	0	3	-0,15	0,77	-0,80	0,30	-1,05	0,24	2,68
Busko-Zdrój (3)	4979	37	57	-1,02	0,58	0,01	-1,41	-0,10	1,06	0,09

Gnojno (2)	1148	18	62	-1,07	-0,51	-0,36	-0,27	-0,14	-0,27	0,45
Nowy Korczyn (2)	1372	36	87	-0,85	0,06	0,46	-1,82	-0,14	-0,72	-0,05
Pacanów (2)	1903	39	85	-1,01	0,13	-0,18	-1,42	-0,32	-0,81	-0,60
Solec-Zdrój (2)	1082	12	37	-0,94	0,20	0,54	-0,78	-0,30	-0,04	0,09
Tuczępy (2)	975	1	31	-0,76	-0,11	-0,07	-1,33	-0,46	-0,19	0,53
Wiślica (2)	1209	29	133	-0,95	-0,39	-0,09	-1,24	-0,23	-0,36	-0,02
Imielno (2)	1024	40	48	-0,91	-0,66	-0,19	-0,10	0,33	-0,55	-0,18
Jędrzejów (3)	2787	54	147	-0,93	-0,06	-0,16	-1,12	0,48	0,60	-0,50
Małogoszcz (3)	1706	11	30	-0,97	-0,59	-0,53	0,03	-0,23	0,18	0,48
Nagłowice (2)	954	27	66	-0,87	-0,78	-0,37	-0,49	0,20	-0,22	-0,36
Oksa (2)	971	23	91	-0,79	-0,56	-0,53	-0,56	0,30	-0,50	0,15
Sędziszów (3)	2020	41	47	-1,01	-0,43	-0,32	-0,65	-0,20	-0,17	-0,48
Słupia (2)	816	48	78	-0,55	-0,74	0,03	-0,25	0,40	-0,58	-0,04
Sobków (2)	1356	15	48	-0,86	-0,51	-0,28	0,05	0,06	-0,09	0,06
Wodzisław (2)	1379	72	115	-0,60	-0,75	-0,61	-0,78	0,12	0,06	-1,00
Bejsce (2)	750	18	29	-0,96	0,03	-0,14	-1,99	-0,09	-0,88	-0,76
Czarnocin (2)	887	38	99	-0,58	-0,11	-0,55	-2,04	-0,45	-0,81	-1,11
Kazimierza Wielka (3)	1999	50	235	-0,70	0,11	-0,51	-1,29	-0,44	-0,49	-1,20
Opatowiec (2)	636	26	76	-0,34	-0,16	-0,35	-2,67	0,00	-0,53	-0,79
Skalbmierz (3)	1271	61	246	-0,79	0,09	-0,57	-1,22	-0,41	-0,79	-1,18
Bieliny (2)	1589	7	6	-1,15	0,05	-0,26	1,01	-0,38	-0,53	0,35
Bodzentyn (3)	2280	4	19	-1,15	-0,39	-0,01	0,09	-0,52	-0,45	0,26
Chęciny (3)	2142	4	8	-1,21	-0,11	-0,02	0,21	-0,30	0,03	0,45
Chmielnik (3)	1560	12	32	-0,99	-0,41	-0,26	-0,22	-0,23	0,04	0,37
Daleszyce (3)	2306	1	6	-1,15	-0,64	-0,31	0,89	-0,68	0,70	0,79
Górno (2)	1941	5	15	-1,19	0,44	-0,30	1,67	-0,34	-0,22	0,37
Łagów (2)	1328	12	23	-1,04	-0,36	0,04	-0,01	-0,15	-0,36	0,13
Łopuszno (2)	1526	20	100	-1,01	-0,73	-0,22	0,43	0,04	0,01	0,72
Masłów (2)	966	2	9	-1,03	-0,14	-0,50	0,72	-0,46	0,98	0,79
Miedziana Góra (2)	1607	0	5	-1,21	0,14	0,21	0,69	-0,46	0,79	0,79
Mniów (2)	1855	1	27	-1,13	-0,22	-0,12	1,03	-0,22	-0,33	0,95
Morawica (2)	1562	7	10	-0,93	0,49	0,10	1,77	-0,24	0,58	0,42
Nowa Słupia (2)	1583	7	18	-1,09	0,39	-0,21	-0,99	-0,21	-0,38	-0,09

Piekoszów (2)	1463	6	7	-0,80	0,51	-0,34	0,67	-0,39	-0,18	0,75
Pierzchnica (2)	966	11	22	-1,01	-0,80	-0,38	0,64	-0,20	-0,34	0,71
Raków (2)	1257	13	40	-0,89	-0,93	-0,52	-0,79	-0,38	0,43	0,83
Sitkówka-Nowiny (2)	415	0	0	-1,02	0,56	-0,24	-0,27	-0,34	0,50	0,77
Strawczyn (2)	1698	10	32	-1,07	0,41	-0,23	1,43	-0,12	-0,37	0,40
Zagnańsk (2)	2084	0	6	-0,64	0,53	-0,32	-0,45	-0,96	0,46	1,29
Fałków (2)	1073	3	5	-1,07	-0,86	-0,42	-0,96	-0,42	-0,25	0,91
Gowarczów (2)	997	4	6	-1,01	-0,66	0,69	-0,38	-0,47	0,06	0,51
Końskie (3)	2820	9	24	-0,69	0,45	0,51	-1,50	-0,35	1,13	0,70
Radoszyce (2)	1504	11	115	-0,23	-0,45	-0,11	-0,20	-0,34	-0,17	1,89
Ruda Maleniecka (2)	817	3	10	-0,93	-0,89	-0,16	-1,29	-0,52	0,04	0,95
Słupia (Konecka) (2)	631	11	95	-0,32	-0,44	-0,38	-1,66	0,04	-0,23	0,81
Smyków (2)	904	0	3	-0,95	-0,52	-0,32	0,02	-0,60	0,02	1,24
Stąporków (3)	2807	2	0	-0,53	-0,45	-0,45	-1,91	-1,13	0,54	1,65
Baćkowice (2)	1026	24	28	-0,54	-0,29	-0,40	-0,77	-0,10	-0,91	-0,61
Iwaniska (2)	1403	15	42	-1,18	-0,27	1,03	0,13	-0,12	-0,92	-0,56
Lipnik (2)	1020	40	86	-0,44	0,37	0,02	-1,07	-0,48	-1,04	-1,58
Opatów (3)	1257	70	110	-0,20	0,49	-0,47	-1,98	-0,34	0,20	-1,63
Ożarów (3)	1674	70	150	-0,33	-0,08	-0,42	-1,18	-0,75	-0,08	-0,89
Sadowie (2)	752	30	78	-0,07	0,06	-0,61	-1,40	-0,21	-0,31	-1,43
Tarłów (2)	1303	31	62	-0,85	-0,63	-0,48	-1,40	-0,38	-0,29	-0,21
Wojciechowice (2)	718	43	99	-0,14	0,49	-0,32	-1,16	-0,51	-0,75	-1,85
Bałtów (2)	748	21	28	-0,87	-0,71	0,61	-1,30	-0,44	-0,11	-0,45
Bodzechów (2)	1968	27	34	-1,10	0,39	0,82	-1,21	-0,38	0,04	-1,05
Ćmielów (3)	653	24	48	0,07	-0,02	-0,19	-1,71	-0,68	-0,10	-0,89
Kunów (3)	1205	7	3	-0,96	0,07	0,01	-0,81	-0,55	-0,13	-0,23
Waśniów (2)	1419	29	72	-0,68	-0,15	-0,30	-0,94	-0,34	-1,21	-1,18
Działoszyce (3)	930	36	57	-0,71	-0,22	-0,28	-2,07	0,16	-0,54	-0,95
Kije (2)	1057	18	59	-0,93	-0,24	1,26	-1,13	-0,20	-0,17	0,19
Michałów (2)	953	42	133	-0,67	-0,32	0,06	-1,26	0,04	-0,52	-0,60
Pińczów (3)	2683	48	157	-0,96	0,11	1,26	-1,33	-0,21	0,04	-0,05
Złota (2)	986	29	88	-0,75	-0,19	-0,39	-1,60	-0,54	-0,34	-0,70
Dwikozy (2)	1744	41	86	-1,06	0,78	-0,48	-1,03	-0,50	-0,62	-1,45

Klimontów (2)	1653	28	47	-1,03	-0,16	-0,33	-0,53	-0,50	-0,75	-0,94
Koprzywnica (3)	1584	16	96	-1,25	0,36	-0,15	-0,99	-0,58	-0,69	-0,71
Łoniów (2)	1530	15	52	-1,12	0,07	-0,16	-0,94	-0,88	-0,50	-0,49
Obrazów (2)	1613	60	216	-1,13	0,82	-0,13	-1,55	-0,58	-0,55	-1,43
Samborzec (2)	2132	50	224	-1,24	0,99	-0,23	-1,33	-0,49	-0,42	-1,50
Wilczyce (2)	821	30	70	-0,72	0,47	-0,41	-0,81	-0,73	-1,00	-1,28
Zawichost (3)	775	34	74	-0,79	0,01	-0,27	-1,21	-0,52	-0,41	-0,94
Bliżyn (2)	1627	1	4	-0,76	-0,34	-0,08	-1,37	-0,88	0,30	1,24
Łączna (2)	1056	2	3	-1,02	-0,21	-0,14	-0,47	-0,52	-0,38	1,03
Skarżysko Kościelne (2)	1200	4	0	-1,19	0,26	-0,05	-0,77	-0,49	0,10	0,39
Suchedniów (3)	931	0	0	-0,88	0,19	-0,14	-1,96	-0,59	1,33	0,61
Brody (2)	1963	1	4	-1,18	-0,06	0,13	-0,62	-0,57	0,02	0,24
Mirzec (2)	1704	7	15	-1,36	0,12	1,50	-0,71	-0,05	-0,31	-0,09
Pawłów (2)	2727	18	25	-1,08	0,72	0,02	0,12	-0,40	-0,96	-0,42
Wąchock (3)	338	1	2	-0,62	0,33	0,33	-1,72	-0,59	0,73	0,81
Bogoria (2)	1683	36	24	-1,18	-0,27	0,26	-0,29	-0,35	-0,40	-0,33
Łubnice (2)	1053	37	60	-1,19	-0,17	1,18	-1,87	-0,06	-0,78	-0,61
Oleśnica (2)	880	8	6	-1,05	-0,10	0,19	-0,17	-0,31	-0,15	0,49
Osiek (3)	1887	11	22	-1,41	-0,65	0,33	0,18	-0,38	-0,60	0,14
Połaniec (3)	1042	10	15	-0,96	0,35	-0,07	0,50	-0,34	-0,40	0,32
Rytwiany (2)	1252	6	12	-1,09	-0,52	-0,21	-0,44	-0,49	0,39	0,59
Staszów (3)	2995	27	24	-0,97	0,20	0,29	-1,08	-0,30	0,75	0,19
Szydłów (2)	1089	51	149	-1,09	-0,77	-0,22	-0,51	-0,35	-0,63	-0,18
Kluczewsko (2)	955	22	40	-0,66	-0,73	-0,50	-0,33	0,08	0,33	1,02
Krasocin (2)	1704	13	37	-0,83	-0,72	-0,51	-0,04	-0,20	1,02	1,03
Moskorzew (2)	537	18	74	-0,79	-0,72	-0,31	-1,40	0,14	-0,22	-0,21
Radków (2)	534	22	66	-0,66	-0,40	0,93	-2,37	0,08	-0,18	0,30
Secemin (2)	866	20	62	-0,76	-0,89	-0,68	-0,74	0,00	-0,26	0,86
Włoszczowa (3)	2010	36	123	-0,76	-0,44	0,17	-0,88	0,08	0,35	0,45
Bartoszyce (2)	990	70	88	2,18	-0,03	1,10	0,18	-0,22	-1,29	-0,22
Bisztynek (3)	562	34	53	2,85	-0,18	-0,18	-0,69	0,15	-0,76	-0,39
Górowo Iławeckie (2)	941	23	29	0,87	-0,28	2,33	0,23	0,21	-1,88	0,69
Sępopol (3)	681	32	42	1,70	-0,62	0,03	-0,69	-0,60	-0,63	-0,58

Braniewo (2)	588	50	52	1,68	-0,12	2,47	0,63	-0,34	-1,77	-0,28
Frombork (3)	203	3	10	0,05	-0,67	1,59	-1,50	-0,75	0,19	-0,13
Lelkowo (2)	314	25	16	3,40	0,54	1,97	-1,09	-0,15	-1,91	-0,10
Pieniężno (3)	541	46	58	1,63	-0,32	1,81	-0,84	0,62	-0,77	0,40
Płoskinia (2)	298	28	30	2,10	0,36	1,67	0,11	-0,09	-1,46	-0,17
Wilczęta (2)	400	41	28	1,34	0,66	2,10	0,00	0,85	-2,40	0,31
Działdowo (2)	958	47	87	1,44	0,01	0,65	1,18	0,26	-1,31	0,17
Iłowo-Osada (2)	844	15	11	0,12	-0,31	-0,14	0,54	-0,47	-0,52	-0,44
Lidzbark (3)	856	43	77	0,57	-0,34	-0,10	-0,04	0,50	-0,20	0,44
Płośnica (2)	817	37	76	0,58	-0,33	-0,01	-0,40	0,85	-1,01	0,72
Rybno (2)	613	32	27	-0,07	-0,56	0,95	0,76	0,26	-0,57	0,00
Elbląg (2)	728	42	44	0,93	0,35	1,79	0,74	-0,28	-0,58	-0,93
Godkowo (2)	525	28	9	1,63	0,39	1,59	-0,73	0,40	-1,88	0,10
Gronowo Elbląskie (2)	523	18	17	0,74	-0,53	-0,26	0,49	-0,85	-0,49	-0,91
Markusy (2)	577	24	28	1,52	1,09	0,87	0,36	0,74	-2,39	0,28
Milejewo (2)	459	8	7	0,65	0,87	2,08	1,07	0,39	-1,76	0,55
Młynary (3)	539	19	17	0,34	-0,55	1,13	0,30	0,58	-0,71	0,86
Pasłęk (3)	698	56	49	1,83	-0,19	-0,34	-0,08	0,55	-0,46	0,39
Rychliki (2)	280	15	19	2,46	0,53	0,24	-0,12	-0,20	-1,69	-0,55
Tolkmicko (3)	417	2	0	0,08	-0,80	0,62	0,05	-0,99	0,89	-0,10
Ełk (2)	1000	49	38	0,73	-0,20	2,58	1,02	0,04	-0,67	0,34
Kalinowo (2)	861	28	53	1,39	0,28	1,46	-0,62	0,40	-2,11	0,27
Prostki (2)	666	22	48	0,94	-0,47	1,31	0,61	0,61	-1,24	0,73
Stare Juchy (2)	543	14	19	0,73	0,25	3,11	-0,99	0,47	-1,70	1,07
Giżycko (2)	889	22	33	0,92	0,01	0,89	0,05	1,19	0,68	0,25
Kruklanki (2)	508	18	20	0,12	-0,12	1,56	0,17	-0,07	-0,30	0,30
Miłki (2)	559	20	24	1,09	0,36	2,06	-0,44	0,18	-1,49	0,24
Ryn (3)	358	16	41	2,36	0,07	1,64	-0,47	0,65	0,13	0,61
Wydminy (2)	859	29	61	0,43	-0,35	2,95	-0,21	0,59	-0,62	0,54
Iława (2)	1187	40	116	0,30	-0,46	-0,05	1,11	1,55	-0,19	0,21
Kisielice (3)	608	26	63	1,14	-0,67	-0,67	0,88	-0,11	-0,51	-0,91
Lubawa (2)	1389	114	120	0,44	0,26	0,17	1,07	2,12	-1,43	-0,25

Susz (3)	561	17	41	1,32	-0,15	-0,10	0,46	1,19	-0,32	0,09
Zalewo (3)	741	23	28	0,84	-0,59	0,00	-0,19	-0,15	-0,57	0,13
Barciany (2)	577	25	29	3,93	-0,19	-1,06	-0,46	-0,10	-1,48	-0,12
Kętrzyn (2)	509	28	46	2,43	0,07	1,02	0,24	-0,41	-1,12	-0,17
Korsze (3)	442	23	15	2,04	-0,21	0,23	-0,21	-0,72	-0,76	-0,11
Reszel (3)	459	14	22	1,85	0,01	0,25	-1,48	-0,07	0,29	0,40
Srokowo (2)	573	15	25	1,96	0,40	0,54	-0,61	-0,15	-1,73	-0,30
Kiwity (2)	622	30	29	1,07	0,64	1,38	-0,17	1,03	-2,12	0,38
Lidzbark Warmiński (2)	1007	45	64	0,77	-0,65	0,72	0,86	0,19	-0,93	0,35
Lubomino (2)	444	27	35	1,69	0,81	2,75	0,43	0,87	-1,89	0,10
Orneta (3)	513	36	37	1,14	-0,40	1,81	-0,89	0,30	0,52	0,08
Mikołajki (3)	458	7	21	0,99	-0,38	1,78	-0,96	0,14	1,54	0,84
Mrągowo (2)	1052	29	43	1,11	-0,02	1,40	0,78	0,78	-0,58	0,46
Piecki (2)	1007	17	30	0,29	-0,96	-0,08	0,53	0,22	0,23	0,93
Sorkwity (2)	621	6	11	1,18	0,32	1,38	0,15	0,52	-1,00	1,39
Janowiec Kościelny (2)	527	65	137	1,90	0,74	0,78	-0,49	2,00	-2,14	1,30
Janowo (2)	385	20	50	1,01	0,07	0,19	-0,49	0,94	-1,00	1,60
Kozłowo (2)	502	18	36	2,13	-0,69	0,25	1,07	-0,43	-1,02	0,48
Nidzica (3)	664	13	37	0,87	-0,27	0,73	-0,21	-0,36	0,51	-0,03
Biskupiec (2)	830	81	144	0,74	-0,61	-0,61	0,86	1,33	-0,50	-0,51
Grodziczno (2)	715	59	90	0,91	0,68	0,20	0,53	2,27	-1,69	0,17
Kurzętnik (2)	915	48	84	0,07	0,69	0,68	0,45	1,70	-1,30	-0,33
Nowe Miasto Lubawskie (2)	852	45	74	0,68	0,81	0,50	0,87	1,30	-1,15	-0,53
Kowale Oleckie (2)	748	29	31	2,00	-0,60	1,32	0,13	-0,05	-0,56	0,98
Olecko (3)	1041	36	40	0,88	0,13	1,03	0,22	0,33	0,66	-0,16
Świętajno (2)	428	17	19	1,31	-0,83	-0,31	0,81	0,78	-0,53	1,02
Wieliczki (2)	553	25	61	0,70	-0,64	0,70	0,76	0,74	-1,00	0,67
Barczewo (3)	1145	12	36	-0,21	-0,59	1,15	0,74	-0,01	0,43	-0,04
Biskupiec (3)	758	81	144	0,77	0,12	1,86	-0,37	1,00	-0,23	0,99
Dobre Miasto (3)	581	49	44	0,99	0,19	1,51	-0,47	0,10	-0,28	0,41
Dywity (2)	1171	8	27	-0,24	-0,25	1,13	1,15	-0,66	1,55	-0,39
Gietrzwałd (2)	320	4	20	1,34	-0,43	0,90	0,95	-0,49	1,23	0,01
Jeziorany (3)	435	28	36	1,51	-0,02	1,60	-0,58	0,67	-1,37	0,55

Jonkowo (2)	738	13	23	-0,38	-0,53	4,64	1,85	0,15	0,71	0,18
Kolno (2)	225	14	17	2,65	0,59	0,39	-0,51	1,24	-1,92	0,98
Olsztynek (3)	630	13	12	0,55	-0,56	0,37	0,45	-0,84	0,57	0,47
Purda (2)	565	13	27	0,30	-0,49	1,34	1,08	-0,07	0,06	0,51
Stawiguda (2)	429	4	4	-0,77	-1,13	1,39	4,01	-0,52	2,75	-0,53
Świątki (2)	505	10	9	1,32	0,42	3,92	0,29	-0,26	-1,78	-0,28
Dąbrówno (2)	342	12	27	1,31	-0,58	0,17	-0,02	0,18	-0,19	0,07
Grunwald (2)	417	23	47	1,67	-0,47	0,95	0,72	0,36	-1,11	0,28
Łukta (2)	386	5	15	-0,64	-0,95	0,47	0,84	-0,48	0,19	0,33
Małdyty (2)	434	23	27	1,83	-0,50	-0,36	-0,01	0,40	-0,57	0,16
Miłakowo (3)	304	12	28	2,34	-0,20	0,74	-0,68	-0,55	-0,41	0,56
Miłomłyn (3)	625	16	42	0,36	-0,89	-0,06	0,16	-0,38	0,15	0,21
Morąg (3)	1247	40	36	0,40	0,02	0,88	-0,16	0,34	-0,01	0,18
Ostróda (2)	933	41	43	1,26	0,04	0,59	0,93	0,71	-0,14	-0,18
Biała Piska (3)	764	72	112	2,18	-0,30	0,20	0,03	1,68	-1,58	2,05
Orzysz (3)	977	19	28	0,34	-0,73	1,53	-0,44	-0,39	-0,42	1,12
Pisz (3)	873	32	100	0,85	-0,28	1,21	0,01	0,84	0,16	1,68
Ruciane-Nida (3)	330	3	11	1,23	-0,49	2,92	-1,41	-0,29	0,86	2,28
Dźwierzuty (2)	514	10	28	1,06	-0,44	0,90	0,18	0,56	-1,14	0,79
Jedwabno (2)	284	5	16	0,54	-0,78	1,86	0,02	-0,09	0,09	1,44
Pasym (3)	302	8	24	0,69	-0,60	0,72	0,36	0,30	0,06	1,26
Rozogi (2)	720	36	41	0,94	-0,58	-0,26	1,18	1,15	-1,21	2,24
Szczytno (2)	618	26	31	0,90	-0,01	1,09	1,68	1,06	-0,56	1,68
Świętajno (2)	494	20	30	0,86	-0,31	3,11	-0,48	1,09	-0,47	1,96
Wielbark (2)	550	18	29	1,03	-0,62	0,73	1,43	1,38	-1,49	2,46
Banie Mazurskie (2)	594	20	14	0,59	-0,26	5,09	-0,92	0,74	-1,02	0,45
Dubeninki (2)	461	3	26	0,73	0,44	5,21	-1,03	1,13	-1,87	0,71
Gołdap (3)	999	30	73	0,74	0,10	7,54	0,26	0,62	-0,92	0,54
Budry (2)	389	22	12	2,27	0,69	1,88	-1,40	1,25	-2,21	0,67
Pozezdrze (2)	494	12	22	0,14	-0,52	1,09	-0,50	0,72	-0,39	0,88
Węgorzewo (3)	636	27	38	1,57	0,16	1,29	-1,29	0,64	0,20	0,21
Budzyń (2)	656	69	115	0,85	-0,33	1,04	0,83	0,63	0,16	-0,18
Chodzież (2)	312	22	39	0,74	-0,62	0,59	0,95	0,71	0,07	0,83

Margonin (3)	371	39	56	1,63	0,39	0,11	0,18	1,79	0,11	0,25
Szamocin (3)	483	21	16	-0,13	-0,31	0,78	0,40	0,21	-0,05	0,41
Czarnków (2)	1214	74	135	0,26	-0,69	0,14	1,10	0,82	-0,05	0,40
Drawsko (2)	420	11	20	0,51	-0,44	-0,29	-0,24	0,20	-0,27	1,75
Krzyż Wielkopolski (3)	348	14	21	-0,95	-0,86	1,81	-0,05	-0,24	0,49	0,61
Lubasz (2)	630	30	74	0,17	-0,71	0,87	1,30	0,25	0,10	-0,20
Połajewo (2)	679	58	119	0,37	-0,61	-0,50	0,86	1,57	-0,55	0,25
Trzcianka (3)	941	27	38	1,32	-0,50	0,49	0,03	-0,53	0,92	1,95
Wieleń (3)	849	28	46	-0,04	-0,94	0,70	-0,01	0,48	0,23	1,01
Czerniejewo (3)	346	29	40	2,80	0,16	-0,76	0,75	1,21	-0,28	0,02
Gniezno (2)	691	61	152	0,85	-0,01	0,11	2,51	0,77	0,62	-0,72
Kiszkowo (2)	427	52	109	1,52	-0,22	-0,58	0,70	0,53	0,14	-0,59
Kłecko (3)	547	72	100	1,15	-0,52	-0,87	1,15	0,58	-0,07	-1,00
Łubowo (2)	448	41	70	1,10	0,31	-0,65	1,83	0,97	0,44	-1,01
Mieleszyn (2)	333	31	44	1,43	-0,29	-0,58	0,83	0,40	-0,25	-0,79
Niechanowo (2)	344	38	56	1,19	-0,17	-0,92	1,42	1,47	-0,42	-1,08
Trzemeszno (3)	750	63	117	0,94	-0,15	-0,78	0,24	0,60	-0,11	-0,50
Witkowo (3)	735	64	103	0,88	-0,32	-0,84	0,35	0,61	0,31	-0,33
Borek Wielkopolski (3)	676	30	97	0,89	0,28	-0,67	-0,10	1,66	-0,55	-0,92
Gostyń (3)	690	53	86	0,64	1,33	-0,54	-0,05	1,75	0,45	-0,42
Krobia (3)	947	61	159	0,80	0,79	-0,84	0,23	1,60	0,13	-1,03
Pępowo (2)	505	57	172	0,29	0,35	-0,86	0,37	2,12	-0,42	-0,86
Piaski (2)	541	95	255	0,73	0,60	-0,81	0,36	1,42	-0,22	-0,73
Pogorzela (3)	539	60	195	1,08	0,26	-0,87	-0,17	1,55	-0,73	-0,85
Poniec (3)	499	46	106	0,82	-0,03	-0,88	0,31	1,61	-0,25	-0,42
Granowo (2)	394	28	50	1,33	0,31	-0,59	1,18	0,55	-0,29	-0,83
Grodzisk Wielkopolski (3)	614	25	62	0,52	0,94	-0,43	0,90	1,69	0,91	-0,70
Kamieniec (2)	529	32	72	1,37	-0,01	-0,78	1,34	1,20	-0,86	-0,93
Rakoniewice (3)	1243	28	65	-0,30	-0,27	-0,23	1,41	0,52	0,75	-0,05
Wielichowo (3)	813	37	99	-0,26	-0,26	-0,38	1,09	1,62	0,20	0,09
Jarocin (3)	1312	73	115	0,48	2,03	-0,32	-0,59	0,46	0,29	-0,55
Kotlin (2)	763	25	55	-0,21	0,09	-0,52	1,13	-0,08	-0,61	-1,28
Żerków (3)	849	88	93	0,62	-0,15	-0,79	0,39	1,17	-0,28	-0,37

Blizanów (2)	1416	52	229	-0,59	-0,21	-0,52	0,53	0,07	0,25	-1,06
Brzeziny (2)	817	9	77	-0,90	-0,80	-0,31	0,06	0,52	0,33	0,16
Ceków-Kolonia (2)	720	16	38	-0,49	0,01	-0,45	-0,11	0,90	0,12	-0,05
Godziesze Wielkie (2)	1305	25	101	-1,22	-0,45	-0,32	1,02	0,54	0,22	-0,25
Koźminek (2)	1003	41	129	-1,21	-0,54	-0,41	0,66	0,82	0,11	-0,50
Lisków (2)	985	32	63	-0,93	-0,23	-0,42	-0,24	0,52	-0,16	-0,09
Mycielin (2)	916	31	20	-0,92	-0,91	-0,57	0,77	0,18	-0,47	0,15
Opatówek (2)	1568	35	240	-1,00	0,39	-0,33	-0,36	0,72	1,02	-0,39
Stawiszyn (3)	847	22	69	-0,83	-0,38	-0,35	0,03	-0,18	0,16	-0,86
Szczytniki (2)	1284	46	127	-0,88	-0,09	-0,51	0,28	0,49	-0,45	-0,44
Żelazków (2)	1516	48	182	-0,69	0,26	0,22	0,55	0,11	0,22	-1,34
Baranów (2)	619	28	25	-0,24	0,28	-0,52	1,40	0,59	0,63	-0,10
Bralin (2)	689	15	38	-0,54	-0,79	-0,34	0,93	0,11	1,29	-0,62
Kępno (3)	1027	27	75	-0,03	1,10	-0,35	-0,35	1,04	1,22	-0,11
Łęka Opatowska (2)	423	19	32	1,12	-0,18	-1,18	0,72	0,47	0,03	0,22
Perzów (2)	424	13	33	0,35	-0,48	-0,47	0,52	0,70	0,24	-0,29
Rychtal (2)	291	24	32	0,94	-0,62	-0,58	0,25	-0,24	-0,03	-0,71
Trzcinica (2)	441	17	22	0,16	-0,34	-0,57	0,32	0,14	0,08	-0,17
Babiak (2)	1145	50	70	-0,52	-0,27	-0,03	0,09	0,30	-0,64	-0,36
Chodów (2)	413	32	37	1,27	0,26	-0,89	-1,56	0,77	-1,21	-0,74
Dąbie (3)	1236	59	90	-0,57	-0,50	-0,30	-0,69	0,33	-0,28	0,00
Grzegorzew (2)	832	26	56	-0,61	-0,29	-0,40	0,11	0,47	-0,43	-0,24
Kłodawa (3)	1227	67	136	-0,01	0,20	-0,69	-0,52	0,31	-0,37	-1,03
Koło (2)	944	51	121	-0,36	-0,21	-0,36	0,86	0,34	-0,24	-0,54
Kościelec (2)	799	27	45	-0,61	-0,65	-0,22	0,58	0,12	0,16	0,49
Olszówka (2)	790	25	45	-0,37	-0,28	-0,58	-0,58	0,02	-0,54	-1,00
Osiek Mały (2)	791	21	31	-0,82	-0,19	0,73	1,16	-0,09	-0,31	-0,43
Przedecz (3)	648	33	69	-0,17	-0,46	-0,44	-0,76	0,55	-0,66	-0,50
Golina (3)	1242	16	37	-0,82	-0,30	-0,22	0,55	-0,01	0,36	-0,40
Grodziec (2)	949	29	45	-0,87	-0,84	-0,32	0,65	0,16	-0,05	0,01
Kazimierz Biskupi (2)	1081	13	13	-0,92	-0,22	-0,02	1,04	-0,32	0,19	-1,04
Kleczew (3)	727	23	50	-0,11	-0,12	-0,27	0,26	-0,02	-0,40	-1,05
Kramsk (2)	1516	37	113	-0,67	-0,27	-0,21	0,54	0,29	-0,07	0,59

Krzymów (2)	1090	18	27	-0,98	-0,29	-0,28	1,06	-0,08	0,20	0,66
Rychwał (3)	1287	31	52	-0,93	-0,35	-0,20	-0,05	0,37	-0,04	0,01
Rzgów (2)	885	26	74	-0,90	-0,59	-0,35	1,13	0,60	-0,03	0,17
Skulsk (2)	1175	23	50	-1,07	-0,71	-0,13	0,41	0,01	-0,26	-0,61
Sompolno (3)	1314	43	142	-0,76	-0,44	-0,30	0,49	0,09	-0,09	-0,56
Stare Miasto (2)	1521	20	69	-1,11	0,02	-0,29	1,68	0,19	0,97	-0,09
Ślesin (3)	1631	23	78	-0,65	-0,46	-0,38	-0,11	-0,24	0,90	-0,58
Wierzbinek (2)	1280	26	58	-0,36	-0,43	-0,15	0,33	0,20	-1,01	-0,04
Wilczyn (2)	866	18	29	-0,50	-0,42	-0,45	0,56	-0,04	-0,34	-1,13
Czempiń (3)	427	48	73	3,49	0,39	-1,26	-0,08	2,37	0,20	0,07
Kościan (2)	694	83	135	1,49	0,46	-0,56	0,78	1,64	-0,19	0,09
Krzywiń (3)	1159	63	110	-0,15	-0,62	-0,56	0,69	1,14	0,14	-0,25
Śmigiel (3)	1208	91	162	0,35	0,27	-0,61	0,54	1,83	0,33	-0,11
Kobylin (3)	553	61	156	1,24	0,30	-0,77	-0,11	2,03	-0,89	-0,36
Koźmin Wielkopolski (3)	952	83	202	0,45	0,41	-0,91	0,02	2,40	0,00	-1,02
Krotoszyn (3)	1447	174	245	0,60	1,09	-0,47	-0,23	1,67	-0,14	-0,61
Rozdrażew (2)	615	70	102	0,62	0,32	-1,03	1,18	1,98	-1,18	-0,97
Zduny (3)	424	131	385	0,63	0,37	-0,60	0,89	1,31	-0,04	-0,12
Krzemieniewo (2)	497	26	46	1,62	0,90	-1,09	0,21	2,79	-0,47	-0,42
Lipno (2)	520	29	78	0,35	-0,23	-0,52	2,35	0,64	1,08	-0,99
Osieczna (3)	755	55	84	-0,16	-0,47	-0,45	0,45	0,84	0,82	-0,40
Rydzyna (3)	450	34	60	1,23	0,05	-0,79	1,40	1,28	0,77	-0,41
Święciechowa (2)	369	23	32	0,98	-0,29	-0,89	1,45	1,19	0,59	-0,41
Wijewo (2)	454	20	21	-1,34	-0,61	-0,31	0,46	5,89	2,26	0,30
Włoszakowice (2)	905	37	76	-0,60	-0,53	-0,54	1,13	0,47	1,90	0,03
Chrzypsko Wielkie (2)	311	26	43	0,53	-0,67	-0,58	0,19	0,90	-0,06	-0,49
Kwilcz (2)	330	20	34	0,60	-0,78	0,21	1,22	0,75	0,37	-0,47
Międzychód (3)	558	44	109	1,39	-0,06	0,75	-0,25	0,86	1,23	0,20
Sieraków (3)	344	28	41	0,75	-0,73	-0,71	0,15	1,78	0,98	-0,03
Kuślin (2)	539	22	36	0,66	-0,32	-0,40	0,86	0,12	0,13	-0,81
Lwówek (3)	624	26	50	0,32	-0,46	-0,22	0,34	0,36	0,22	-0,35
Miedzichowo (2)	510	10	21	-0,71	-1,24	0,29	0,27	-0,16	0,95	0,43
Nowy Tomyśl (3)	1325	21	49	-0,74	0,32	-0,07	0,91	0,53	1,57	-0,05

Opalenica (3)	727	46	101	0,60	0,35	-0,71	0,60	0,55	1,03	-0,91
Zbąszyń (3)	803	21	65	-0,21	-0,22	-0,14	0,46	-0,09	1,01	0,20
Oborniki (3)	906	51	111	1,04	0,25	-0,52	0,69	0,86	1,26	-0,63
Rogoźno (3)	510	43	75	1,64	0,18	-0,34	0,58	0,46	0,45	-0,44
Ryczywół (2)	444	78	133	1,47	-0,38	-0,67	0,44	1,59	-0,69	0,32
Nowe Skalmierzyce (3)	1292	70	295	-0,22	0,33	-0,53	0,63	0,06	-0,35	-0,34
Odolanów (3)	1600	43	81	-0,59	0,57	-0,11	0,83	0,38	-0,08	0,23
Ostrów Wielkopolski (2)	1775	75	132	-0,59	0,11	1,59	0,58	0,71	0,24	-1,11
Przygodzice (2)	1207	32	79	-0,33	0,04	-0,30	0,34	0,80	0,66	0,09
Raszków (3)	1179	54	115	-0,15	-0,17	-0,66	0,76	0,82	0,18	-0,90
Sieroszewice (2)	1001	59	120	-0,41	-0,56	-0,67	0,94	1,16	0,01	0,15
Sośnie (2)	618	25	24	-0,29	-0,86	-0,19	0,93	-0,03	0,03	0,54
Czajków (2)	471	14	30	-0,64	-1,07	-1,16	0,66	0,34	0,59	0,85
Doruchów (2)	581	45	52	-0,41	-0,56	-0,64	1,06	0,98	-0,08	0,00
Grabów nad Prosną (3)	898	48	179	-0,43	-0,43	-0,76	0,45	1,35	0,56	0,18
Kobyla Góra (2)	742	11	22	-0,69	-0,78	-0,55	0,74	0,43	0,72	0,35
Kraszewice (2)	666	21	46	-0,54	-0,73	-0,93	0,52	0,14	0,33	0,60
Mikstat (3)	825	23	69	-0,77	0,00	-0,39	0,23	1,52	0,29	-0,09
Ostrzeszów (3)	1289	30	58	-0,61	0,26	-0,37	-0,16	0,68	1,25	0,13
Białośliwie (2)	596	31	67	-0,10	-0,32	-0,23	0,77	0,13	-0,65	-0,10
Kaczory (2)	377	21	43	1,99	0,09	-0,33	0,70	0,84	-0,36	0,55
Łobżenica (3)	888	80	87	0,81	-0,16	-0,43	0,48	1,49	-0,97	-0,28
Miasteczko Krajeńskie (2)	369	21	38	0,30	-0,19	-0,07	0,21	0,11	-0,45	0,56
Szydłowo (2)	472	23	57	2,14	-0,78	0,38	1,97	-1,43	0,75	-1,09
Ujście (3)	454	21	49	1,24	0,07	0,20	-0,60	0,05	0,29	0,14
Wyrzysk (3)	733	68	63	0,99	-0,03	-0,37	0,37	0,99	-0,74	-0,10
Wysoka (3)	476	39	64	1,57	0,09	-0,56	0,52	1,32	-1,19	-0,03
Czermin (2)	700	46	99	-0,09	-0,62	-0,57	1,35	0,47	-0,27	-0,69
Gizałki (2)	677	22	40	-0,33	-0,64	-0,55	0,65	0,12	0,22	0,24
Gołuchów (2)	1030	69	146	-0,04	-0,07	-0,51	0,60	0,09	0,45	-0,72
Pleszew (3)	1323	69	200	0,10	0,53	-0,45	-0,50	0,38	0,76	-0,72
Buk (3)	527	51	79	1,03	0,48	-0,61	0,60	1,06	1,81	-1,07
Czerwonak (2)	179	5	6	-0,86	2,75	0,98	-0,32	7,70	3,00	-0,22

Dopiewo (2)	320	11	40	1,84	1,47	-0,19	3,59	1,26	2,43	-1,16
Kleszczewo (2)	286	45	101	3,54	0,94	-1,22	3,68	1,09	0,62	-0,75
Komorniki (2)	466	11	39	0,36	2,13	0,56	4,39	0,04	2,39	-2,07
Kostrzyn (3)	422	33	101	2,28	0,89	-0,89	0,87	1,02	0,73	-0,35
Kórnik (3)	587	30	68	0,43	0,38	0,21	3,10	0,53	1,94	-0,90
Mosina (3)	794	30	69	0,33	0,83	-0,18	1,23	1,71	2,00	0,04
Murowana Goślina (3)	333	16	29	0,83	-0,14	-0,29	1,15	0,54	1,70	-0,25
Pobiedziska (3)	416	34	100	1,61	0,56	-0,22	1,02	0,56	1,56	-0,34
Rokietnica (2)	292	8	25	0,59	0,97	0,48	3,16	0,20	2,19	-1,67
Stęszew (3)	531	34	61	0,92	0,46	-0,31	0,36	2,80	1,78	-0,71
Suchy Las (2)	289	10	7	-0,47	0,47	0,97	1,19	0,75	4,47	-0,81
Swarzędz (3)	370	20	55	1,95	3,48	0,26	0,84	1,16	1,82	-0,75
Tarnowo Podgórne (2)	811	17	44	-0,11	1,99	0,35	1,97	0,44	3,71	-1,62
Bojanowo (3)	363	29	36	1,85	0,10	-0,88	0,03	1,12	-0,64	-0,06
Jutrosin (3)	782	51	146	0,09	-0,07	-0,71	0,66	2,91	0,00	-0,02
Miejska Górka (3)	844	88	163	0,33	0,33	-0,73	0,44	1,64	-0,58	-0,76
Pakosław (2)	622	57	101	0,42	0,27	-0,57	0,26	1,25	-0,07	-0,02
Rawicz (3)	927	49	107	-0,96	1,62	-0,10	-1,11	8,85	1,92	0,05
Lądek (2)	924	28	67	-0,46	-0,64	-0,23	-0,01	0,09	0,02	-0,26
Orchowo (2)	359	27	58	1,18	-0,23	-0,69	-0,27	-0,12	-0,22	-0,67
Ostrowite (2)	707	38	107	-0,17	-0,75	-0,50	0,60	0,06	-0,20	-0,82
Powidz (2)	212	5	16	0,11	-0,85	-0,38	-0,24	-0,39	1,36	-0,48
Słupca (2)	1526	39	103	-0,54	-0,50	-0,55	0,97	0,14	0,31	-1,12
Strzałkowo (2)	688	46	116	0,74	-0,55	-0,76	0,93	0,40	0,02	-1,06
Zagórów (3)	1187	56	115	-0,59	-0,77	-0,32	0,33	0,52	-0,01	0,50
Duszniki (2)	781	67	136	0,60	0,01	-0,50	1,08	1,23	0,10	-0,91
Kaźmierz (2)	276	21	46	2,34	0,12	-0,85	1,42	0,42	0,55	-0,95
Obrzycko (2)	278	18	13	0,21	-0,64	-0,54	1,00	0,56	0,23	-1,11
Ostroróg (3)	254	9	54	0,38	-0,28	-0,55	0,16	4,40	0,37	-1,15
Pniewy (3)	390	41	247	1,23	0,03	-0,77	0,64	1,60	0,76	-0,77
Szamotuły (3)	1069	36	127	0,23	0,68	-0,48	0,05	1,73	1,16	-1,07
Wronki (3)	1221	30	64	-0,34	-0,73	-0,52	0,17	0,02	0,50	-0,78
Dominowo (2)	277	37	88	1,34	-0,10	-0,86	0,94	2,02	-0,15	-0,24

Krzykosy (2)	561	63	123	0,02	-0,41	-0,59	1,40	1,04	0,16	0,12
Nowe Miasto nad Wartą (2)	660	43	32	-0,35	0,42	-0,47	0,17	6,01	0,17	-0,58
Środa Wielkopolska (3)	634	58	162	1,41	1,16	-0,65	0,26	3,05	1,09	-0,43
Zaniemyśl (2)	300	43	62	0,30	0,49	-0,44	0,40	5,04	0,93	-0,20
Brodnica (2)	198	19	43	4,84	0,92	-1,68	0,33	5,67	-0,77	1,23
Dolsk (3)	709	29	72	-0,08	-0,69	-0,07	1,17	0,74	0,13	-0,80
Książ Wielkopolski (3)	682	38	105	0,25	-0,20	-0,45	0,91	2,22	0,05	-0,04
Śrem (3)	996	48	125	0,87	1,45	-0,05	0,01	1,14	0,89	-0,47
Brudzew (2)	866	15	22	-0,91	-0,68	-0,12	0,57	0,39	-0,47	-0,06
Dobra (3)	824	20	49	-0,46	-0,77	-0,43	-0,30	0,49	-0,33	0,53
Kawęczyn (2)	863	43	70	-0,37	-0,29	-0,44	0,10	0,69	-0,56	0,25
Malanów (2)	863	10	48	-0,66	-0,44	-0,43	0,91	0,26	-0,39	0,35
Przykona (2)	737	20	46	-0,71	-0,55	0,19	0,81	0,23	-0,29	0,07
Tuliszków (3)	1217	12	13	-0,76	-0,77	-0,32	0,75	-0,09	-0,37	0,47
Turek (2)	1399	26	53	-1,31	-0,52	-0,07	2,20	0,00	0,09	-0,25
Władysławów (2)	1101	8	31	-1,11	-0,44	-0,26	1,36	-0,02	-0,35	0,30
Damasławek (2)	422	53	80	1,88	-0,15	-1,02	-0,28	1,39	-0,39	-0,87
Gołańcz (3)	597	107	146	1,75	-0,29	-0,91	1,01	1,56	-0,45	-0,51
Mieścisko (2)	382	34	63	1,76	-0,54	-0,55	0,31	0,55	-0,59	-0,13
Skoki (3)	417	17	59	0,59	-0,69	-0,56	1,81	0,58	0,29	-0,25
Wapno (2)	233	20	33	1,38	0,36	-0,55	-0,61	0,67	-0,95	-0,63
Wągrowiec (2)	1145	157	180	0,91	-0,72	-0,53	1,47	1,05	-0,39	-0,38
Przemęt (2)	1559	65	138	-0,41	-0,62	-0,52	0,89	0,77	1,04	0,33
Siedlec (2)	1415	59	194	-0,10	0,09	-0,33	1,17	1,48	0,70	-0,16
Wolsztyn (3)	1612	24	75	-0,96	0,47	-0,10	0,08	3,46	2,20	0,03
Kołaczkowo (2)	562	73	175	1,33	-0,19	-0,90	1,06	1,73	-0,12	-0,58
Miłosław (3)	454	48	107	3,39	0,06	-1,06	0,10	0,02	0,63	1,68
Nekla (3)	308	18	94	1,08	0,03	-0,77	0,94	0,57	0,83	-0,11
Pyzdry (3)	998	24	102	-0,67	-0,72	-0,27	0,17	0,74	0,27	0,50
Września (3)	904	90	134	1,53	1,06	-0,85	-0,23	1,03	1,02	-0,88
Jastrowie (3)	499	9	23	0,82	-0,86	0,74	0,54	-0,73	0,24	-0,31
Krajenka (3)	576	46	73	0,83	-0,48	0,01	0,89	-0,34	-0,26	-0,46
Lipka (2)	453	19	53	1,69	-0,67	-0,27	0,52	-0,28	-0,45	-0,29

Okonek (3)	389	14	38	1,72	-0,70	0,31	-0,03	-0,77	-0,35	-0,63
Tarnówka (2)	249	13	34	1,26	-0,84	-0,32	0,33	-0,90	-0,27	-0,68
Zakrzewo (2)	500	38	27	0,60	-0,79	-0,50	1,30	0,64	-0,49	0,30
Złotów (2)	801	51	77	1,51	-0,59	0,14	2,09	0,43	-0,98	0,03
Białogard (2)	949	21	59	0,99	-0,63	2,63	0,46	-0,93	-0,25	-0,42
Karlino (3)	299	8	33	1,87	-0,05	0,81	0,33	-0,70	0,44	-0,53
Tychowo (3)	412	10	38	1,35	-0,71	2,88	0,11	-1,00	-0,34	-0,18
Bierzwnik (2)	545	17	27	0,34	-0,69	1,61	-0,67	-0,45	0,52	-0,31
Choszczno (3)	926	40	88	1,34	0,07	0,97	-0,65	-0,89	0,80	-1,32
Drawno (3)	174	15	21	1,98	-0,32	4,59	-0,86	-0,81	-0,29	-0,08
Krzęcin (2)	457	17	46	1,11	-0,53	0,54	0,41	-0,99	-0,51	-1,23
Pełczyce (3)	402	30	85	4,28	0,05	-0,73	0,05	-1,48	0,15	-0,85
Recz (3)	312	6	28	2,15	-0,34	1,79	0,15	-0,49	0,58	-0,62
Czaplinek (3)	819	20	42	0,43	-0,60	3,71	0,08	-0,67	0,74	-0,33
Drawsko Pomorskie (3)	537	37	31	3,10	0,31	5,44	-1,13	-1,06	0,89	0,35
Kalisz Pomorski (3)	270	9	18	0,04	-0,88	3,57	0,58	-0,20	0,56	-0,23
Ostrowice (2)	370	19	14	-0,15	-0,11	9,16	-0,55	-0,11	-0,58	-0,50
Wierzchowo (2)	551	6	21	-0,97	-0,53	4,48	1,45	5,67	-0,47	-2,26
Złocieniec (3)	363	5	16	0,98	0,26	4,41	-0,44	-0,57	0,65	0,01
Goleniów (3)	750	12	37	0,42	0,24	2,12	-0,22	2,41	2,26	0,56
Maszewo (3)	569	28	102	1,36	-0,60	0,17	1,34	-0,38	-0,20	-0,02
Nowogard (3)	806	31	65	2,00	0,12	2,16	-0,43	-0,45	1,22	0,24
Osina (2)	217	4	14	0,98	-0,14	1,59	0,11	0,22	0,15	-0,15
Przybiernów (2)	250	12	29	1,99	-0,37	3,73	-0,10	-0,18	-0,03	0,56
Brojce (2)	211	17	25	1,93	-0,70	-0,08	1,16	-0,50	-0,53	-0,63
Gryfice (3)	473	31	53	3,67	0,40	0,19	-0,56	-0,72	1,74	0,09
Karnice (2)	335	6	26	1,62	-0,64	0,27	0,15	-0,62	0,71	-0,33
Płoty (3)	348	29	53	2,16	-0,57	0,61	0,33	-0,45	0,27	0,12
Rewal (2)	87	3	1	2,84	-0,06	-3,09	-4,36	-0,31	16,65	2,95
Trzebiatów (3)	411	19	53	2,26	0,00	0,02	-0,60	-0,41	1,56	0,09
Banie (2)	451	17	57	3,29	-0,80	-0,30	0,10	-2,22	0,66	-1,16
Cedynia (3)	328	5	20	1,28	-0,96	-0,30	-0,34	-1,31	1,09	-1,19
Chojna (3)	1090	9	36	1,29	-1,00	-0,05	0,25	-1,57	1,27	-1,06

Gryfino (3)	593	19	45	1,56	0,37	0,03	-0,67	0,04	2,02	-0,87
Mieszkowice (3)	361	8	25	1,45	-0,43	0,68	0,29	-0,85	0,37	-0,82
Moryń (3)	173	6	16	3,67	-0,56	-0,56	-0,13	-1,08	0,25	-0,32
Stare Czarnowo (2)	183	3	12	5,05	0,03	-1,01	-0,83	0,17	1,03	0,51
Trzcińsko-Zdrój (3)	408	14	51	1,41	-0,62	-0,21	-0,89	-1,20	0,26	-0,98
Widuchowa (2)	338	9	13	1,56	-0,91	-0,24	0,10	-1,24	0,61	-0,89
Golczewo (3)	329	15	32	1,52	-0,57	1,41	-0,59	0,94	1,21	-0,78
Kamień Pomorski (3)	409	13	41	2,00	-0,18	0,06	-1,48	-0,43	2,25	-0,07
Świerzno (2)	413	7	16	1,51	-0,42	1,40	0,38	-0,44	0,37	0,86
Wolin (3)	470	17	51	1,99	-0,37	0,33	-0,40	0,11	1,74	0,26
Dygowo (2)	420	9	53	1,69	0,18	0,00	0,25	-0,63	0,55	-0,73
Kołobrzeg (2)	575	20	44	0,30	0,11	3,86	0,08	-0,09	3,88	-0,26
Rymań (2)	346	22	61	1,17	-0,47	0,56	0,61	-0,38	0,38	-0,37
Siemyśl (2)	394	15	18	1,41	-0,15	0,64	0,87	-1,01	0,46	-0,98
Ustronie Morskie (2)	211	1	4	0,74	-0,89	-1,52	-1,11	-0,09	9,30	0,23
Będzino (2)	477	17	25	1,64	-0,23	-0,01	0,16	-0,23	0,72	-0,58
Biesiekierz (2)	313	20	19	1,92	0,56	0,07	0,90	-0,32	1,05	-0,42
Bobolice (3)	381	13	51	1,14	-0,29	5,02	-0,36	-0,11	-0,59	0,01
Manowo (2)	351	1	8	0,37	-0,72	1,54	0,78	-0,55	1,32	0,23
Mielno (2)	212	3	9	2,00	-1,33	-3,00	-4,13	-0,06	14,57	2,53
Polanów (3)	476	14	24	1,44	-0,51	4,64	-0,18	-0,57	-0,26	0,29
Sianów (3)	870	22	47	0,47	-0,51	2,01	0,47	-0,39	1,13	-0,16
Świeszyno (2)	506	3	5	0,19	-0,38	1,08	1,72	-1,04	1,39	-0,70
Barlinek (3)	424	13	23	1,59	0,03	0,86	-0,57	-0,90	1,03	-0,94
Boleszkowice (2)	241	9	30	1,20	-0,94	-0,44	0,75	-0,94	0,39	-0,67
Dębno (3)	563	19	79	1,16	-0,02	0,28	-0,12	-0,61	1,32	-0,75
Myślibórz (3)	636	31	85	2,29	-0,40	-0,01	-0,62	-0,80	1,05	-0,65
Nowogródek Pomorski (2)	264	8	13	0,46	-0,82	0,82	0,98	-0,28	-0,01	-0,28
Dobra (Szczecińska) (2)	216	4	9	1,51	1,32	2,03	2,33	-0,48	3,72	0,01
Kołbaskowo (2)	301	7	7	0,78	-0,09	1,01	3,06	-1,37	2,01	-1,43
Nowe Warpno (3)	92	0	4	0,35	-0,89	0,18	-0,88	0,32	1,99	2,25
Police (3)	1158	3	10	-0,39	0,58	1,28	-0,21	-0,27	1,23	0,56
Bielice (2)	324	9	24	1,26	-0,66	0,02	1,03	-1,06	-0,08	-1,00

Kozielice (2)	302	6	18	1,87	-0,69	0,29	0,33	-1,58	0,54	-1,00
Lipiany (3)	296	5	20	0,97	-0,50	0,15	-0,89	-0,95	1,16	-1,12
Przelewice (2)	413	20	67	2,82	-0,30	-0,74	0,29	-1,42	0,21	-1,22
Pyrzyce (3)	1052	44	144	1,12	0,30	0,04	-0,82	-1,10	0,98	-1,39
Warnice (2)	371	13	37	1,45	-0,28	-0,64	-0,20	-1,45	-0,46	-1,80
Darłowo (2)	853	29	41	0,68	-0,91	-0,51	0,51	-0,25	1,89	0,31
Malechowo (2)	485	11	34	2,35	-0,62	0,58	0,25	-0,84	0,48	-0,32
Postomino (2)	593	14	15	0,75	-0,85	0,34	0,10	-0,48	1,12	0,10
Sławno (2)	1088	16	48	-0,20	-0,84	1,26	0,74	-0,42	0,05	-0,46
Chociwel (3)	417	16	26	0,81	-0,87	0,64	-0,46	-0,79	0,34	-0,36
Dobrzany (3)	515	15	17	0,45	-0,84	1,61	-0,42	-0,67	0,72	0,18
Dolice (2)	229	14	34	4,05	-0,02	-0,93	0,15	-1,56	0,13	-0,88
Ińsko (3)	177	6	11	1,21	-0,66	1,74	-1,59	-0,50	0,64	0,83
Kobylanka (2)	259	8	35	-4,76	0,52	0,94	-0,83	18,12	6,59	-0,98
Marianowo (2)	234	8	23	1,18	-0,64	2,60	1,16	-0,69	-0,32	-0,39
Stara Dąbrowa (2)	180	10	43	4,31	-0,50	-0,22	1,55	-1,49	-0,63	-0,51
Stargard (2)	764	0	0	2,77	-0,17	-0,56	1,31	1,87	0,38	-0,71
Suchań (3)	623	22	27	0,45	-0,85	0,38	0,27	-0,80	-0,12	-0,83
Barwice (3)	542	17	32	0,84	-0,24	3,78	0,00	1,34	-0,14	-0,22
Biały Bór (3)	412	17	34	0,58	-0,29	7,25	-0,55	0,02	-0,32	-0,12
Borne Sulinowo (3)	506	4	15	0,95	-0,48	4,56	-1,07	-0,03	0,52	-0,07
Grzmiąca (2)	409	6	14	1,71	-0,31	3,02	-0,92	-0,77	-0,10	-0,32
Szczecinek (2)	647	24	40	0,49	-0,08	10,12	0,82	-0,17	-1,27	-0,62
Brzeżno (2)	306	20	43	1,98	-0,21	1,03	0,56	-0,50	-0,90	-0,19
Połczyn-Zdrój (3)	625	41	50	1,38	0,02	6,60	-1,41	-0,21	0,46	-0,62
Rąbino (2)	299	15	31	1,92	-0,47	2,64	0,05	-0,94	-0,31	-0,98
Sławoborze (2)	275	5	21	1,86	-0,46	0,53	-0,29	-0,71	-0,17	0,16
Świdwin (2)	506	36	55	1,19	-0,27	2,50	0,20	0,98	-0,89	-0,17
Człopa (3)	367	6	13	0,58	-0,93	2,61	-0,02	-0,84	0,30	-0,26
Mirosławiec (3)	169	4	10	2,46	-0,28	3,26	-0,02	-1,16	0,36	0,01
Tuczno (3)	212	18	77	2,32	-0,64	3,24	-0,13	-0,74	0,03	-0,37
Wałcz (2)	1102	41	117	1,02	-0,84	3,19	0,67	-1,28	-0,09	-1,26
Dobra (3)	191	10	30	3,33	-0,44	0,32	-0,01	-1,06	-0,30	0,25

Łobez (3)	363	28	49	1,45	-0,39	-0,12	-0,62	-0,01	1,49	-0,20
Radowo Małe (2)	158	9	46	4,13	-0,43	-0,53	-0,37	-0,75	-0,01	0,63
Resko (3)	269	14	41	1,88	-0,78	-0,39	-0,12	-0,61	0,89	-0,12
Węgorzyno (3)	341	17	32	2,30	-0,83	-0,32	-0,23	-0,87	0,26	0,05
