# Supplementary material for: Spatial determinants of farmers’ interest in European Union’s pro-investment programs in Poland
Source: PLoS One. 2021 Mar 18;16(3):e0248059. doi: 10.1371/journal.pone.0248059 (PMC7971496; doi:10.1371/journal.pone.0248059)
Supplement: Attachment — Submitted filename: Response_to_reviewers.docx [file pone.0248059.s002.docx]

**We thank the reviewer for her/his constructive critiques. We feel the manuscript is greatly improved by incorporating their suggestions. Please find our detailed responses below:**

1. The article has a local dimension, can these results be extrapolated to the international dimension? Could you describe it? The local perspective is interesting, but is it possible for other countries in the EU or in the world to adapt it? How to use this knowledge?

**Thank you for bringing up this point. Although this research concerns Poland, its results contribute to a broader discussion on the impact of local factors on the effects of EU's structural policies. The studies quoted in the paper (lines 452-454) demonstrate that also in other EU countries, in remote and lower income locations where rural development programs are most needed, the programs’ performance is often relatively modest. Thus, it can be concluded that negative selection demonstrated in the paper, is taking place in case of rural development programs not only in Poland but in many EU countries. Moreover, the paper aims to contribute to the discussion on the impact of structural funds on regional differences in EU (lines 72-76 and also 444-454). Our findings suggest that pro-investment programs contribute to the deepening of development differences in Polish agriculture in the territorial dimension, which may be an important hint in for UE policy designing. It has been also indicated that the differentiation of farmers’ interest in the EU support was strongly determined by the agrarian structure. This finding, independent of the specificity of Poland, may constitute a premise for constructing the assumptions of agricultural and structural policy. The discussion of this point has now been added to the conclusions.**

**Finally, in the paper we propose a method of merging the areas which are characterized by a high/low application ratio for selected programs, which may be used for analyzing of other structural programs in only in Poland but in other EU countries.** **Thank you for this remark, it has been now added to the paper (lines 427-429).**

2. Improving the economic situation of farmers also means increasing the standard of living and quality of life. So can you answer: How do investment programs affect farmers' standard of living? The social part of sustainable development is worth pointing out. It will allow us to take a broad look at the problem under investigation.

**Measures ‘modernization of agricultural holdings’ and ‘setting up of young farmers’ were implemented under the 1rd goal of the 2007–2013 RDP to ‘improve the competitiveness of the agricultural and forestry sector.’ Thus, in Polish conditions their main goal was to support the modernization of farms with adequate production potential. In 2007–2013 RDP, these programs were supplemented by the measures of objective 3, which directly aimed at increasing the standard of living and quality of life. However, as farm modernization results in overall better economic situation of farms’ holders, it also positively impacts the standard of living. Therefore, the conclusions of the research were extended to consider the social effects of the implementation of the discussed instruments, indicating that they contribute to the development of agribusiness and the standard of living of farmers mainly in areas where this sector is already strong (lines 463-465). Therefore, the need to apply other, pro-development forms of support in areas with a dysfunction of both agriculture and other sectors of the economy was indicated.**

3. When you look at Figure 1, you can see that two extreme situation - low-low and high-high. This groups dominate. What does that mean? Could you explain it?

**In the paper we used the local Moran’s autocorrelation coefficient as a basis for separating the statistically significant cluster of above-average frequencies of applying for pro-investment support (High-High) from the cluster of below-average values (Low-Low) and outliers. These are the most important area of our investigation. However, we also delimitated the outliers (with a high level of the coefficient of applying for the support but adjacent to municipalities at low levels- High-Low, and with a low level of the coefficient surrounded by municipalities at high levels- Low-High). Our results demonstrate indeed that Low-Low and High-High dominate over outliers. This confirms that the territorial conditions affect the application ratios (lines 238-239). It is especially evident in Poland where agriculture structures differ regionally. However, the existence of the outliers (even if their number is limited) demonstrates that despite the determinants for applying for pro-investment programs specified in this paper (such as the agricultural and rural development level), the applicants may be driven by a series of other unrelated factors (explained in lines 415-426).**

4. The discussion is extremely interesting, but is it not worthwhile to make recommendations for state policy, and perhaps the EU as well?

**In the paper we conclude that in order to ensure the development of rural areas in regions of less favourable agrarian structures where agriculture is currently unable to compete, it would be critical to enhance conditions for alternative types of economic activities. This calls for the EU intervention which is well-tailored to the local needs. The discussion on this point is in the last paragraph of the paper.**

5. It difficult to find the relationship between Figure 1 and 2, could you explain clearly?

**Figure 1 shows the spatial differentiation of the use of pro-investment measures in Poland, while Figure 2 shows selected aspects of Polish agrarian structure. One of the most important aspects of the research was to show the similarities between the location of high-high municipalities (Figure 1) and the share of farms with an area of more than 10 ha (Figure 2). The comparison of both figures was made in the Results section of the paper (lines 270-274). The dominant importance of structural factors in applying for EU funds has been also discussed in conclusions.**

Sincerely,

Ewa Kiryluk-Dryjska,

Arkadiusz Sadowski (corresponding author)

Barbara Więckowska
